# Acylated Aminooligosaccharides from the Yellow Sea *Streptomyces* sp. HO1518 as Both α-Glucosidase and Lipase Inhibitors

**DOI:** 10.3390/md18110576

**Published:** 2020-11-20

**Authors:** Jian-Lin Xu, Hai-Li Liu, Zhi-Feng Liu, Yu-Hong Ren, Yong Wang

**Affiliations:** 1 Key Laboratory of Synthetic Biology, CAS Center for Excellence in Molecular Plant Sciences, Institute of Plant Physiology and Ecology, Chinese Academy of Sciences, Shanghai 200032, China; xujianlin@cemps.ac.cn (J.-L.X.); liuzhifeng@cemps.ac.cn (Z.-F.L.); 2 University of Chinese Academy of Sciences, Beijing 100039, China; 3 State Key Laboratory of Bioreactor Engineering, East China University of Science and Technology, Shanghai 200237, China; yhren@ecust.edu.cn

**Keywords:** *Streptomyces* sp. HO1518, acylated aminooligosaccharides, α-glucosidase, α-amylase, sucrase, lipase

## Abstract

Three new acylated aminooligosaccharide (**1**–**3**), along with five known congeners (**4**–**8**), were isolated from the marine-derived *Streptomyces* sp. HO1518. Their structures were fully elucidated by extensive spectroscopic analysis, mainly based on 1D-selective and 2D TOCSY, HSQC-TOCSY, and HRESIMS spectrometry measurements, and by chemical transformations. All of the compounds were evaluated for their α-glucosidase and pancreatic lipase inhibitory activities. Among the isolates, *D*6-*O*-isobutyryl-acarviostatin II03 (**3**) and *D*6-*O*-acetyl-acarviostatin II03 (**8**), sharing acarviostatin II03-type structure, showed the most potent α-glucosidase and lipase inhibitory effects, far stronger than the antidiabetic acarbose towards α-glucosidase and almost equal to the anti-obesity orlistat towards lipase in vitro. This is the first report on inhibitory activities against the two major digestive enzymes for acylated aminooligosaccharides. The results from our investigation highlight the potential of acylated aminooligosaccharides for the future development of multi-target anti-diabetic drug.

## 1. Introduction

Type 2 diabetes mellitus (T2DM) is a frequent metabolic syndrome, characteristic of prolonged high levels of blood glucose. Chronic hyperglycemia would result in various complications, such as neuronal disorder, retinopathy, hypertension, kidney disease, and cardiovascular comorbidity, etc. [1,2,3]. Due to the global rising tide of obesity, intake of energy-dense diets, and sedentary lifestyles, the incidence and prevalence of T2DM has increased dramatically in recent years. It is estimated that 463 million people were living with T2DM and 4.2 million died from diabetes in 2019, with approximately 10% of global healthcare expenditure spent on diabetes and its complications, which placed immense economic pressures to the patients [4,5,6,7,8].

α-Glucosidases secreted from the intestinal chorionic epithelium mainly include two types of enzymes, α-amylases and disaccharidases. Functionally, α-amylases are capable of hydrolyzing complex polysaccharides into oligosaccharides by breaking the α-1,4-glycosidic bond in the non-reducing ends of polysaccharides, and the resulting oligosaccharides can be further catalyzed into glucose by the disaccharidases (sucrase, maltase, and isomaltase), which readily leads to blood glucose elevation in T2DM patients [9,10]. The competitive inhibition of these enzymes by α-glucosidase inhibitors is one of the most efficient therapeutic strategies for the treatment of T2DM since it can retard carbohydrate digestion and avoid excessive glucose absorption. The well-known acarbose, a nitrogen-containing *pseudo*-tetrasaccharide obtained from various actinomycetes, potently inhibits the α-glucosidases in vitro and in vivo, which is regarded as one of the most commonly used oral hypoglycemic drugs [11,12]. Additionally, T2DM can be largely attributed to the dysfunction of insulin-producing pancreatic islet β-cells, which is caused by the excess accumulation of lipids in the pancreas [2,13]. An increasing number of scientific evidences revealed that decreasing total pancreatic fat was associated with the improvement of the function of β-cells [14]. Pancreatic lipase (PL) plays a vital role in the hydrolysis of dietary lipids, which degrades triacylglycerols to free fatty acids and monoacylglycerols in the intestinal lumen [15]. Inhibiting PL contributes to reduce the lipid absorption and protect the pancreas, which will restore normal level of insulin secretion of the β-cells. Orlistat, a powerful PL inhibitor, is clinically widely used for the treatment of obesity. Although a number of natural metabolites, i.e., polyphenols, have recently been reported as both α-glucosidase and lipase inhibitors [16,17], none of them have the potency to be selected as hit molecules targeting T2DM, till now largely attributable to their relatively lower inhibitory activities towards either α-glucosidase or lipase when compared with acarbose or orlistat. Thus, it is imperative to search for more effective candidate compounds with dual properties against T2DM and obesity.

**Figure 1:**
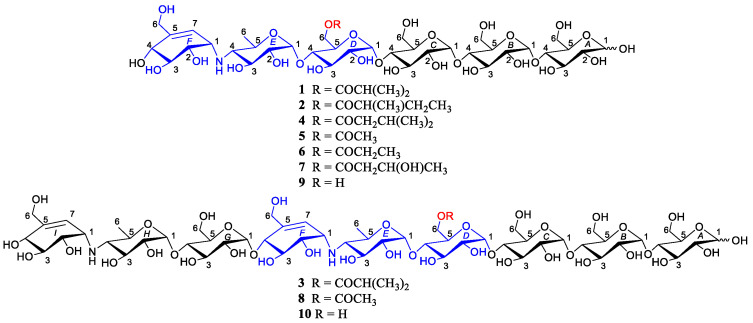
The structures of compounds **1**–**10**.

Actinomycetes, especially the genus *Streptomyces*, have been recognized as wealthy resource of pharmaceutically and industrially bioactive small molecules, including anticancer agents, antibiotics, and enzyme inhibitors [18,19]. Stimulated by the fruitful achievements from the *Streptomyces* species, our group has been dedicated to search for novel bioactive secondary metabolites from marine-derived actinomycetes [20,21,22,23]. Previously, we reported five new acylated oligosaccharides from the *Streptomyces* sp. HO1518, isolated from a sediment sample of Yellow Sea, among which *D*6-*O*-acetyl-acarviostatin II03 (**8**) was the most potent α-amylase inhibitor, with an IC_50_ value 540-fold stronger than acarbose [24]. Very recently, driven by the knowledge of α-glucosidase inhibition in diabetes and obesity [25,26], we tried to test **8** for its inhibitory capacity toward lipase enzyme. To our surprise, besides against α-glucosidase, **8** was found to exhibit conspicuous inhibition against lipase with an IC_50_ value of 2.00 μM, almost comparable to orlistat (IC_50_ = 0.58 μM) and far stronger than acarbose (IC_50_ = 207.57 μM), indicating that **8** and its structural analogues could be promising lead compounds in the development of antidiabetic agents. Moreover, careful reanalysis of the extract of the strain HO1518 using LC-MS suggested the presence of a handful of newly appeared molecular formula related to aminooligosaccharides. The above observation inspired our great interest to further carry out the search from the extract of *Streptomyces* sp. HO1518 for antidiabetic and anti-obesity agents. Then, large-scale refermentation of *Streptomyces* sp. HO1518 led to the isolation of three new acylated aminooligosaccharide congeners (**1**–**3**) and five known related compounds (**4**–**8**) (Figure 1). Herein, we describe their isolation, structural elucidation, and inhibitory activities against α-glucosidase and lipase.

## 2. Results and Discussion

### 2.1. Structure Determination of New Compounds

*D*6-*O*-isobutyryl-acarviostatin I03 (**1**) was isolated as white amorphous powder. The molecular formula was assigned as C_41_H_69_NO_29_ based on the positive mode HRESIMS (*m*/*z* 1040.4008 [M + H]^+^, calcd for C_41_H_70_NO_29_, 1040.4028), suggesting the presence of eight degrees of unsaturation. The IR spectrum (Figure S18) suggested characteristic absorption bands for hydroxyl (3350 cm^−1^) and carbonyl (1633 cm^−1^) groups. The ^13^C NMR in conjunction with the DEPT spectra (Figure S9) of **1** unlocked the existence of 41 carbon signals corresponding to three methyls, five sp^3^ methylenes, thirty sp^3^ methines, one sp^2^ methine, and two non-protonated carbons. An ester carbonyl (δ_C_ 180.1) and one olefinic bond (δ_C_ 123.7, 139.0) accounted for two out of eight degrees of unsaturation, which implies six rings should be present in **1**.

**Figure 2:**
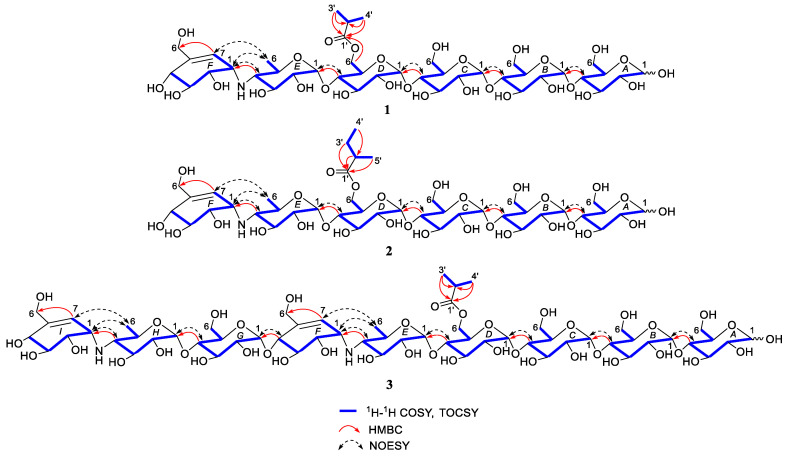
Key 2D NMR correlations of compounds **1**–**3**.

Careful comparison of NMR data of **1** with those of previously reported from *Streptomyces* sp. HO1518 [24], **1** was inferred as an aminooligosaccharide derivative, which was characterized by acarviosin moiety with d-glucose units attached in the reducing terminus through the glycosidic bond. The reducing terminal glucose unit (ring A) was confirmed by the typical protons of H-*A*1α, H-*A*1β and H-*A*2β (δ_H_ 5.24, 4.66 and 3.28), while the chemical shift of three low-field protons (δ_H_ 5.42, 3H, overlapped) allowed for the assignment of the anomeric protons of rings B–D [27]. Analysis of its 2D TOCSY spectrum (Figure S15) revealed the presence of two spin systems as depicted with bold blue lines in Figure 2: H-*E*1/H-*E*4/H_3_-*E6* and H-*F*1/H-*F*4/H_2_-*F*6/H-*F*7, which clearly revealed the presence of one acarviosin substructure (rings E and F) evidenced by the HMBC correlations from H-*E*4 (δ_H_ 2.46) to C-*F*1 (δ_H_ 56.0) and C-*F*2 (δ_H_ 72.9) as well as H-*F*1 (δ_H_ 3.53) to C-*E*4 (δ_H_ 65.0). When the independent protonic signals (δ_H_ 5.24, 4.66, 5.42, 4.44, 5.27, and 5.90) were selectively irradiated by the 1D-selective TOCSY experiments (Figures S2–S7), six self-spin systems of residues A-F were successfully acquired and their corresponding ^13^C NMR data could be confirmed on the basis of a comprehensive inspection of HSQC, HMBC, and HSQC-TOCSY spectra (Figures S10, S13 and S14). The remaining carbons resonated at δ_C_ 180.1, 33.8, 18.1, and 18.2 in **1** and were ascribed as an isobutyryl fragment, which was supported by the HMBC correlations from H_3_-3′ and H_3_-4′ to C-1′ and C-2′. The linkage of the isobutyryl group to the ring D was at C-*D*6 via an oxygen attributable to the down-field shifted methylene proton H_2_-*D*6 (δ_H_ 4.44, 4.23), as determined by the pivotal HMBC interaction from H-*D*6a to C-1′ (δ_C_ 180.1), indicating that the C-*D*6 hydroxyl group of **1** was esterified with the isobutyric acid.

**Figure 3:**
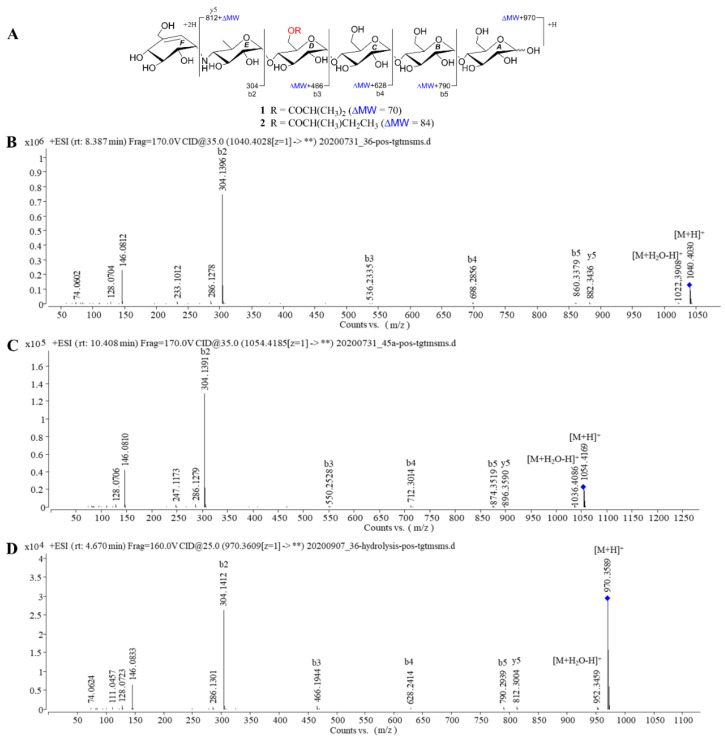
Positive HRESIMS/MS fragmentation and spectra of compounds **1**, **2** and **9**. (**A**) Positive-ion HRESIMS/MS fragmentation pattern of **1**, **2** and **9**; (**B**–**D**) HRESIMS/MS spectra of **1**, **2** and **9**.

The above deduction was further demonstrated by several of crucial fragment ions at *m*/*z* 860 (b5), 698 (b4), 536 (b3), and 304 (b2) observed in the positive ESIMS/MS spectrum (Figure 3), corresponding to the loss of one to four glucose units from **1**. Moreover, the ion at *m*/*z* 872 (y5) was produced by the cleavage of cyclohexitol–nitrogen bond in the non-reducing end of **1**, while the peak at *m*/*z* 1022 was correlated to the neutral loss of one water molecules. Given the coupling constants of the anomeric protons and the NOESY correlations (Figure 2), the configuration of the glycosidic bonds in **1** was determined as α-(1→4), the same as that of the model known precursor acarviostatin I03 (**9**) [28], which was further corroborated by the chemical correlation between **1** and **9**. Thus, the structure of compound **1** was completely assigned, as depicted in Figure 1.

Compound **2** was obtained as white amorphous powder with the molecular formula C_42_H_71_NO_29_, as determined by its HRESIMS data. The ^1^H and ^13^C NMR spectral data (Table 1) of **2** were almost in accordance with those of **1**, except for the possible replacement of the isobutyryl functionality in **1** by an additional 2-methyl-butyryl group [δ_C_ 179.8 (C-1′), δ_H_ 2.52 (H-2′), δ_C_ 40.9 (C-2′), δ_H_ 1.64, 1.51 (H_2_-3′), δ_C_ 26.4 (C-3′), δ_H_ 0.88 (H_3_-4′), δ_C_ 10.8 (C-4′), δ_H_ 1.14 (H_3_-5′), δ_C_ 15.7 (C-5′)] in **2**, which could be verified by the ^1^H-^1^H COSY cross peak of H_3_-5′/H-2′/H_2_-3′/H_3_-4′ as well as the HMBC correlations from H-2′, H_2_-3′ and H_3_-5′ to C-1′ and H_3_-4′ to C-2′ (Figure 2). Similarly, four proton signals in the residue D (δ_H_ 3.93, 3.63, 4.01, 4.20 and 4.44) of **2** were slightly low-field shifted by comparing with **9**, which suggested that the -OH at C-*D*6 was esterified with the five-carbon acyl group in **2**. Further support for the proposed assignment was evidenced by the HMBC correlation of H-*D*6a (δ_H_ 4.44) to C-1′ (δ_C_ 179.8) and the ESIMS/MS peaks at *m*/*z* 896 (y5), 874 (b5), 712 (b4), 550 (b3), and 304 (b2). The configuration of the glycosidic bonds in **2** was deduced to be the same as that of **1** based on the similar ^1^H-^1^H coupling constants of the anomeric protons between **1** and **2**, which could be reconfirmed by the NOESY spectrum (Figure S33). Meanwhile, the alkaline hydrolysis method was applied to determine the absolute configuration of 2-methyl-butyryl side chain in **2** [29]. Unfortunately, substantial tentative efforts to acquire its related esterification products were failed mainly attributable to the limit amount of **2**. Therefore, the absolute configuration of the acyl unit remains undetermined.

**Figure 4:**
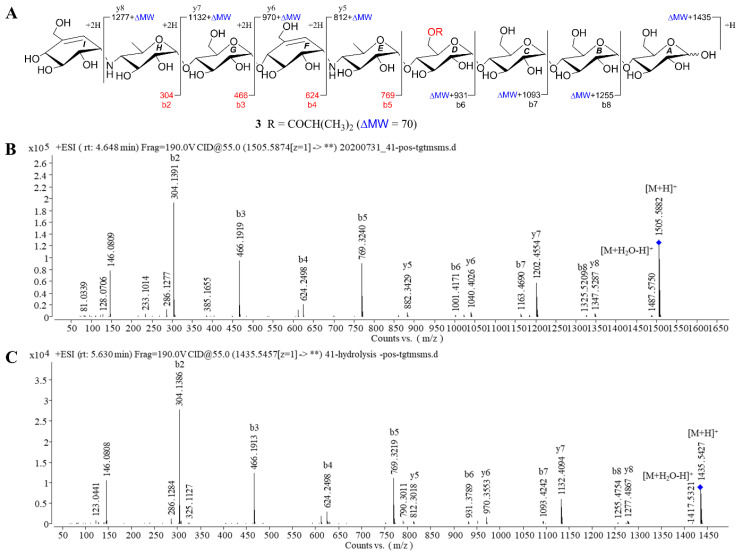
Positive HRESIMS/MS fragmentation and spectra of compounds **3** and **10**. (**A**) Positive-ion HRESIMS/MS fragmentation pattern of **3** and **10**; (**B**,**C**) HRESIMS/MS spectra of **3** and **10**.

Compound **3**, white amorphous powder, was assigned as C_60_H_100_N_2_O_41_ with the inference to its HRESIMS data (*m*/*z* 1505.5892 [M + H]^+^, calcd for 1505.5874), implying 12 degrees of hydrogen deficiency. Careful inspection of the NMR spectroscopic data between **1** and **3** revealed that **3** possessed an analogous structure to **1**, except for the presence of the second *pseudo*-trisaccharide core in **3**. Among the observed carbon resonances in the ^13^C spectrum (Figure S47), signals at δ_C_ 20.2, 58.8, 63.3, 64.4, 67.8, 72.4, 73.7, 73.8, 74.0, 74.2, 75.4, 75.6, 75.8, 76.0, 76.3, 100.4, 102.7, 126.6, and 141.8 (Table 2) were deduced to be affiliated to an additional *pseudo*-trisaccharide substructure in the non-reducing end, which was consistent with 465 mass unit more than that of **1**. These data were confirmed by the COSY cross-peaks of H-*G*1/H-*G*2/H-*G*3/H-*G*4/H-*G*5/H_2_-*G*6, H-*H*1/H-*H*2/H-*H*3/H-*H*4/H-*H*5/H_2_-*H*6, and H-*I*7/H-*I*1/H-*I*2/H-*I*3/H-*I*4 and the HMBC correlations from H-*G*4 to C-*H*1, H-*H*4 to C-*I*1 as well as H-*I*7 to C-*I*6 (Figure 2). The location of the isobutyryloxyl group was assigned at C-*D*6 due to the diagnostic ESIMS/MS fragment ions at *m*/*z* 1347 (y8), 1202 (y7), 1040 (y6), 1001 (b6), 882 (y5), and 769 (b5) (Figure 4). Likewise, the almost identical coupling constants of the anomeric protons in conjunction with the NOESY correlations (Figure S54) suggested the glycosidic bonds of **3** to be α-(1→4), further evidenced by the chemical conversation between **3** and co-occurring known acarviostatin II03 **10 [24]**. Consequently, compound **3** was identified and named *D*6-*O*-isobutyryl-acarviostatin II03.

Five known congeners were identified as isovalertatin M03 (**4**) [30], *D*6-*O*-acetyl-acarviostatin I03 (**5**) [24], *D*6-*O*-propionyl-acarviostatin I03 (**6**) [24], *D*6-*O*-β-hydroxybutyryl-acarviostatin I03 (**7**) [24] and *D*6-*O*-acetyl-acarviostatin II03 (**8**) [24] by comparison of spectroscopic data with literature values.

Taken together, the structure elucidations of three new aminooligosaccharide derivatives **1**–**3** revealed that their major differences were attributed to the number of *pseudo*-trisaccharide core(s) and acyl group. Generally speaking, aminooligosaccharides show high sensitivity to positive-ion MS/MS technique, owing to the presence of the readily protonated amine residues. The extensive MS study enables one to obtain all of primary and secondary fragment ions of oligosaccharides, which is conducive to identifying their structures [31,32]. In the positive ion mode ESIMS/MS spectra, the bi and yj fragment ion peaks are corresponding to glycosidic bond dissociation of protonated aminooligosaccharides, and every glycosidic bond could be dissociated to some extent [28]. As referring to **1** and **2**, their most abundant fragment ion at *m*/*z* 304 (b2) was identical to the known acarviostatin I03 (**9**), while the signals at *m*/*z* b3-b5 and y5 in **1** and **2** were 70 and 84 mass units more than those of **9**, respectively, indicating that the hydroxyl group at C-*D*6 of the two oligomers was esterified with the four- or five-carbon acyl units, respectively (Figure 3). As for **3**, the molecular weight of **3** had 465 mass units higher than that of **1**, which was indicative of the existence of the repeated *pseudo*-trisaccharide substructure. A careful comparison of ESIMS/MS spectra between **3** and known acarviostatin II03 (**10**) revealed that the ion peaks at *m*/*z* b5-b8 in **3** were consistent with those of its deacyl product **10**, whereas the other fragmental ion signals in **3** (b6-b8 and y5-y8) was 70 Da more than that of **10**, which was characteristic for the isobutyryl group (Figure 4). Interestingly, this isobutyryl substituent was reacted with the hydroxyl group of C-*D*6 in **3**, the same as those of **1** and **2**. Furthermore, the alkaline hydrolysis of **1**–**3** was carried out, which was used to confirm the aforementioned structural deduction. As expected, when **1**–**3** were treated individually with ammonium hydroxide in methanol, two corresponding precursors **9** and **10** were yielded. Based on these reliable results, the structures of **1**–**3** were unequivocally determined.

### 2.2. Inhibitory Activities Against α-Glucosidase and Pancreatic Lipase

As mentioned above, T2DM is a complicated metabolic disease, which is closely associated with disturbances of glycose and lipid metabolism. The inhibitory activities against key digestive enzymes involved in the breakdown of polysaccharides and fat, such as α-glucosidase and lipase, have been recognized as effective therapeutics in the management of blood glucose concentration in diabetic patient. Therefore, we evaluated compounds **1**–**8** for their inhibitory activity against α-glucosidase and pancreatic lipase.

For α-glucosidase inhibitory activity assay, two types of enzymes including porcine pancreatic α-amylase (PPA) and sucrose (disaccharidase) were chosen in this study. Firstly, **1**–**4** were evaluated for their inhibitory effects on PPA with acarbose as the positive control. Similar to **5**–**8** [24], **1**–**4** also caused remarkable inhibition of PPA with the IC_50_ values ranging from 0.04 to 0.34 μM as shown in Table 3, of which *D*6-*O*-isobutyryl-acarviostatin II03 (**3**) was 77-fold stronger than acarbose (3.80 μM). Subsequently, all of the tested isolates (**1**–**8**) showed more potential sucrase inhibition ability than acarbose with the IC_50_ values ranging from 0.41 to 9.34 μM. Amongst them, *D*6-*O*-2-acetyl-acarviostatin II03 (**8**) exhibited the strongest activity against sucrase, with an IC_50_ value 27 times more effective than acarbose. In addition, the pancreatic lipase (PL) inhibitory activities of **1**–**8** were also performed. **1**–**8** displayed considerable inhibitory effect against PL with the IC_50_ values ranging from 0.82 to 19.7 μM, while acarbose only showed extremely weak activity with an IC_50_ value of more than 200 μM. Notably, the inhibitory potential of **3** and **8** toward lipase was nearly comparable to the anti-obesity agent orlistat. To our knowledge, this is the first report on inhibitory activities against the two major digestive enzymes for acylated aminooligosaccharides.

### 2.3. Structure-Activity Relationships (SAR) of Acylated Aminooligosaccharides

Preliminary SAR demonstrated that the number of the *pseudo*-trisaccharide core and the acyloxyl unit at C-*D*6 played a pivotal role in the inhibition potency of digestive enzymes including α-glucosidase and lipase enzymes. Compound **3** sharing the identical isobutyryl group as **1** showed stronger inhibitory capacity against all tested enzymes than **1**, suggesting that the increase in the *pseudo*-trisaccharide core was beneficial to both α-glucosidase and lipase inhibitory activities. When the four- or five-carbon acyl side chains were reduced to short chains such as acetyl or propionyl groups, the lipase inhibitory activity of the oligosaccharides **5** and **6** were significantly decreased as compared to **1**, **2**, **4,** and **7**, which revealed that the introduction of the long-chain acyl group seemed to be favorable for the biological properties towards lipase. However, increasing the acyl chain length would pose completely the opposite effect in the sucrase inhibition assay, leading to weaker suppressing activity, as referring to six compounds **1**, **2,** and **4**–**7**. All isolates displayed the strongest PPA inhibition in three digestive enzymes inhibition assay, indicating that aminooligosaccharides might have a better binding affinity against α-amylase comparing to sucrase and lipase.

Although eight acylated aminooligosaccharides exhibited excellent inhibitory efficacy against both α-glucosidase and lipase, the information with respect to the inhibition of these metabolic enzymes by oligosaccharides remains unclear. Additionally, the biosynthetic speculation of acylated aminooligosaccharides seemed to be an intractable challenge owing to their perplexing backbone with multiple glucose or *pseudo*-glucose units along with an intriguing acyloxyl group only fixed at C-*D*6. Mechanistic details including the binding energy for interaction molecules or ligands within the active site of each enzyme using docking study as well as their entire biosynthetic pathway related to the rich skeletons and the rare acyl side chains will be revealed in the further studies.

## 3. Materials and Methods

### 3.1. General Experimental Procedures

Optical rotations were carried out on an Anton Paar MCP-500 spectropolarimeter (Anton Paar, Graz, Austria) at 20 °C. UV spectra were obtained on a JASCO V-550 UV/VIS spectrophotometer (Jasco Corporation, Tokyo, Japan). IR data were measured using a FT-IR Vertex 70 v spectrometer (Bruker, Fällanden, Switzerland). The 1D and 2D NMR spectra were acquired using a Bruker Avance 500 MHz spectrometer with TMS as an internal standard (Bruker, Fällanden, Switzerland). HRESIMS data were collected on a Thermo Q Exactive high resolution mass spectrometer (Thermo Fisher Scientific, Waltham, MA, USA). ESIMS/MS data were recorded on an Agilent Q-TOF 6545 mass spectrometer (Agilent Technologies, Santa Clara, CA, USA). MCI gel CHP20/P120 (Mitsubishi Chemical Corporation, Tokyo, Japan) and SiliaSphere C_18_ (50 µm, Silicycle, QuébecK, QC, Canada) were used for column chromatography (CC). TLC analysis was carried out on silica gel plates (Yantai Chemical Inst., Yantai, China). Thermo ultimate 3000 (Thermo Fisher Scientific, Waltham, MA, USA) equipped with Alltech 3300 ELSD detector and VWD detector was used for HPLC. Preparative HPLC was performed using a SilGreen C_18_ column (250 × 20 mm, 5 μm, 12 nm, Greenherbs CO., Ltd., Beijing, China); while semi-preparative HPLC was performed utilizing a TSK-gel 100 V C_18_ column (250 × 10 mm, 5 μm, 12 nm, Tosoh Corporation, Tokyo, Japan). All solvents were of analytical grade (Sinopharm Chemical Reagent Co., Ltd., Beijing, China). Porcine pancreatic α-amylase and pancreatic lipase were purchased from Sigma Aldrich Co. (St Louis, MO, USA), sucrase and acarbose were obtained from Shanghai yuanye Bio-Technology Co., Ltd. (Shanghai, China). Orlistat was bought from Shanghai xushuo Bio-Technology Co., Ltd. (Shanghai, China).

### 3.2. Strain Isolation and Identification

The bacteria *Streptomyces* sp. HO1518 was isolated from a sediment sample collected in summer 2010, from the Rizhao coastal area, Shandong Province of China, at a depth of 50–100 m. The strain HO1518 shows 99% similarity to *Streptomyces fradiae* (Accession No. KP698740.1) based on 16S rDNA sequence analysis. This strain (Voucher Specimen No. M2018176) is preserved at the China Center for Type Culture Collection (CCTCC), Wuhan University.

### 3.3. Fermentation, Extraction and Isolation

The bacterial strain HO1518 was inoculated on MS solid growth medium (20 g/L soybean flour, 20 g/L mannitol, and 20 g/L agar powder) and cultivated at 28 °C for a week. The spores of HO1518 were inoculated into 400 mL of TSB liquid medium in the ten 2 L-Erlenmeyer flasks, which were cultivated at 28 °C and 200 rpm as seed cultures. After 2 days of fermentation, 3.5 L of the culture was transferred to 35 L CSM liquid medium containing 10 g/L cottonseed meal, 10 g/L soluble starch, 12 g/L glucose, 5 g/L corn steep liquor, and artificial seawater (g/L: NaCl 24.48, Na_2_SO_4_ 3.92, KCl 0.66, SrCl_2_·6H_2_O 0.04, MgCl_2_·6H_2_O 4.98, CaCl_2_ 0.95, NaHCO_3_ 0.19, H_3_BO_3_ 0.026, and NaF 0.004), pH 7.2. The entire culture was incubated in a fermenter at 28 °C for 7 days. Then, the 35 L fermented broth was filtered to remove mycelia and the secondary metabolites scattered in the supernatant were absorbed with XAD-16 resins, which were further eluted with anhydrous ethanol to obtain a crude extract. The ethanol extract (10.2 g) was separated into 6 fractions (Frs. 1–6) on a C_18_ reverse-phase (RP) silica gel column by step gradient elution with MeOH/H_2_O (5%–100%, *v/v*).

Fr. 1 (4.2 g) was chromatographed over RP silica gel column using gradient elution with MeOH/H_2_O (5–100%, *v/v*) to obtain eleven subfractions (Frs. 1A–1K). Fr. 1G was purified by preparative RP HPLC system equipped with a SilGreen C_18_ column (MeCN/H_2_O, 8 mL/min, 0~50 min, 5:95→25:75; 50.1~70 min, 25:75→50:50) to produce two major parts, which were further purified on semi-preparative RP HPLC equipped with a TSK-gel 100 V C_18_ column (MeOH/H_2_O, 3 mL/min, 18:82) to yield **5** (5.0 mg, t*_R_* 24.7 min) and **7** (4.3 mg, t*_R_* 32.7 min), respectively. Fr. 1I was chromatographed on MCI gel column and separated by semi-preparative RP HPLC (MeOH/H_2_O, 3 mL/min, 18:82) to afford **8** (50.2 mg, t*_R_* 28.7 min). Fr. 1K was separated by MCI gel CC and semi-preparative RP HPLC (MeOH/H_2_O, 3 mL/min, 18:82) to afford **6** (40.2 mg, t*_R_* 38.7 min).

Fr. 2 (3.9 g) was subjected to MCI gel CC, eluting with MeOH/H_2_O (5–100%, *v/v*) to afford seven subfractions (Frs. 2A–G). Fr. 2F was repurified by preparative RP HPLC system to produce two major parts, which were further purified on semi-preparative RP HPLC (MeOH/H_2_O, 3 mL/min, 26:74) to obtain **1** (10.9 mg, t*_R_* 28.7 min) and **3** (10.2 mg, t*_R_* 30.6 min), respectively. Fr. 2G was further purified by preparative RP HPLC system (MeCN/H_2_O, 8 mL/min, 0~50 min, 5:95→25:75; 50.1~70 min, 25:75→50:50) and semi-preparative RP HPLC (MeOH/H_2_O, 3 mL/min, 26:74) to acquire **2** (3.2 mg, t*_R_* 17.6 min) and **4** (15.6 mg, t*_R_* 18.8 min), respectively.

*D*6-*O*-isobutyryl-acarviostatin I03 (**1**): White amorphous powder, ${\left[\alpha \right]}_{D}^{25}$ + 366.1 (*c* 1.27, H_2_O). UV (H_2_O) end absorption; IR *ν*_max_ 3350, 1633, 1353, 1151, 1020 cm^−1^. ^1^H (500 MHz) and ^13^C (125 MHz) NMR spectroscopic data, see Table 1; positive ESIMS: *m*/*z* 1040 [M + H]^+^; HRESIMS: *m*/*z* 1040.4008 [M + H]^+^ (calcd for C_41_H_70_NO_29_, 1040.4028).

*D*6-*O*-2-methyl-butyryl-acarviostatin I03 (**2**): White amorphous powder, ${\left[\alpha \right]}_{D}^{25}$ + 154.0 (c 0.60, H_2_O). UV (H_2_O) end absorption; IR *ν*_max_ 3343, 1634, 1152, 1023 cm^−1^. ^1^H (500 MHz) and ^13^C (125 MHz) NMR spectroscopic data, see Table 1; positive ESIMS: *m*/*z* 1054 [M + H]^+^; HRESIMS: *m*/*z* 1054.4193 [M + H]^+^ (calcd for C_42_H_72_NO_29_, 1054.4184).

*D*6-*O*-isobutyryl-acarviostatin II03 (**3**): White amorphous powder, ${\left[\alpha \right]}_{D}^{25}$ + 99.3 (c 0.42, H_2_O). UV (H_2_O) end absorption; IR *ν*_max_ 3301, 1637, 1152, 1024 cm^−1^. ^1^H (500 MHz) and ^13^C (125 MHz) NMR spectroscopic data, see Table 2; positive ESIMS: *m*/*z* 1505 [M + H]^+^; HRESIMS: *m*/*z* 1505.5892 [M + H]^+^ (calcd for C_60_H_101_N_2_O_41_, 1505.5874).

### 3.4. Conversion of Compounds 1 and 2 to 9 and 3 to 10

One milligram of compounds **1**–**3** were dissolved individually in 2 mL of 0.1 M ammonium hydroxide in 70% MeOH-H_2_O. The mixtures were stirred at room temperature (RT) for 30 h, and the hydrolysis products were analyzed via LC-MS technique. The analysis indicated that both **1** and **2** had been hydrolyzed into common precursor **9**, while **3** had been changed into precursor **10**. The structure of their deacyl-products (**9** and **10**) was identified by comparing of their ^1^H NMR spectra (Figures S58 and S59) with those reported in the literature [28].

### 3.5. α-Glucosidase Inhibition Assay

#### 3.5.1. Porcine Pancreatic α-Amylase (PPA) Inhibition Assay

The PPA inhibitory activities of compounds **1**–**4** were performed based on the previously reported method. Acarbose was used as the positive control [24].

#### 3.5.2. Sucrase Inhibition Assay

The sucrase inhibition assay was performed according to the method outlined by Honda et al. with some modifications [33]. In brief, 10 µL of enzyme solution (100 U/mL) and 30 µL of test sample solution with appropriate concentration (dissolved in distilled water) were added into the 96-well plates, and were incubated for 10 min at 37 °C. The reaction was conducted by addition of 100 µL of sucrose solution (60 mM, dissolved in 0.1 M phosphate buffer solution). Then, the mixture was added to 200 µL of 3,5-dinitrosalysilic acid, which was heated in boiling water for 5 min to stop the reaction. The absorbance of each tested compound was measured at 540 nm. The inhibition rates were calculated using the following equation. All experiments were measured in triplicate, and a logarithmic regression curve was established to calculate IC_50_ values.

(1) $$\mathrm{Inhibition}\;\%\;=\;\frac{A_{\mathrm{control}\;}{-\;A}_{\mathrm{sample}}}{A_{\mathrm{control}}}\;\times \;100$$

where A_control_ represents the absorbance of the mixture of sample solution, sucrose solution 3,5-dinitrosalysilic acid and enzyme solution; while the A_sample_ is the absorbance of the mixture of phosphate buffer, sucrose solution 3,5-dinitrosalysilic acid and enzyme solution.

### 3.6. Pancreatic Lipase (PL) Inhibition Assay

This assay was carried out using *p*-nitrophenyl laurate (*p*NP laurate) method with slight modification [34]. In detail, 150 µL lipase solution (10 mg/mL, dissolved in the 0.1 M Tris buffer) was mixed with 50 µL tested sample solution (inhibitors) in a 1.5 mL centrifuge tube, then 350 µL 0.1 M Tris buffer (pH = 8.2) was added and pre-incubated. After the addition of 450 µL *p*NP laurate (substrate), the reaction was incubated for 30 min at 37 °C. The mixtures were heated in boiling water for 5 min to terminate the reaction. After cooling to RT, the tube was centrifuged at 12,000 rpm for 3 min. A portion of 100 µL of mixture was added into a 96-well plate, which was measured at 405 nm. The percentage of inhibitory activities was calculated using the formula below. All data were performed in triplicate, and a logarithmic regression curve was established to calculate IC_50_ values.

(2) $$\mathrm{Inhibition}\%\;=\;\left[1\;-\;\left(\frac{A_{\mathrm{sample}}{\;-\;A}_{\mathrm{blank}}}{A_{\mathrm{test}}\;{-\;A}_{\mathrm{control}}}\right)\right]\;\times \;100$$

where A_sample_ is the absorbance of the mixture of sample solution, *p*NP solution and enzyme solution; A_blank_ is the absorbance of the mixture of sample solution and *p*NP solution without enzyme; A_test_ is the absorbance of the mixture of Tris buffer, *p*NP solution and enzyme solution; A_control_ is the absorbance of the mixture of Tris buffer and *p*NP solution without enzyme.

## 4. Conclusions

In summary, three new acarviosin-containing oligosaccharides, *D*6-*O*-isobutyryl-acarviostatin I03 (**1**), *D*6-*O*-2-methyl-butyryl-acarviostatin I03 (**2**) and *D*6-*O*-isobutyryl-acarviostatin II03 (**3**), together with five known analogues (**4**–**8**), were isolated from the marine actinomycete *Streptomyces* sp. HO1518. The structures of all new compounds were fully elucidated by a combination of NMR data, HRESIMS, as well as chemical conversion. Compounds **1**–**8** exhibited conspicuous inhibitory activities against both α-glucosidase and lipase enzymes under the low micromolar concentrations, among which **3** and **8** with acarviostatin II03-type structure are the most promising dual α-glucosidase and lipase inhibitors for the treatment of T2DM. To the best of our knowledge, this is the first report of the inhibitory effects of acylated aminooligosaccharides on the two major digestive enzymes. In addition, the SAR of **1**–**8** was summarized, which highlighted that the number of the *pseudo*-trisaccharide unit and the length of C-*D*6 acyl substituent might exert conspicuous influence on their enzyme inhibitory activities. This study not only enriched the chemical diversity of aminooligosaccharides produced by the genus *Streptomyces*, but also provided new candidate molecules for further scientific research towards multi-target anti-diabetic drug discovery.

## Figures and Tables

**Table 1:** ^1^H (500 MHz) and ^13^C (125 MHz) NMR Data for **1** and **2** in D_2_O.

No.	1		2	
	δ_C_	δ_H_ (*J* in Hz)	δ_C_	δ_H_ (*J* in Hz)
A1α	91.9, CH	5.24, d (3.5)	91.9, CH	5.21, d (3.5)
A2α	71.3, CH	3.58, m	71.3, CH	3.58, m
A3α	73.1, CH	3.98, m	73.1, CH	3.96, m
A4α	76.2, CH	3.66, m	76.2, CH	3.69, m
A5α	69.9, CH	3.98, m	69.9, CH	3.96, m
A6α	60.4, CH_2_	3.84, m	60.4, CH_2_	3.88, m
A1β	95.8, CH	4.66, d (8.0)	95.8, CH	4.63, d (8.0)
A2β	74.0, CH	3.28, m	74.0, CH	3.25, m
A3β	77.0, CH	3.78, t (9.0)	76.9, CH	3.75, m
A4β	76.8, CH	3.66, m	76.8, CH	3.63, m
A5β	74.5, CH	3.61, m	74.5, CH	3.61, m
A6β	60.4, CH_2_	3.91, m	60.4, CH_2_	3.89, m
B1	99.4, CH	5.42, overlapped, (3.5)	99.4, CH	5.38, overlapped, (3.5)
B2	71.5, CH	3.64, m	71.6, CH	3.60, m
B3	73.2, CH	3.96, m	73.2, CH	3.94, m
B4	76.8, CH	3.67, m	76.8, CH	3.64, m
B5	71.1, CH	3.84, m	71.1, CH	3.82, m
B6	60.5, CH_2_	3.84, m	60.5, CH_2_	3.82, m
C1	99.5, CH	5.42, overlapped, (3.5)	99.5, CH	5.38, overlapped, (3.5)
C2	71.6, CH	3.64, m	71.5, CH	3.60, m
C3	73.3, CH	3.96, m	73.3, CH	3.94, m
C4	77.0, CH	3.67, m	77.0, CH	3.64, m
C5	71.2, CH	3.84, m	71.2, CH	3.82, m
C6	60.7, CH_2_	3.84, m	60.7, CH_2_	3.82, m
D1	99.6, CH	5.41, overlapped, (3.5)	99.6, CH	5.38, overlapped, (3.5)
D2	72.2, CH	3.65, m	72.2, CH	3.61, m
D3	73.3, CH	3.95, m	73.3, CH	3.93, m
D4	78.2, CH	3.65, m	78.4, CH	3.63, m
D5	69.0, CH	4.06, d (11.6)	69.0, CH	4.01, m
D6a	63.5, CH_2_	4.44, d (11.6)	63.4, CH_2_	4.44, d (10.0)
D6b		4.23, dd (11.6, 5.0)		4.20, d (10.0)
E1	100.7, CH	5.27, d (3.4)	100.7, CH	5.23, d (3.4)
E2	71.3, CH	3.59, m	71.3, CH	3.56, m
E3	72.7, CH	3.62, m	72.8, CH	3.60, m
E4	65.0, CH	2.46, m	65.0, CH	2.43, m
E5	69.8, CH	3.73, m	69.8, CH	3.72, m
E6	17.3, CH_3_	1.31, d (6.0)	17.3, CH_3_	1.29, d (6.6)
F1	56.0, CH	3.53, m	56.0, CH	3.52, m
F2	72.9, CH	3.65, m	73.0, CH	3.62, m
F3	73.0, CH	3.75, m	73.0, CH	3.73, m
F4	70.8, CH	4.06, d (4.6)	70.9, CH	4.01, d (4.8)
F5	139.0, C		139.0, C	
F6a	61.6, CH_2_	4.23, brd (14.1)	61.6, CH_2_	4.20, brd (14.2)
F6b		4.12, brd (14.1)		4.09, brd (14.2)
F7	123.7, CH	5.90, s	123.7, CH	5.87, s
1′	180.1, C=O		179.8, C=O	
2′	33.8, CH	2.71, m	40.9, CH	2.52, m
3′	18.1, CH_3_	1.19, d (3.8)	26.4, CH_2_	1.51, m
				1.64, m
4′	18.2, CH_3_	1.19, d (3.8)	10.8, CH_3_	0.88, t (7.0)
5′			15.7, CH_3_	1.14, d (7.0)

**Table 2:** ^1^H (500 MHz) and ^13^C (125 MHz) NMR data for **3** in D_2_O.

No.	3		No.	3	
	δ_C_	δ_H_ (*J* in Hz)		δ_C_	δ_H_ (*J* in Hz)
A1α	94.8, CH	5.26, d (3.3)	E5	72.6, CH	3.73, m
A2α	74.2, CH	3.56, m	E6	20.2, CH_3_	1.33, d (6.0)
A3α	76.1, CH	4.18, m	F1	57.9, CH	3.56, m
A4α	79.6, CH	3.72, m	F2	73.5, CH	3.82, m
A5α	72.8, CH	3.93, m	F3	73.6, CH	4.15, m
A6α	63.3, CH_2_	3.76, m	F4	79.0, CH	4.07, m
A1β	98.6, CH	4.68, d (7.8)	F5	139.3, C	
A2β	76.8, CH	3.30, dd (9.0, 7.8)	F6a	64.8, CH_2_	4.25, m
A3β	79.1, CH	3.79, m	F6b		4.15, m
A4β	79.7, CH	3.66, m	F7	129.2, CH	6.01, d (4.5)
A5β	77.4, CH	3.60, m	G1	100.4, CH	5.40, d (3.5)
A6β	63.4, CH_2_	3.92, m	G2	76.0, CH	3.65, m
B1	102.4, CH	5.43, d (3.3)	G3	76.3, CH	3.92, m
B2	74.4, CH	3.64, m	G4	73.7, CH	3.63, m
B3	76.2, CH	3.97, m	G5	74.0, CH	3.94, m
B4	79.9, CH	3.69, m	G6	63.3, CH_2_	3.82, m
B5	74.0, CH	3.86, m	H1	102.7, CH	5.34, d (3.3)
B6	63.5, CH_2_	3.86, m	H2	74.2, CH	3.65, m
C1	102.4, CH	5.43, d (3.3)	H3	75.4, CH	3.65, m
C2	74.3, CH	3.64, m	H4	67.8, CH	2.49, m
C3	76.2, CH	3.97, m	H5	72.4, CH	3.81, m
C4	79.7, CH	3.69, m	H6	20.2, CH_3_	1.37, d (6.0)
C5	74.1, CH	3.86, m	I1	58.8, CH	3.56, t (5.0)
C6	65.3, CH_2_	3.86, m	I2	75.6, CH	3.69, m
D1	102.2, CH	5.43, d (3.3)	I3	75.8, CH	3.79, m
D2	73.9, CH	3.68, m	I4	73.8, CH	4.07, m
D3	76.0, CH	3.97, m	I5	141.8, C	
D4	81.0, CH	3.68, m	I6a	64.4, CH_2_	4.25, m
D5	71.8, CH	4.07, m	I6b		4.15, m
D6a	66.3, CH_2_	4.46, m	I7	126.6, CH	5.93, d (2.5)
D6b		4.25, q (5.4)	1′	182.9, C=O	
E1	103.6, CH	5.29, d (3.3)	2′	36.7, CH	2.73, m
E2	74.1, CH	3.55, m	3′	21.0, CH	1.20, d (2.7)
E3	75.8, CH	3.57, m	4′	21.1, CH	1.22, d (2.7)
E4	67.1, CH	2.49, m			

**Table 3:** The inhibitory activities of **1**–**8** against PPA, sucrase, and PL.

Compounds	IC_50_ Values (μM) ^a^		
	Against PPA	Against Sucrase	Against PL
**1**	0.22 ± 0.04	5.50 ± 0.09	2.16 ± 0.60
**2**	0.15 ± 0.01	3.12 ± 0.10	4.92 ± 0.20
**3**	0.04 ± 0.01	1.45 ± 0.07	0.82 ± 0.08
**4**	0.34 ± 0.07	9.34 ± 0.12	3.20 ± 0.10
**5**	n.t. ^b^	2.98 ± 0.10	19.70 ± 1.00
**6**	n.t. ^b^	2.36 ± 0.12	15.90 ± 0.60
**7**	n.t. ^b^	3.68 ± 0.13	8.10 ± 0.60
**8**	n.t. ^b^	0.41 ± 0.05	2.00 ± 0.18
acarbose	3.80 ± 0.15	11.27 ± 0.20	207.57 ± 9.77
orlistat	n.t.	n.t.	0.58 ± 0.14

^a^ Values are expressed as the mean ± SD; ^b^ the IC_50_ values against PPA for **5**–**8** have been reported in our previous study [24]; n.t. means not tested.

## Supplementary Materials

The following are available online at https://www.mdpi.com/1660-3397/18/11/576/s1, Figures S1–S57: HRESIMS, IR, UV, and 1D and 2D NMR spectra of compounds **1**–**3**; Figures S58–S59: ^1^H NMR spectra of compounds **9** and **10**.
