# Supplementary material for: Acylated Aminooligosaccharides from the Yellow Sea Streptomyces sp. HO1518 as Both α-Glucosidase and Lipase Inhibitors
Source: Mar Drugs. 2020 Nov 20;18(11):576. doi: 10.3390/md18110576 (PMC7699847; doi:10.3390/md18110576)
Supplement: Supplementary file 1 [file marinedrugs-18-00576-s001.pdf]

Supplementary Information

Acylated aminooligosaccharides from the Yellow Sea *Streptomyces* sp. HO1518 as both α-glucosidase and lipase inhibitors

Jianlin Xu[1,2,3], Haili Liu[1,*], Zhifeng Liu[1,2], Yuhong Ren[3], Yong Wang[1,*]

[1] Key Laboratory of Synthetic Biology, CAS Center for Excellence in Molecular Plant Sciences, Institute of Plant Physiology and Ecology, Chinese Academy of Sciences, Shanghai 200032, China

[2] University of Chinese Academy of Sciences, Beijing 100039, China

[3] State Key Laboratory of Bioreactor Engineering, East China University of Science and Technology, Shanghai 200237, China

[*] Corresponding authors.

E-mail addresses: hlliu@cemps.ac.cn (H.-L.L.); yongwang@cemps.ac.cn (Y.W.)

Table of Contents

Figure S1. ^{1}H NMR spectrum of compound **1** (500 MHz, D_2O).. 4
Figure S2. 1D-selective TOCSY spectrum of compound **1** (500 MHz, D_2O, excitation at δ 5.24, H-**A**1α). 5
Figure S3. 1D-selective TOCSY spectrum of compound **2** (500 MHz, D_2O, excitation at δ 4.66, H-**A**1β).. 6
Figure S4. 1D-selective TOCSY spectrum of compound **1** (500 MHz, D_2O, excitation at δ 5.42, H-**B**1, H-**C**1, and H-**D**1)... 7
Figure S5. 1D-selective TOCSY spectrum of compound **1** (500 MHz, D_2O, excitation at δ 4.44, H-**D**6a)... 8
Figure S6. 1D-selective TOCSY spectrum of compound **1** (500 MHz, D_2O, excitation at δ 5.27, H-**E**1).. 9
Figure S7. 1D-selective TOCSY spectrum of compound **1** (500 MHz, D_2O, excitation at δ 5.90, H-**F**7).. 10
Figure S8. ^{13}C NMR spectrum of compound **1** (125 MHz, D_2O)... 11
Figure S9. DEPT-135 spectrum of compound **1** (125 MHz, D_2O).. 12
Figure S10. HSQC spectrum of compound **1** (500 MHz, D_2O). 13
Figure S11. ^{1}H-^{1}H COSY spectrum of compound **1** (500 MHz, D_2O)................................. 14
Figure S12. 2D-TCOSY spectrum of compound **1** (500 MHz, D_2O)................................... 15
Figure S13. HSQC-TCOSY spectrum of compound **1** (500 MHz, D_2O)............................. 16
Figure S14. HMBC spectrum of compound **1** (500 MHz, D_2O). 17
Figure S15. NOESY spectrum of compound **1** (500 MHz, D_2O).. 18
Figure S16. HRESIMS spectrum of compound **1**. 19
Figure S17. UV spectrum of compound **1**... 19
Figure S18. IR spectrum of compound **1**.. 20
Figure S19. ^{1}H NMR spectrum of compound **2** (500 MHz, D_2O).. 21
Figure S20. 1D-selective TOCSY spectrum of compound **2** (500 MHz, D_2O, excitation at δ 5.21, H-A1α). 22
Figure S21. 1D-selective TOCSY spectrum of compound **2** (500 MHz, D_2O, excitation at δ 4.63, H-A1β).. 23
Figure S22. 1D-selective TOCSY spectrum of compound **2** (500 MHz, D_2O, excitation at δ 5.38, H-**B**1, **C**1, and **D**1). 24
Figure S23. 1D-selective TOCSY spectrum of compound **2** (500 MHz, D_2O, excitation at δ 4.44, H-**D**6a)... 25
Figure S24. 1D-selective TOCSY spectrum of compound **2** (500 MHz, D_2O, excitation at δ 5.23, H-**E**1).. 26
Figure S25. 1D-selective TOCSY spectrum of compound **2** (500 MHz, D_2O, excitation at δ 5.87, H-**F**1).. 27
Figure S26. ^{13}C NMR spectrum of compound **2** (125 MHz, D_2O).. 28
Figure S27. DEPT-135 spectrum of compound **2** (125 MHz, D_2O)...................................... 29
Figure S28. HSQC spectrum of compound **2** (500 MHz, D_2O). 30
Figure S29. ^{1}H-^{1}H COSY spectrum of compound **2** (500 MHz, D_2O)................................. 31
Figure S30. 2D-TCOSY spectrum of compound **2** (500 MHz, D_2O)................................... 32

Figure S31. HSQC-TCOSY spectrum of compound **2** (500 MHz, D_2O). 33
Figure S32. HMBC spectrum of compound **2** (500 MHz, D_2O). 34
Figure S33. NOESY spectrum of compound **2** (500 MHz, D_2O). 35
Figure S34. HRESIMS spectrum of compound **2**. 36
Figure S35. UV spectrum of compound **2**. 36
Figure S36. IR spectrum of compound **2**. 37
Figure S37. ^{1}H NMR spectrum of compound **1** (500 MHz, D_2O). 38
Figure S38. 1D-selective TOCSY spectrum of compound **3** (500 MHz, D_2O, excitation at δ 5.26, H-**A**1α). 39
Figure S39. 1D-selective TOCSY spectrum of compound **3** (500 MHz, D_2O, excitation at δ 4.66, H-**A**1β). 40
Figure S40. 1D-selective TOCSY spectrum of compound **3** (500 MHz, D_2O, excitation at δ 5.43, H-**B**1, H-**C**1, H-**D**1). 41
Figure S41. 1D-selective TOCSY spectrum of compound **3** (500 MHz, D_2O, excitation at δ 4.46, H-**D**6a). 42
Figure S42. 1D-selective TOCSY spectrum of compound **3** (500 MHz, D_2O, excitation at δ 5.29, H-**E**1). 43
Figure S43. 1D-selective TOCSY spectrum of compound **3** (500 MHz, D_2O, excitation at δ 6.01, H-**F**7). 44
Figure S44. 1D-selective TOCSY spectrum of compound **3** (500 MHz, D_2O, excitation at δ 5.40, H-**G**1). 45
Figure S45. 1D-selective TOCSY spectrum of compound **3** (500 MHz, D_2O, excitation at δ 5.34, H-**H**1). 46
Figure S46. 1D-selective TOCSY spectrum of compound **3** (500 MHz, D_2O, excitation at δ 5.93, H-**I**7). 47
Figure S47. ^{13}C NMR spectrum of compound **3** (125 MHz, D_2O). 48
Figure S48. DEPT-135 spectrum of compound **3** (125 MHz, D_2O). 49
Figure S49. HSQC spectrum of compound **3** (500 MHz, D_2O). 50
Figure S50. ^{1}H-^{1}H COSY spectrum of compound **3** (500 MHz, D_2O). 51
Figure S51. 2D-TOCSY spectrum of compound **3** (500 MHz, D_2O). 52
Figure S52. HSQC-TOCSY spectrum of compound **3** (500 MHz, D_2O). 53
Figure S53. HMBC spectrum of compound **3** (500 MHz, D_2O). 54
Figure S54. NOESY spectrum of compound **3** (500 MHz, D_2O). 55
Figure S55. HRESIMS spectrum of compound **3**. 56
Figure S56. UV spectrum of compound **3**. 56
Figure S57. IR spectrum of compound **3**. 57
Figure S58. ^{1}H NMR spectrum of the common basic hydrolysis product (**9**) of compounds **1** and **5** (500 MHz, D_2O). 58
Figure S59. ^{1}H NMR spectrum of the common basic hydrolysis product (**10**) of compound **3** (500 MHz, D_2O). 59

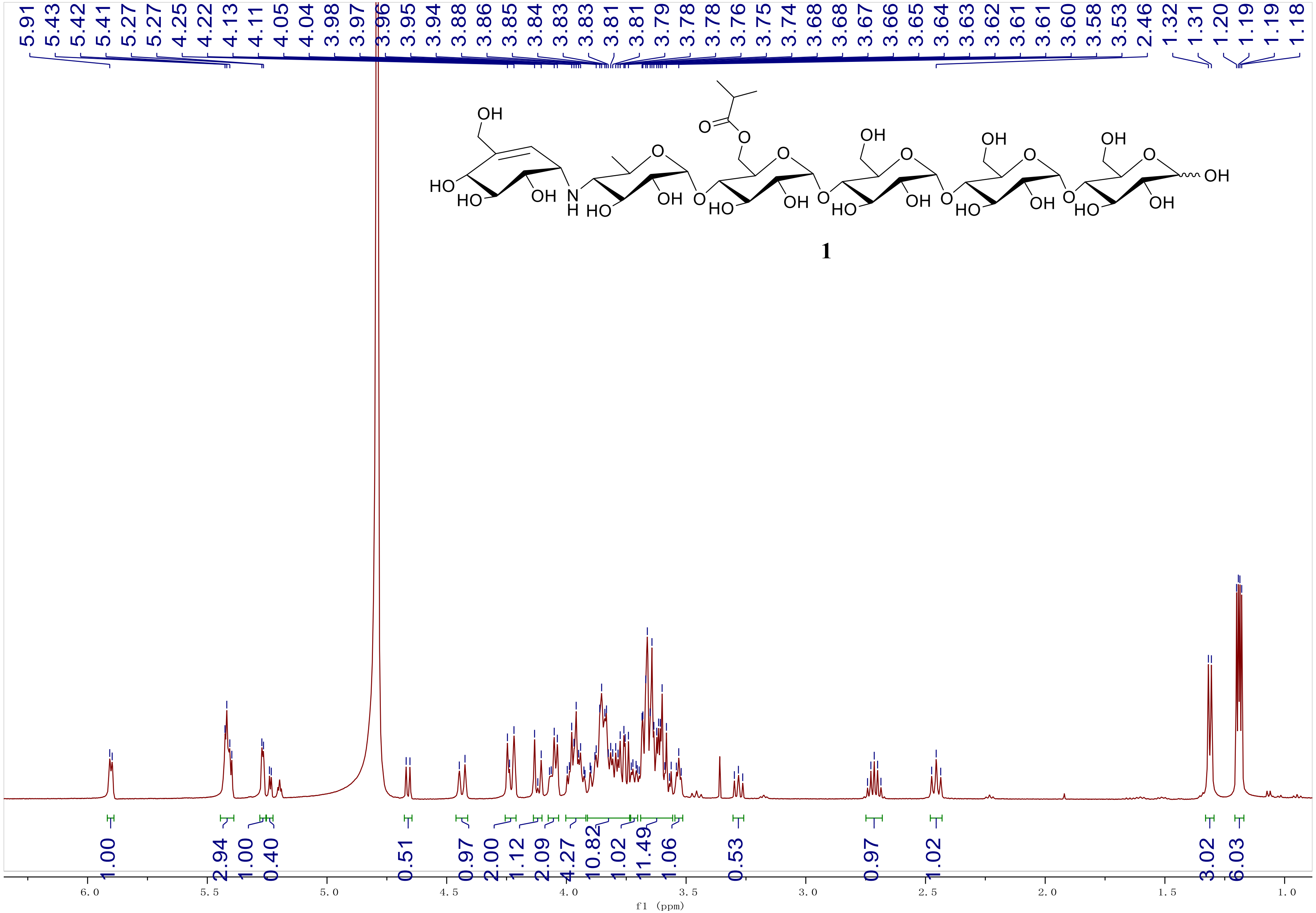

Figure S1. ^{1}H NMR spectrum of compound **1** (500 MHz, D_2O).

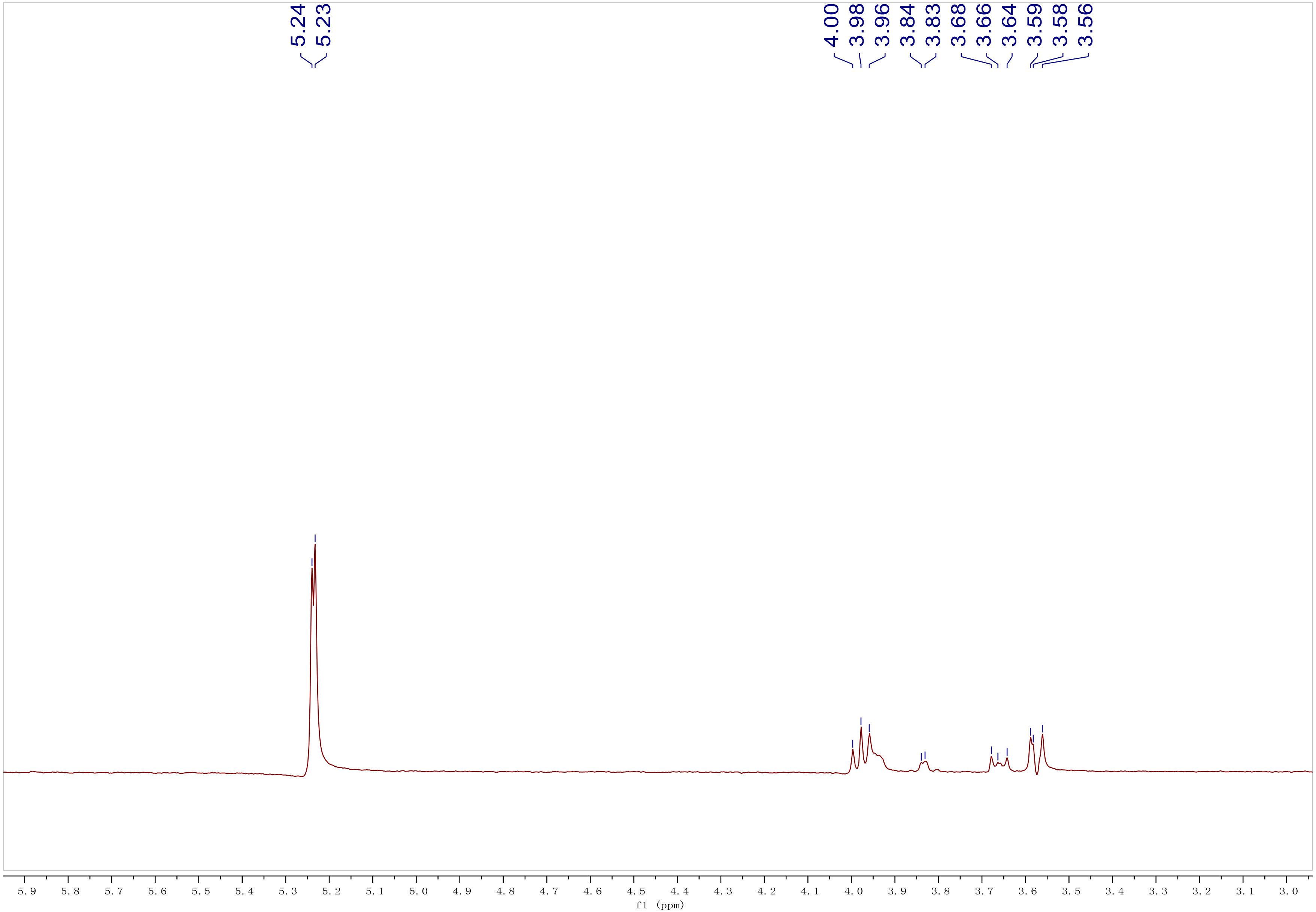

Figure S2. 1D-selective TOCSY spectrum of compound **1** (500 MHz, D_2O, excitation at δ 5.24, H-**A**1α).

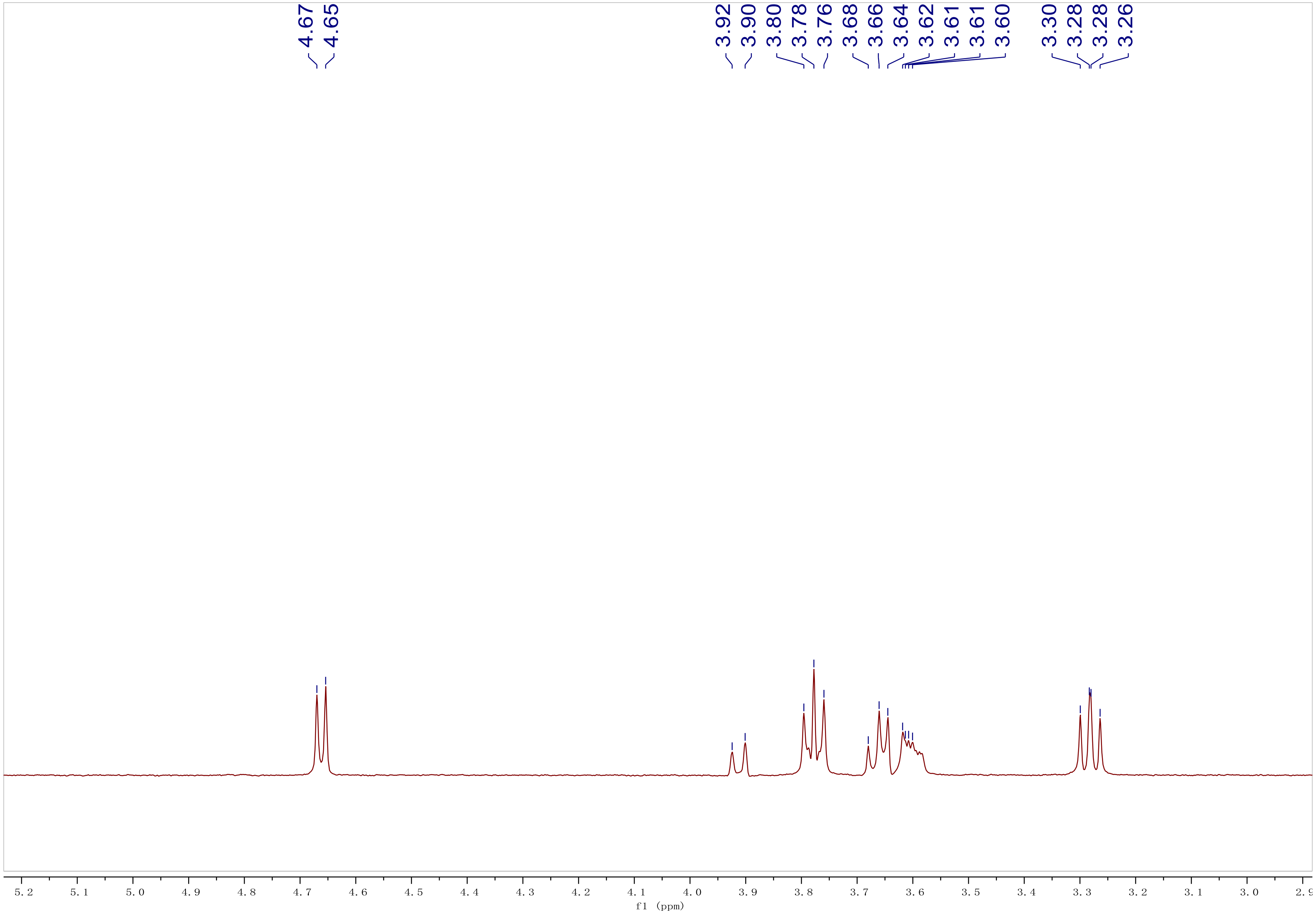

Figure S3. 1D-selective TOCSY spectrum of compound **2** (500 MHz, D_2O, excitation at δ 4.66, H-**A**1β).

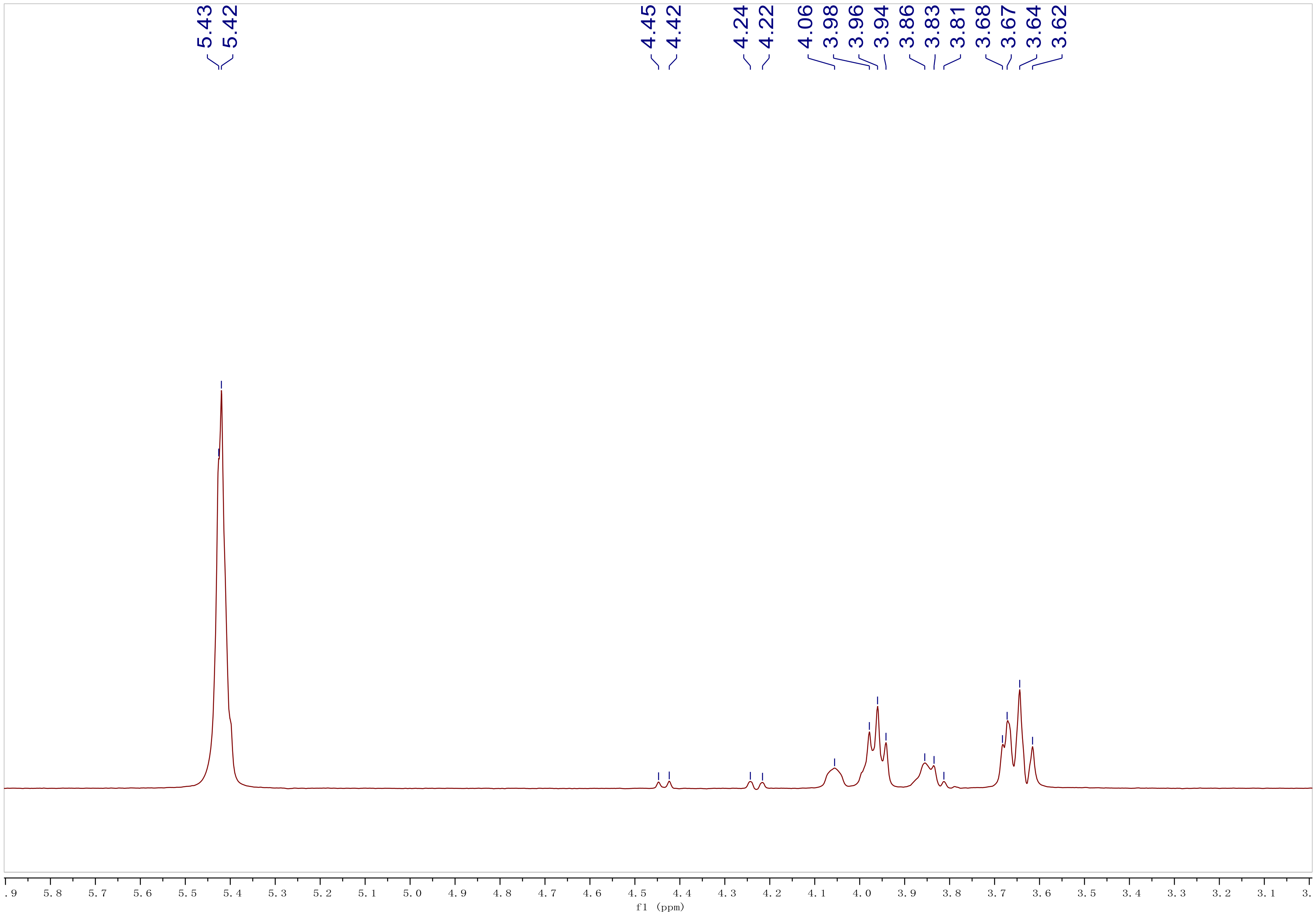

Figure S4. 1D-selective TOCSY spectrum of compound **1** (500 MHz, D_2O, excitation at δ 5.42, H-**B**1, H-**C**1, and H-**D**1).

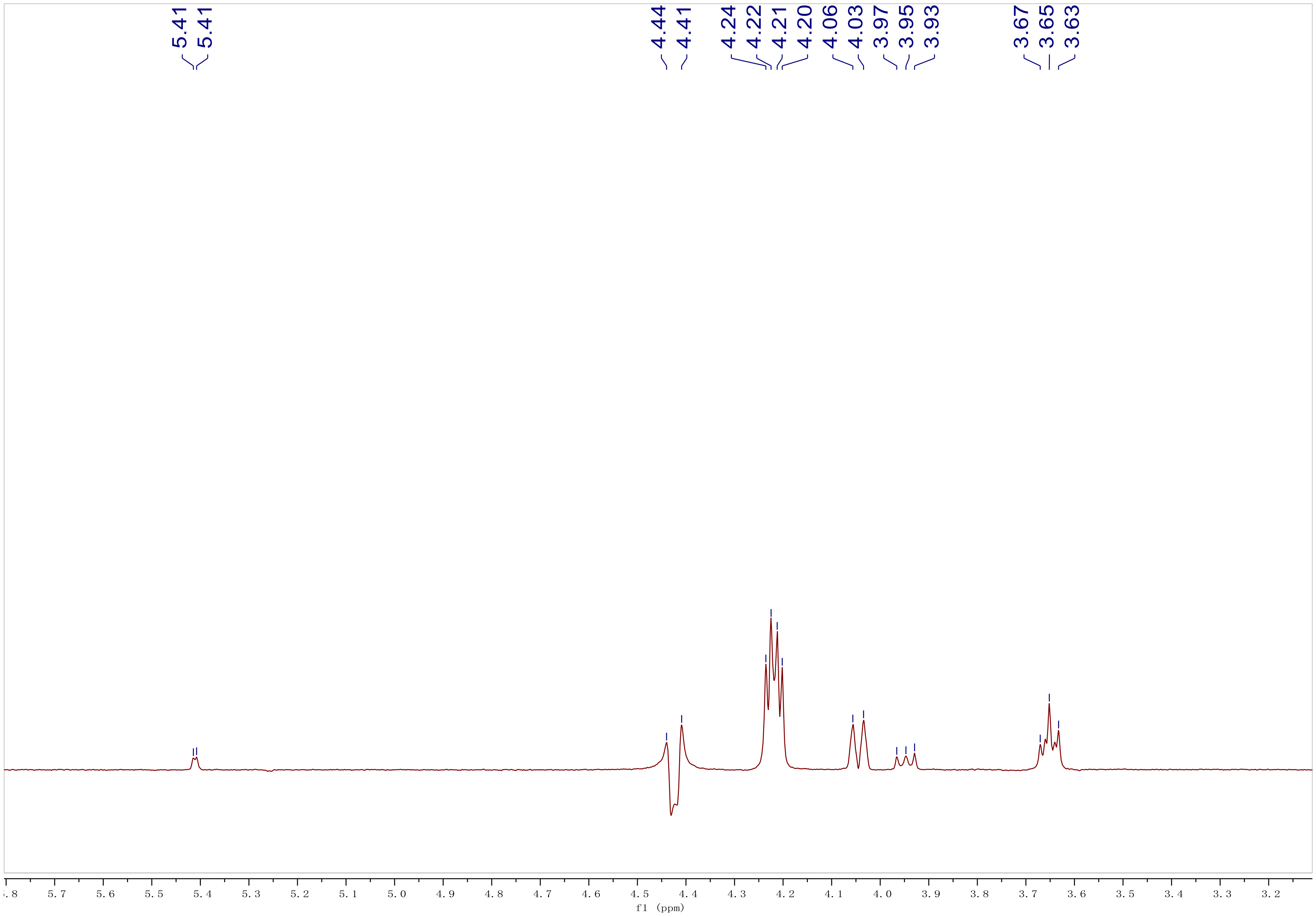

Figure S5. 1D-selective TOCSY spectrum of compound **1** (500 MHz, D_2O, excitation at δ 4.44, H-**D**6a).

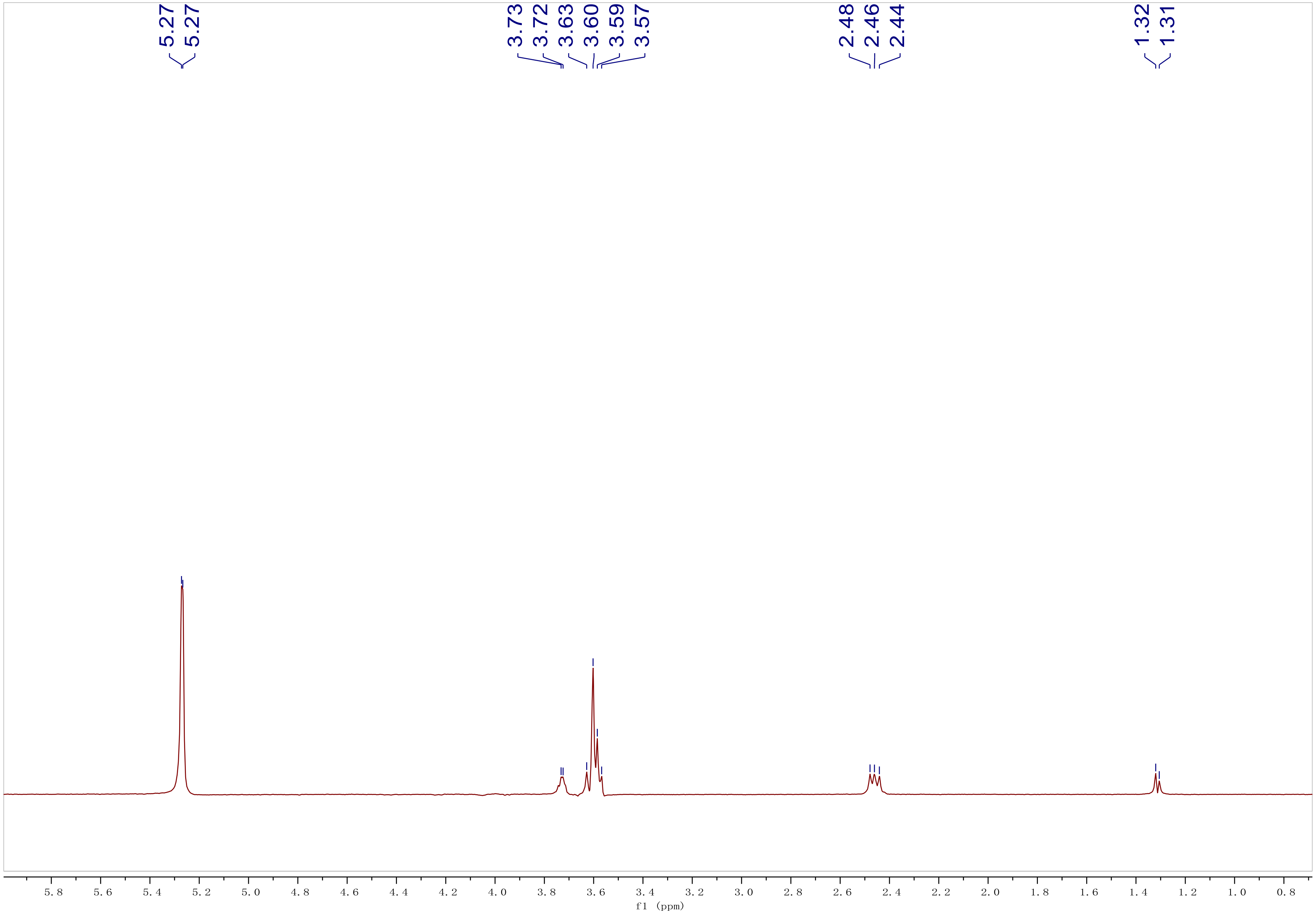

Figure S6. 1D-selective TOCSY spectrum of compound **1** (500 MHz, D_2O, excitation at δ 5.27, H-**E**1).

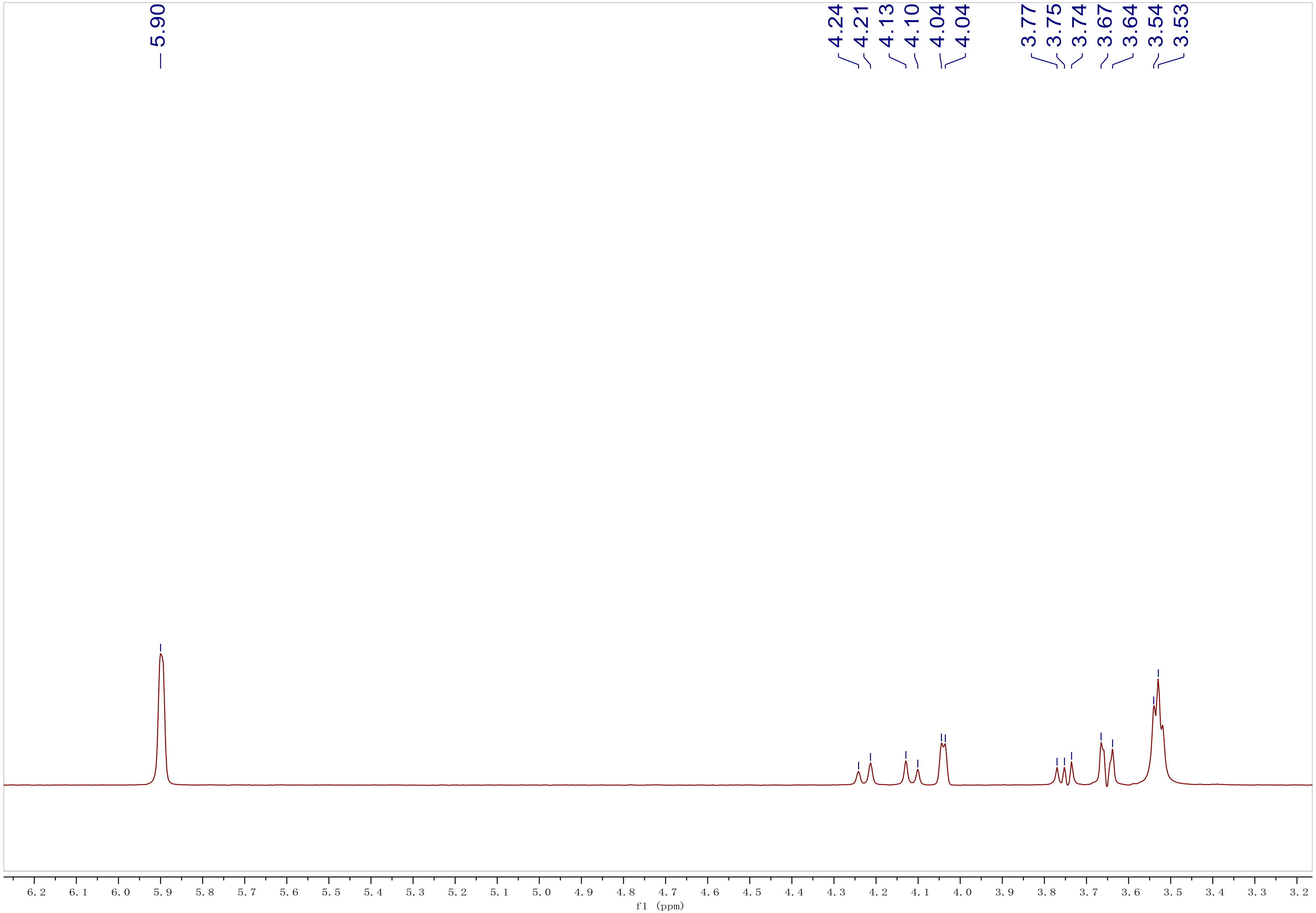

Figure S7. 1D-selective TOCSY spectrum of compound **1** (500 MHz, D_2O, excitation at δ 5.90, H-**F**7).

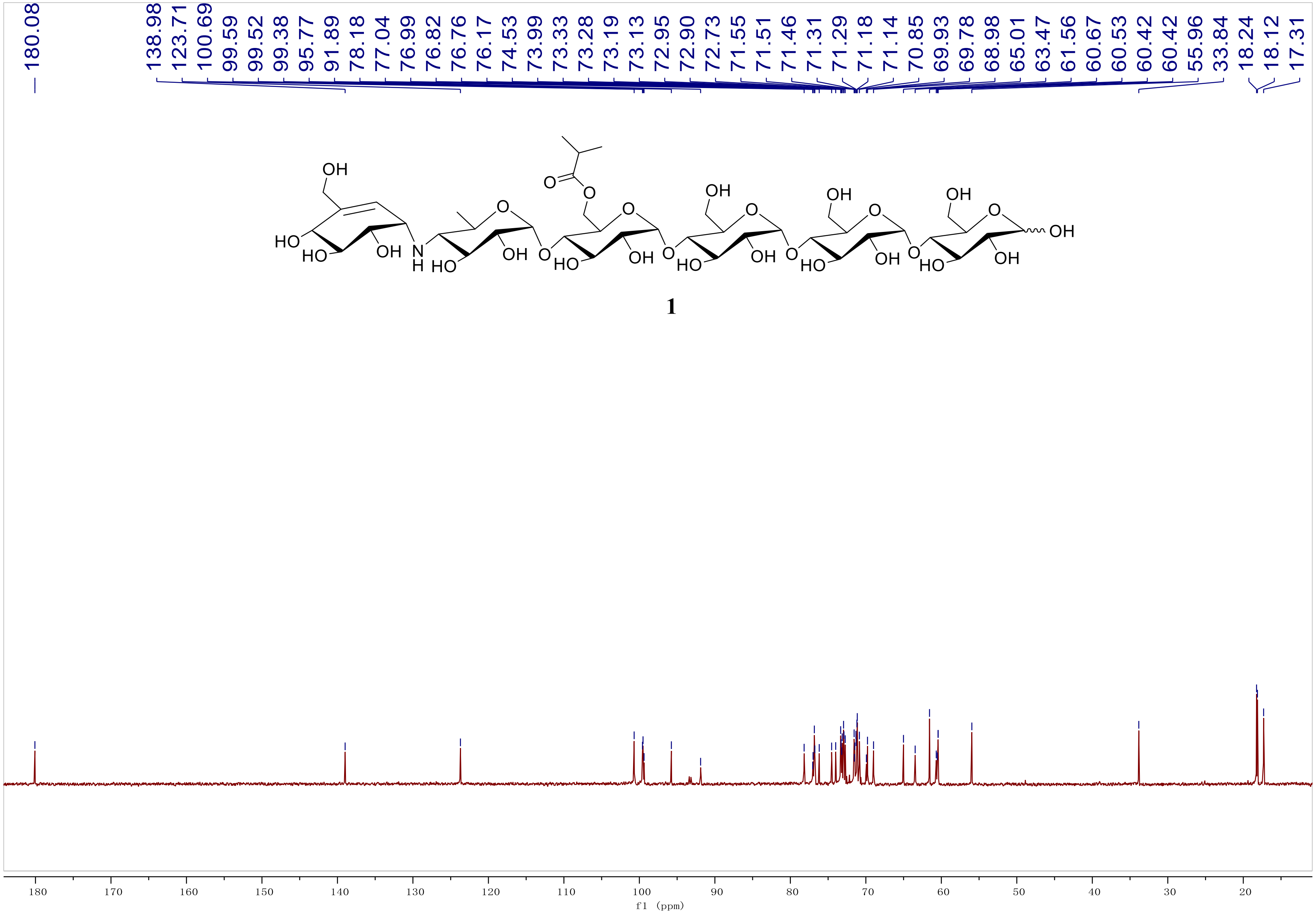

Figure S8. ^{13}C NMR spectrum of compound **1** (125 MHz, D_2O).

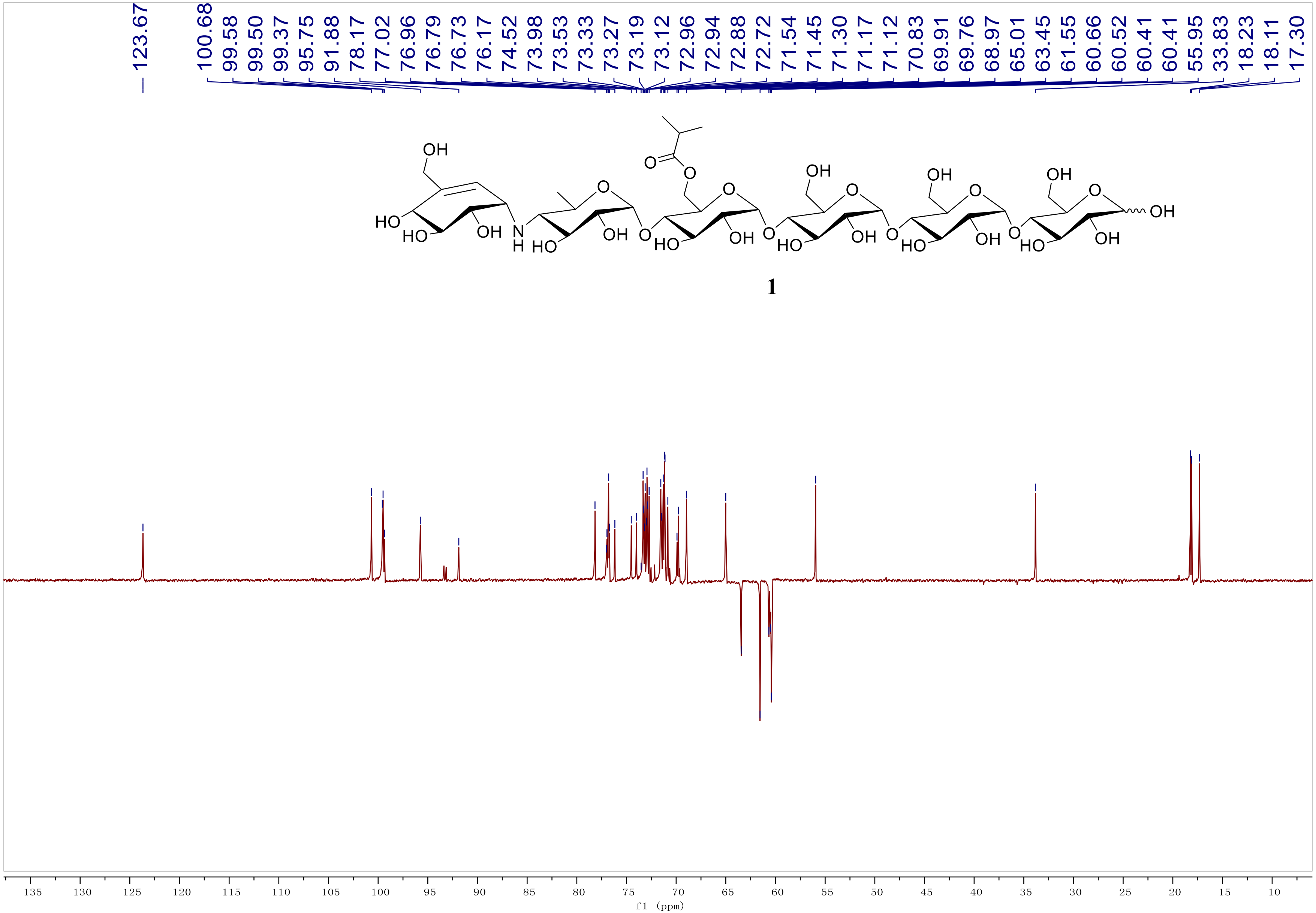

Figure S9. DEPT-135 spectrum of compound **1** (125 MHz, D_2O).

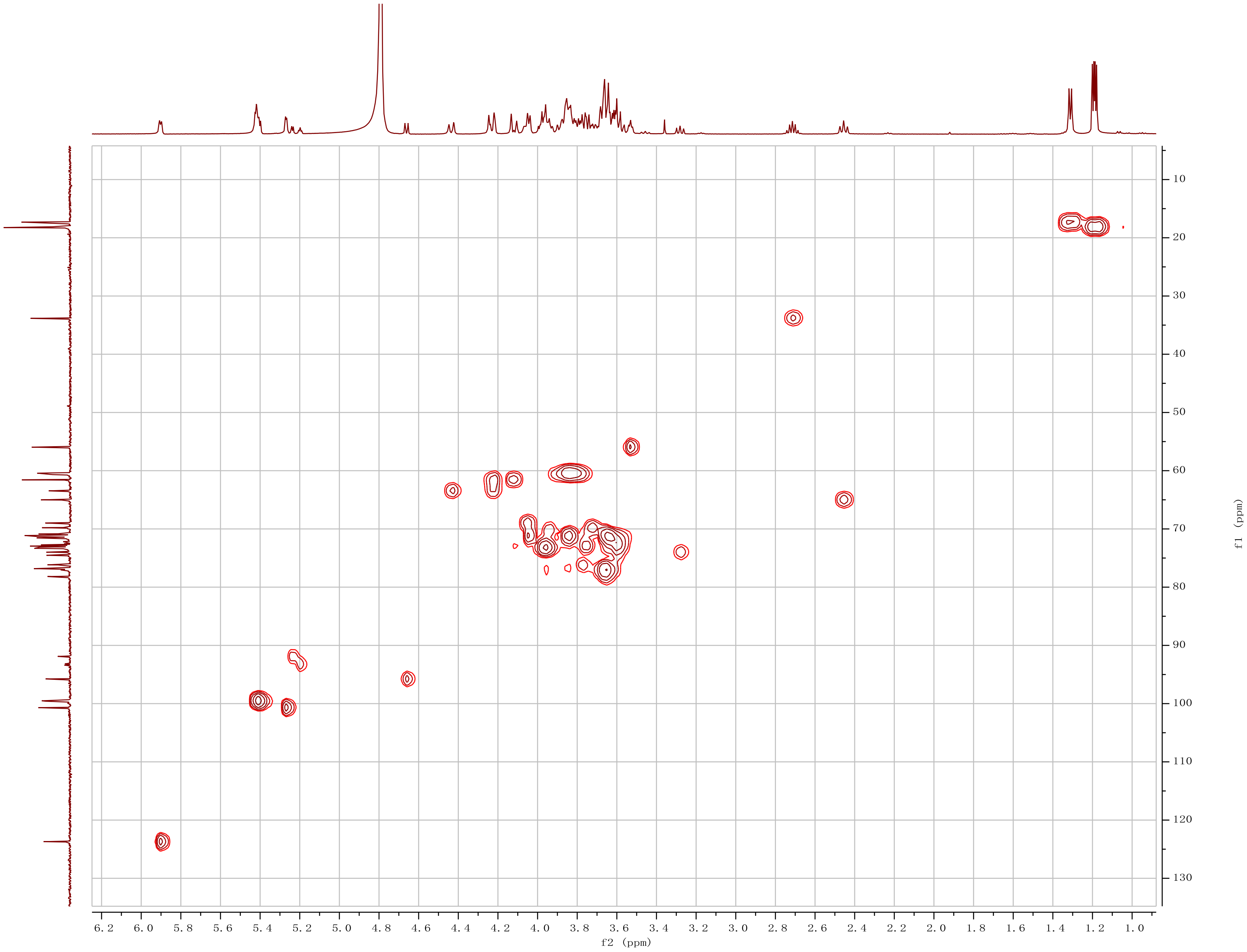

Figure S10. HSQC spectrum of compound **1** (500 MHz, D_2O).

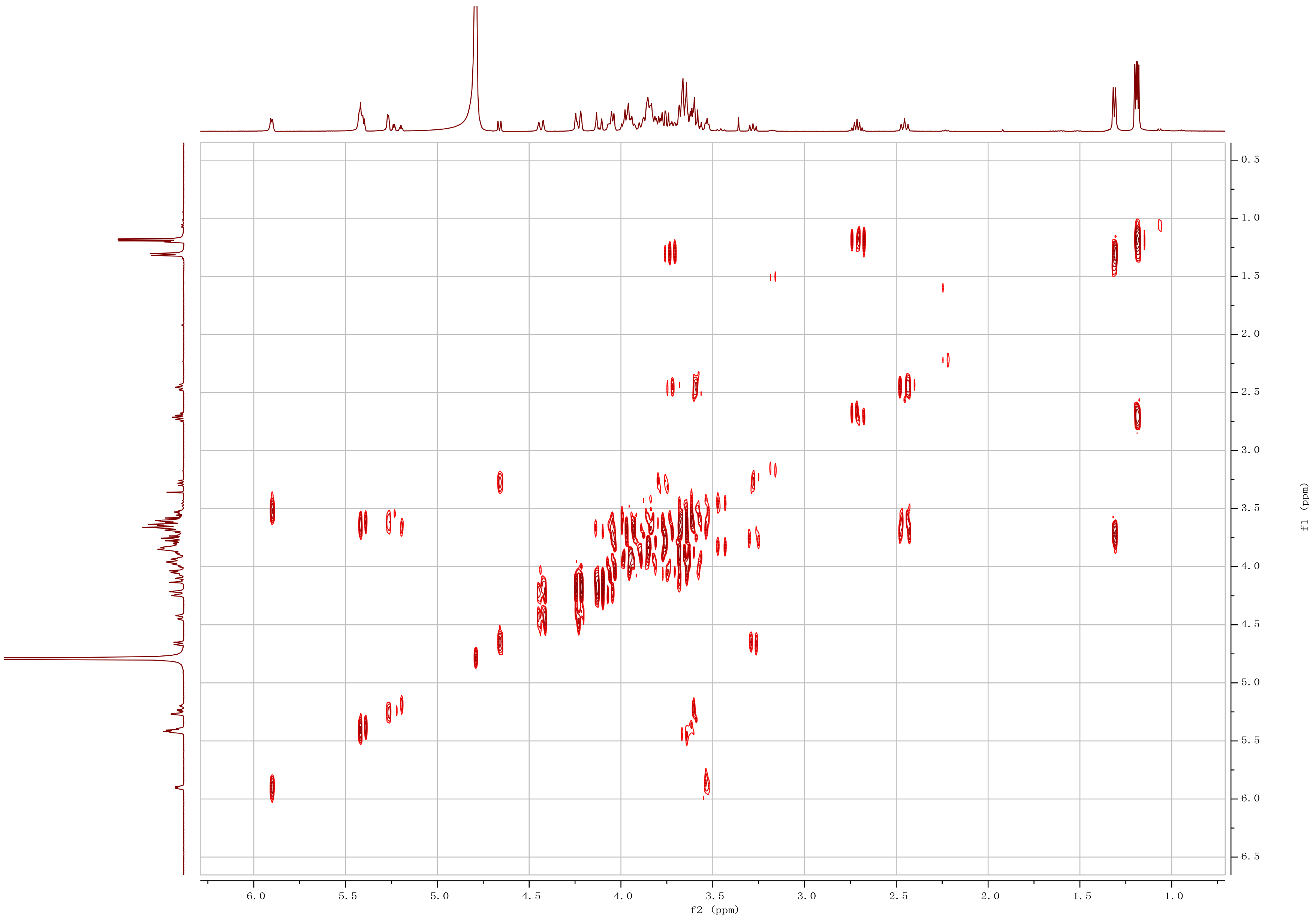

Figure S11. ^{1}H-^{1}H COSY spectrum of compound **1** (500 MHz, D_2O).

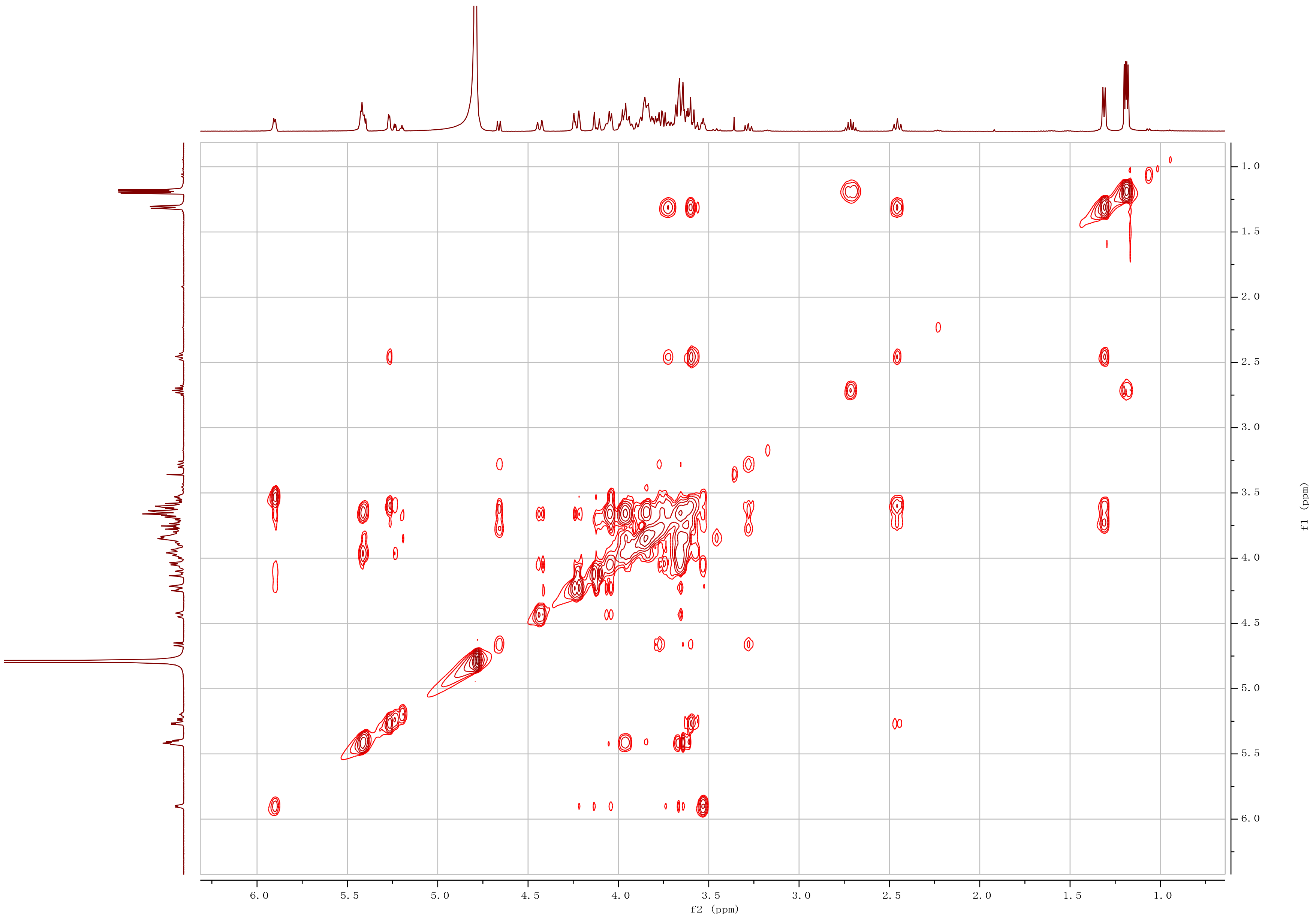

Figure S12. 2D-TCOSY spectrum of compound **1** (500 MHz, D_2O).

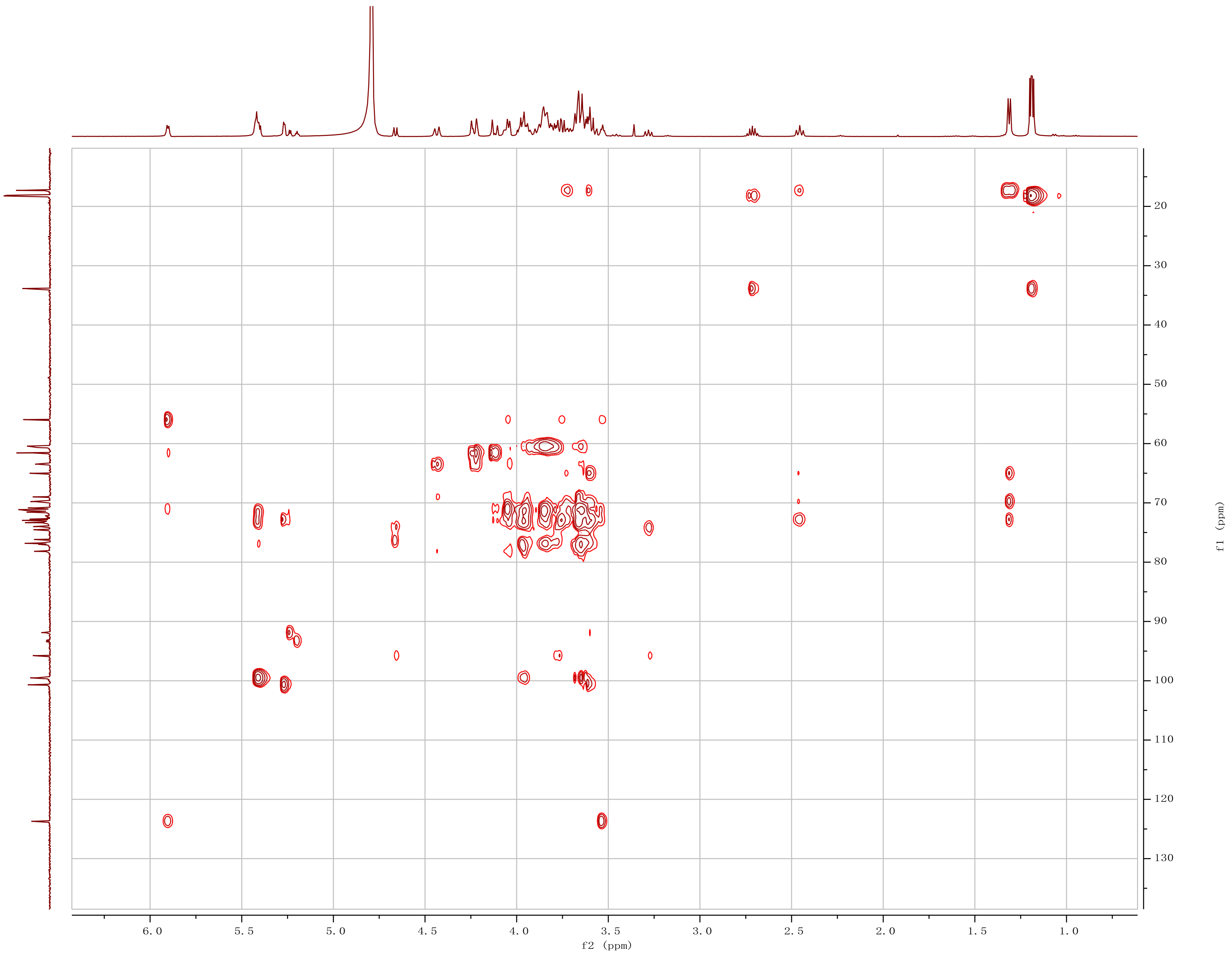

Figure S13. HSQC-TCOSY spectrum of compound **1** (500 MHz, D_2O).

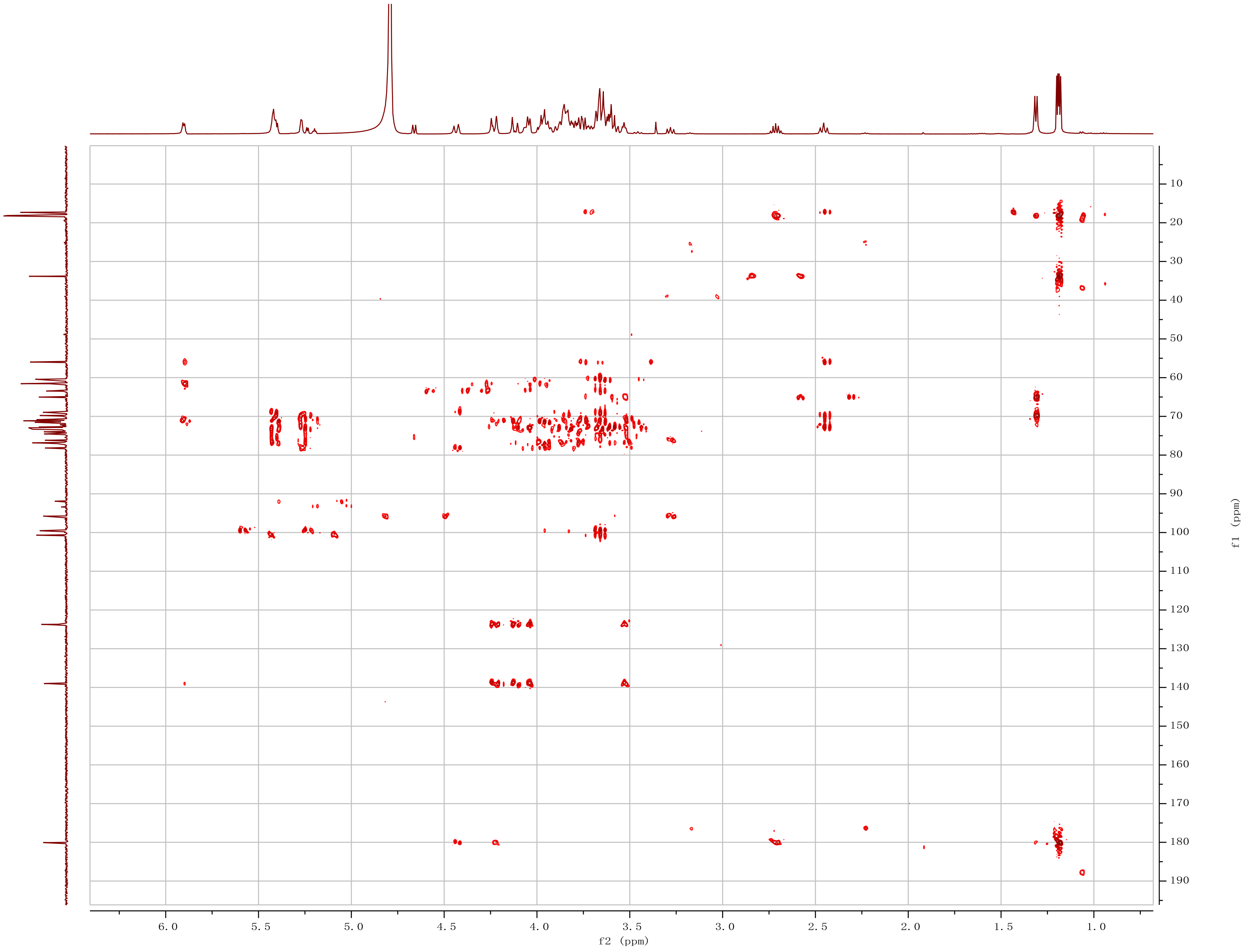

Figure S14. HMBC spectrum of compound **1** (500 MHz, D_2O).

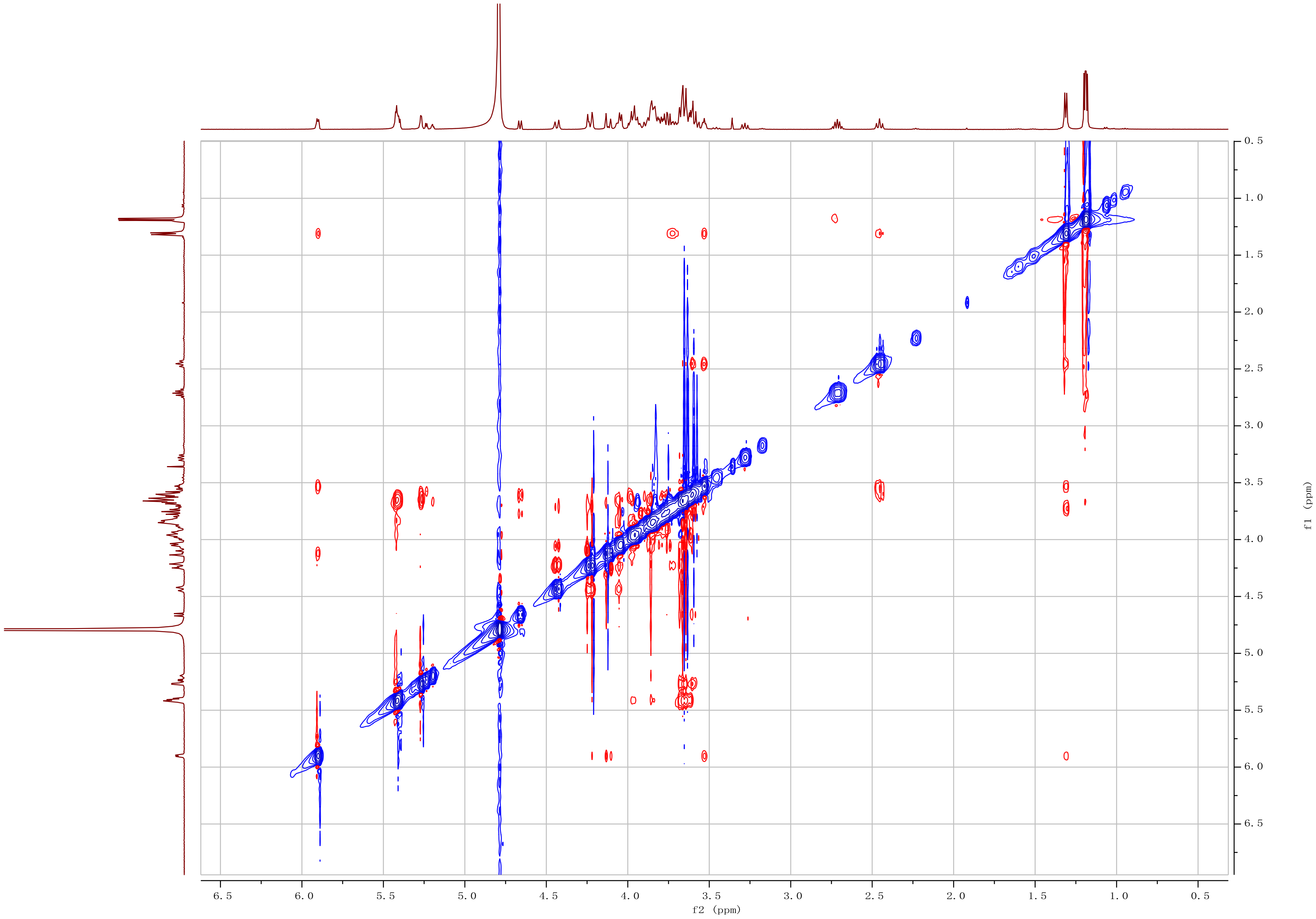

Figure S15. NOESY spectrum of compound **1** (500 MHz, D_2O).

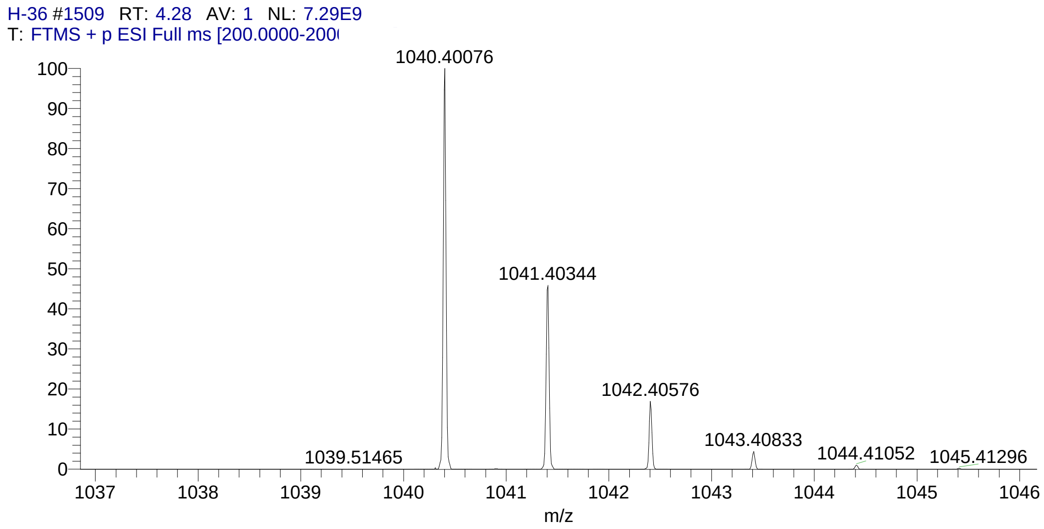

Figure S16. HRESIMS spectrum of compound **1**.

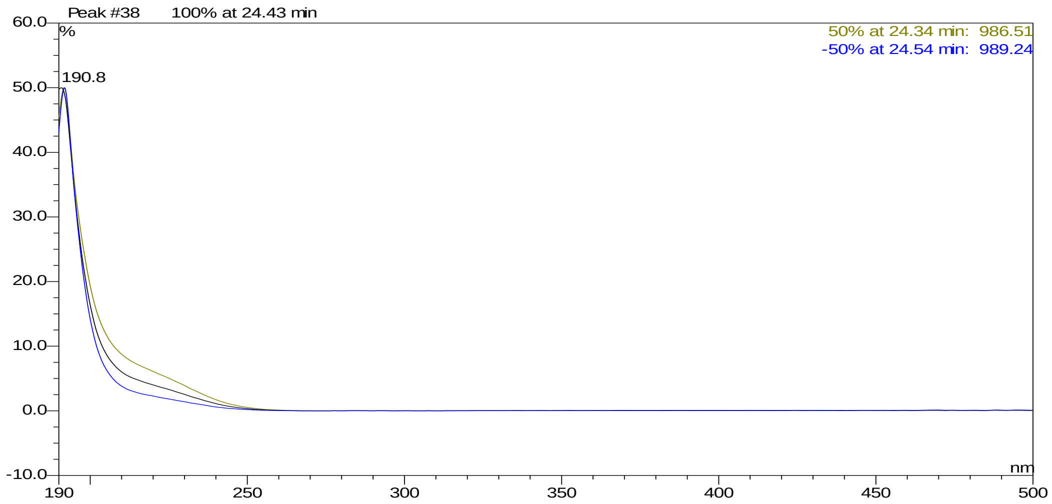

Figure S17. UV spectrum of compound **1**.

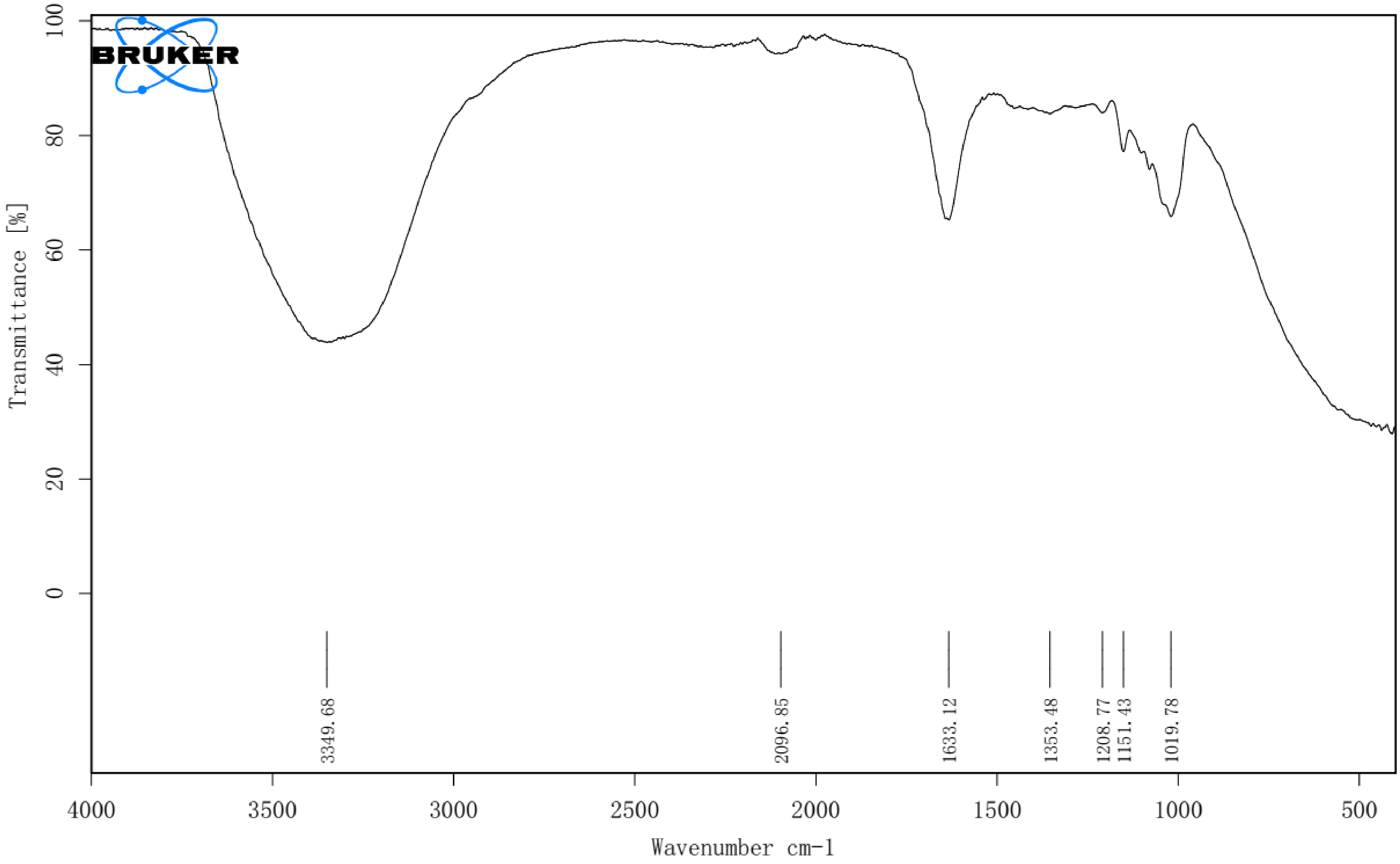

Figure S18. IR spectrum of compound **1**.

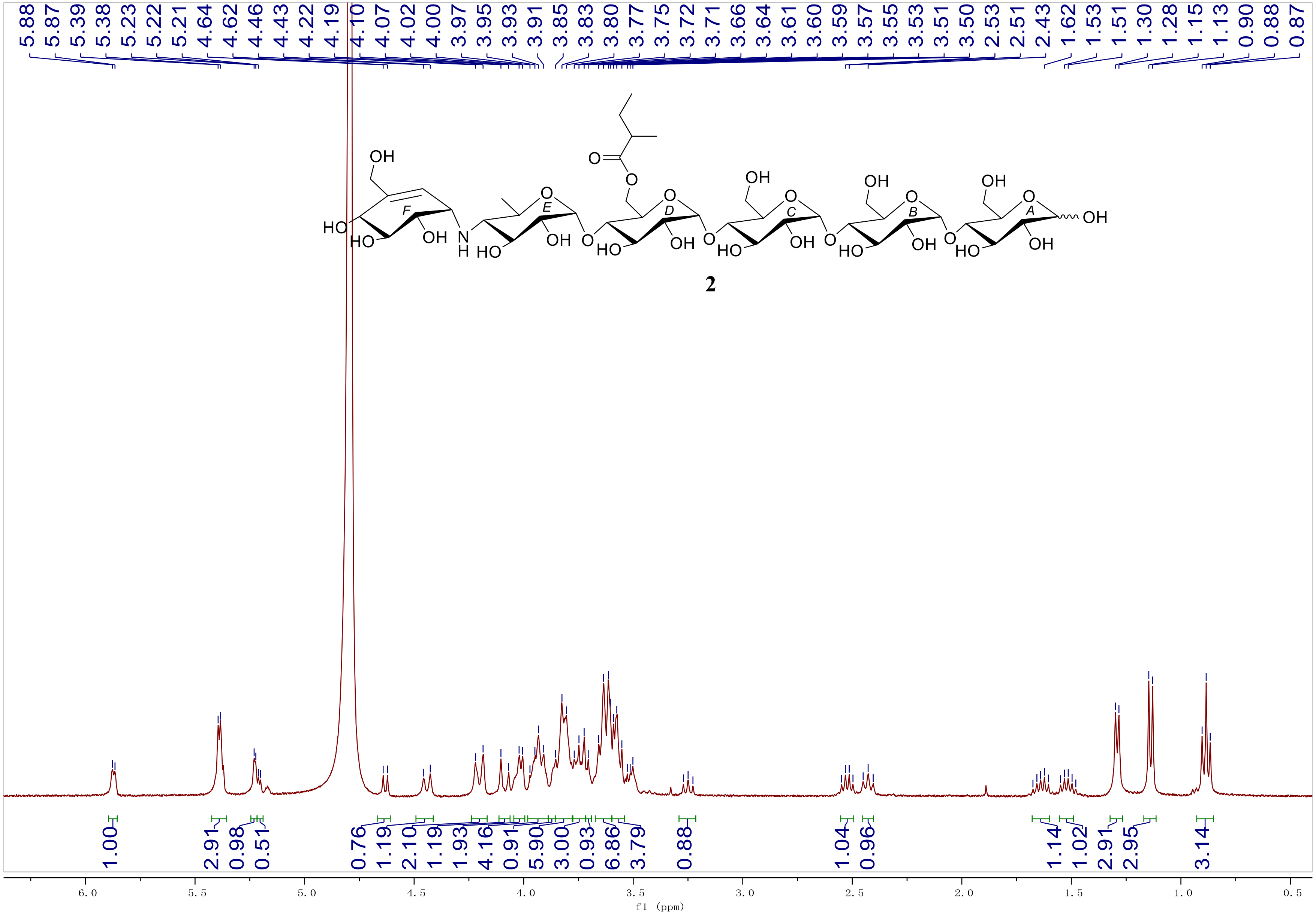

Figure S19. ^{1}H NMR spectrum of compound **2** (500 MHz, D_2O).

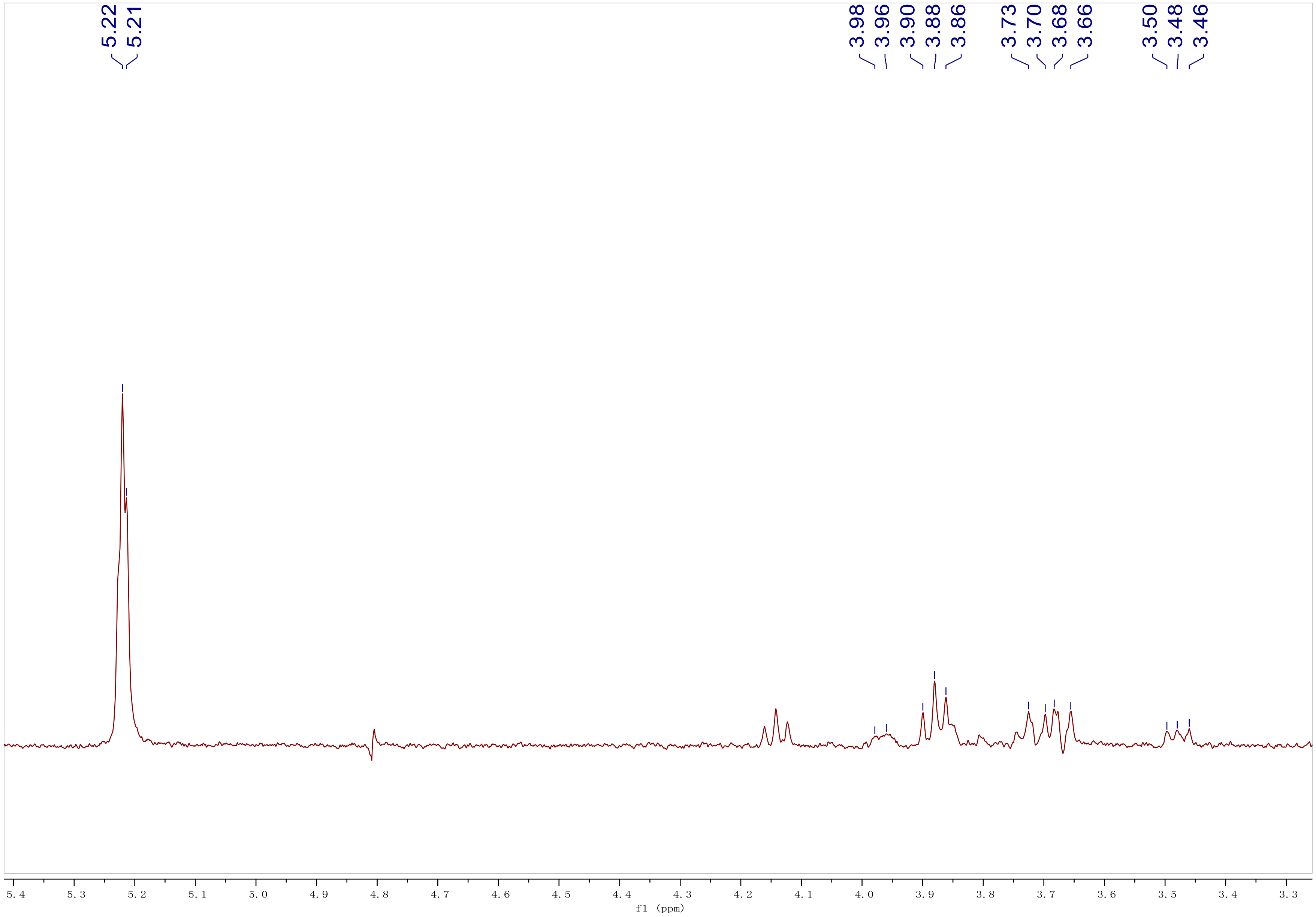

Figure S20. 1D-selective TOCSY spectrum of compound **2** (500 MHz, D_2O, excitation at δ 5.21, H-A1α).

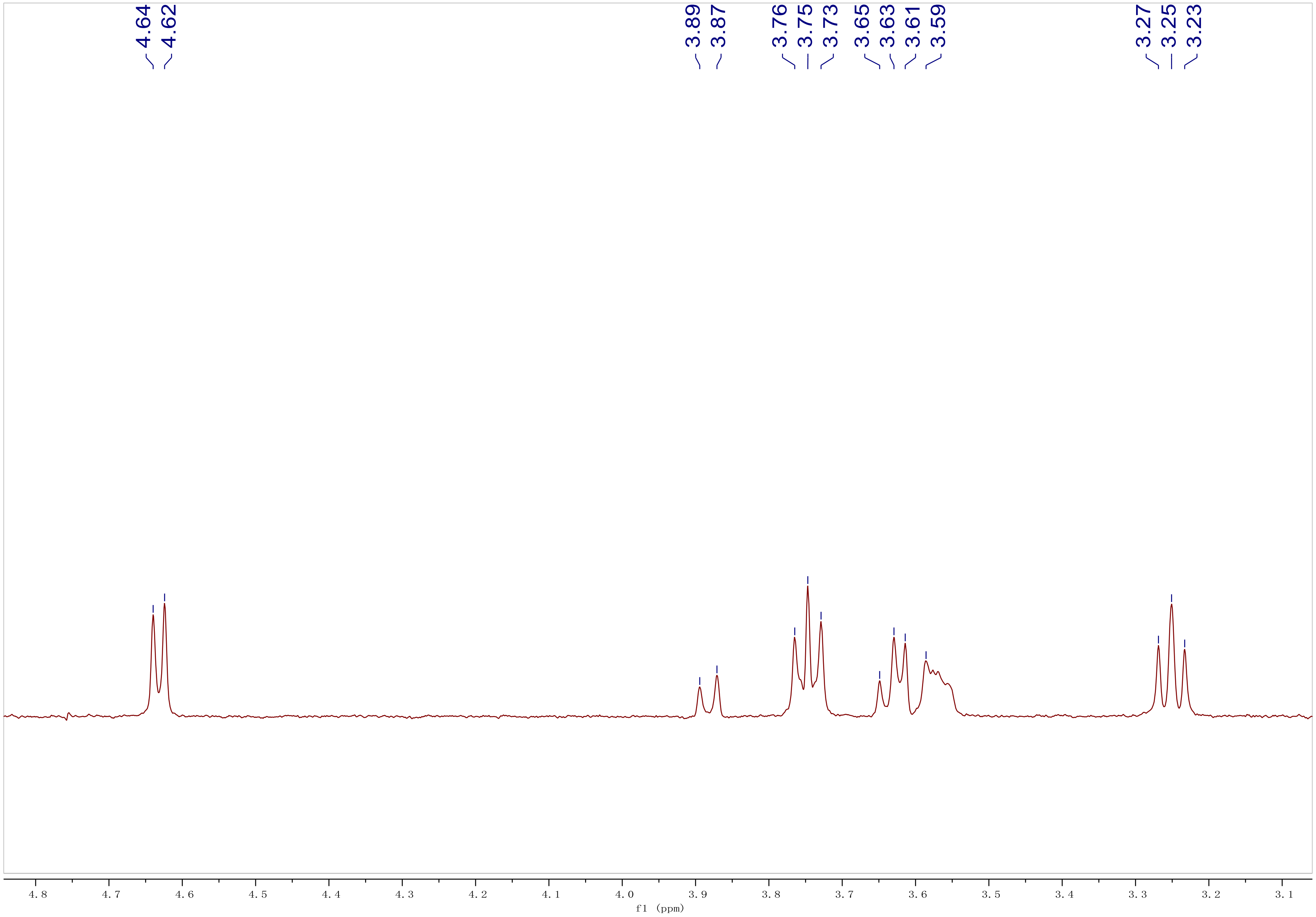

Figure S21. 1D-selective TOCSY spectrum of compound **2** (500 MHz, D_2O, excitation at δ 4.63, H-A1β).

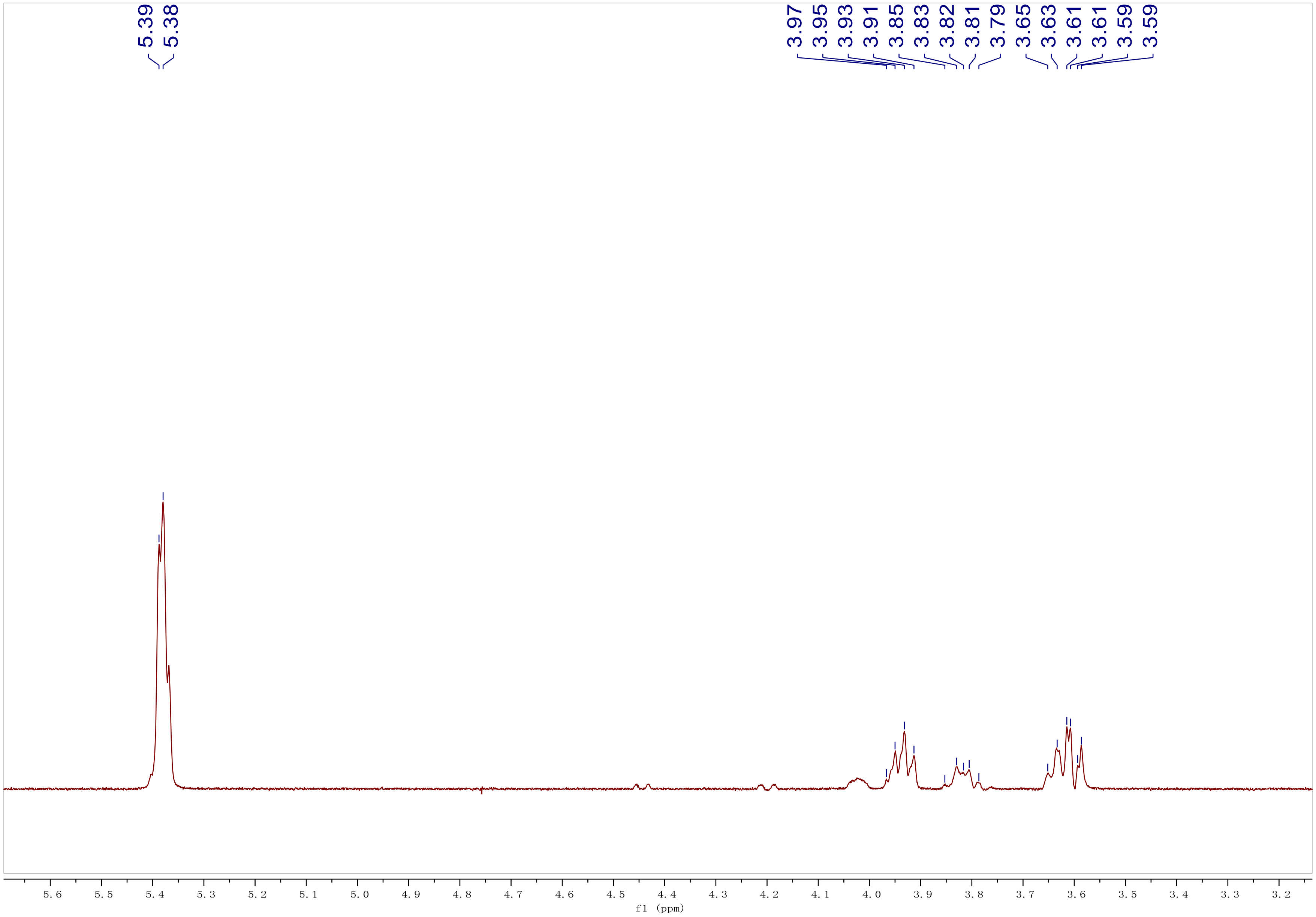

Figure S22. 1D-selective TOCSY spectrum of compound **2** (500 MHz, D_2O, excitation at δ 5.38, H-**B**1, **C**1, and **D**1).

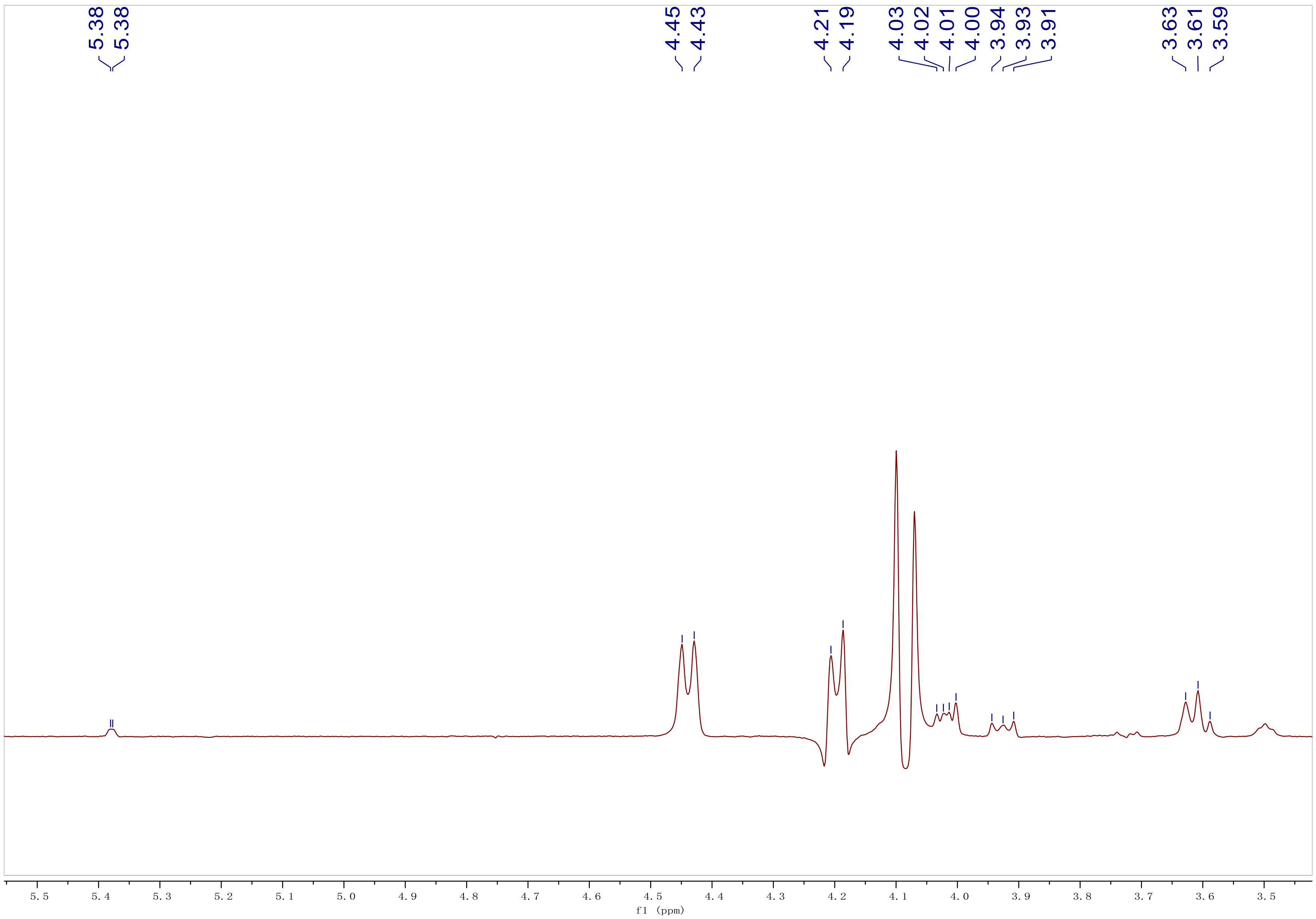

Figure S23. 1D-selective TOCSY spectrum of compound **2** (500 MHz, D_2O, excitation at δ 4.44, H-**D**6a).

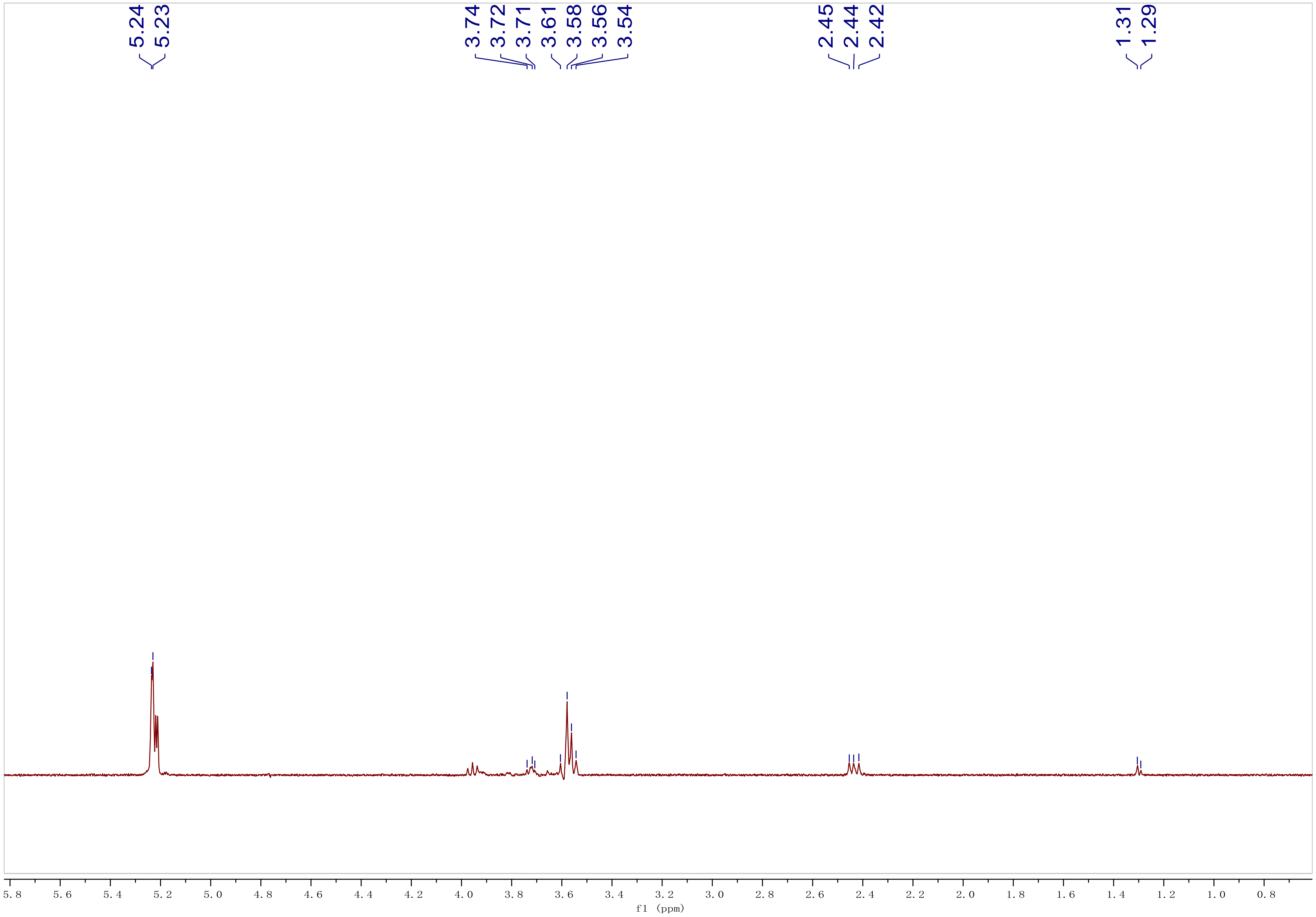

Figure S24. 1D-selective TOCSY spectrum of compound **2** (500 MHz, D_2O, excitation at δ 5.23, H-**E**1).

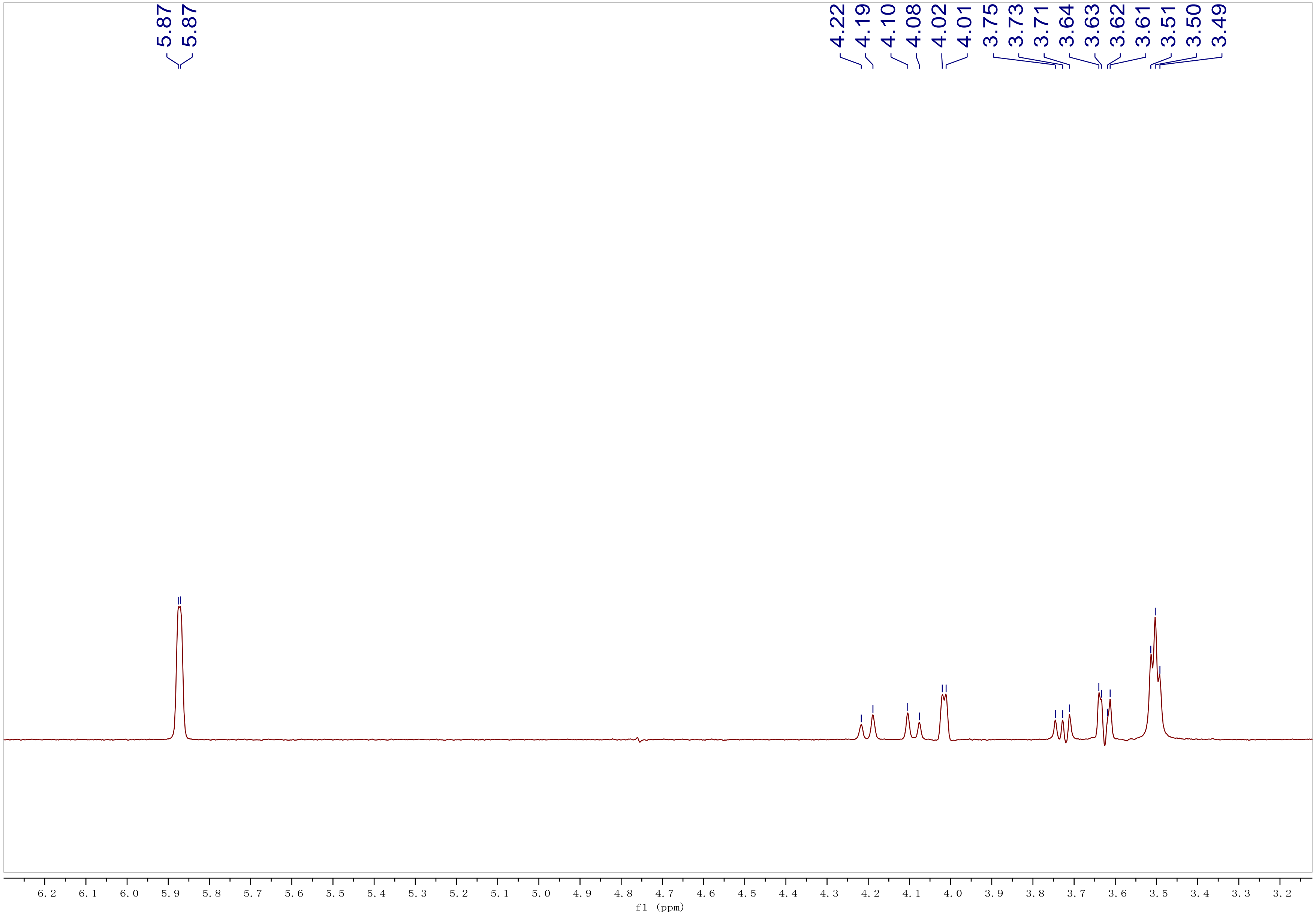

Figure S25. 1D-selective TOCSY spectrum of compound **2** (500 MHz, D_2O, excitation at δ 5.87, H-**F**1).

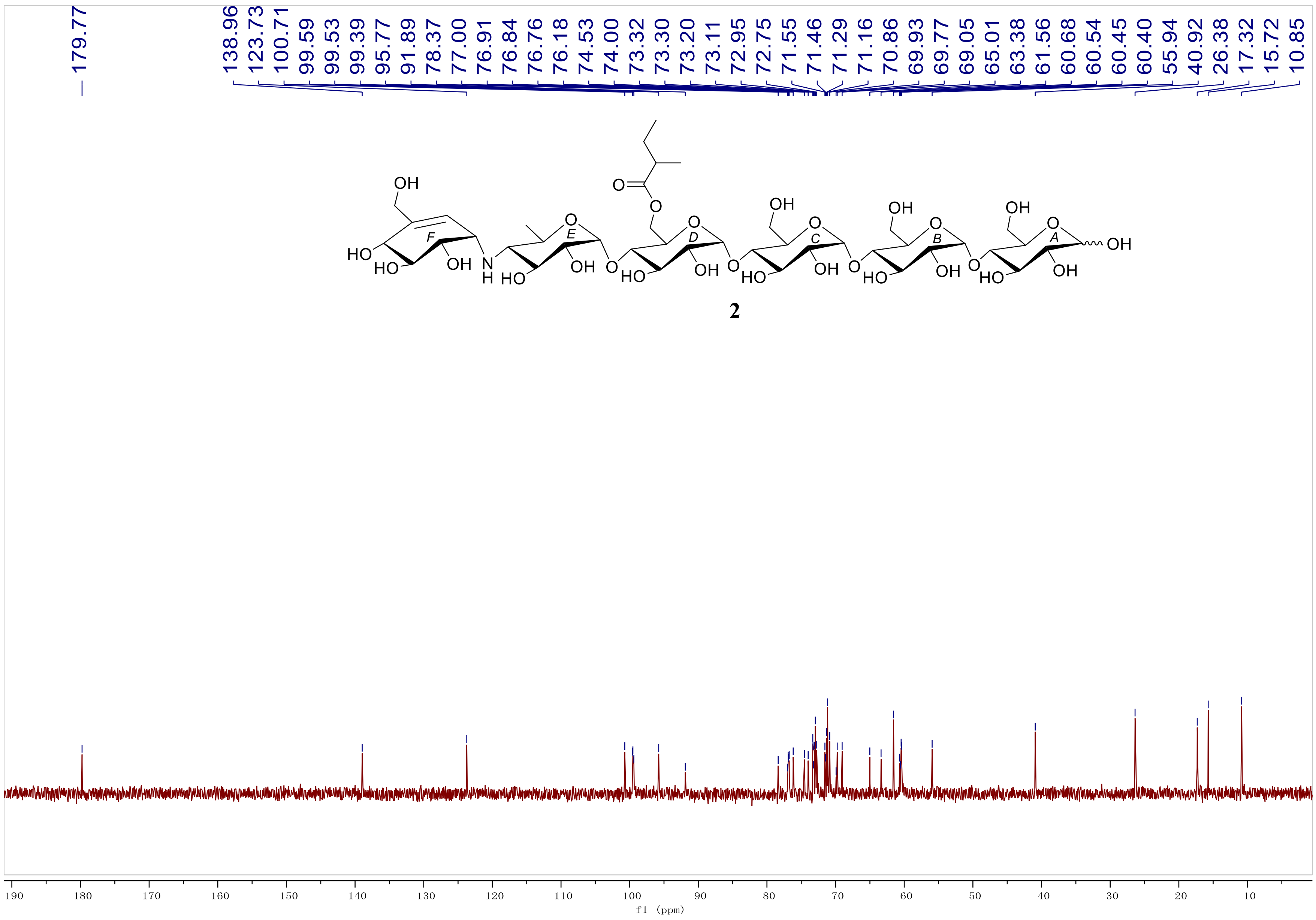

Figure S26. ^{13}C NMR spectrum of compound **2** (125 MHz, D_2O).

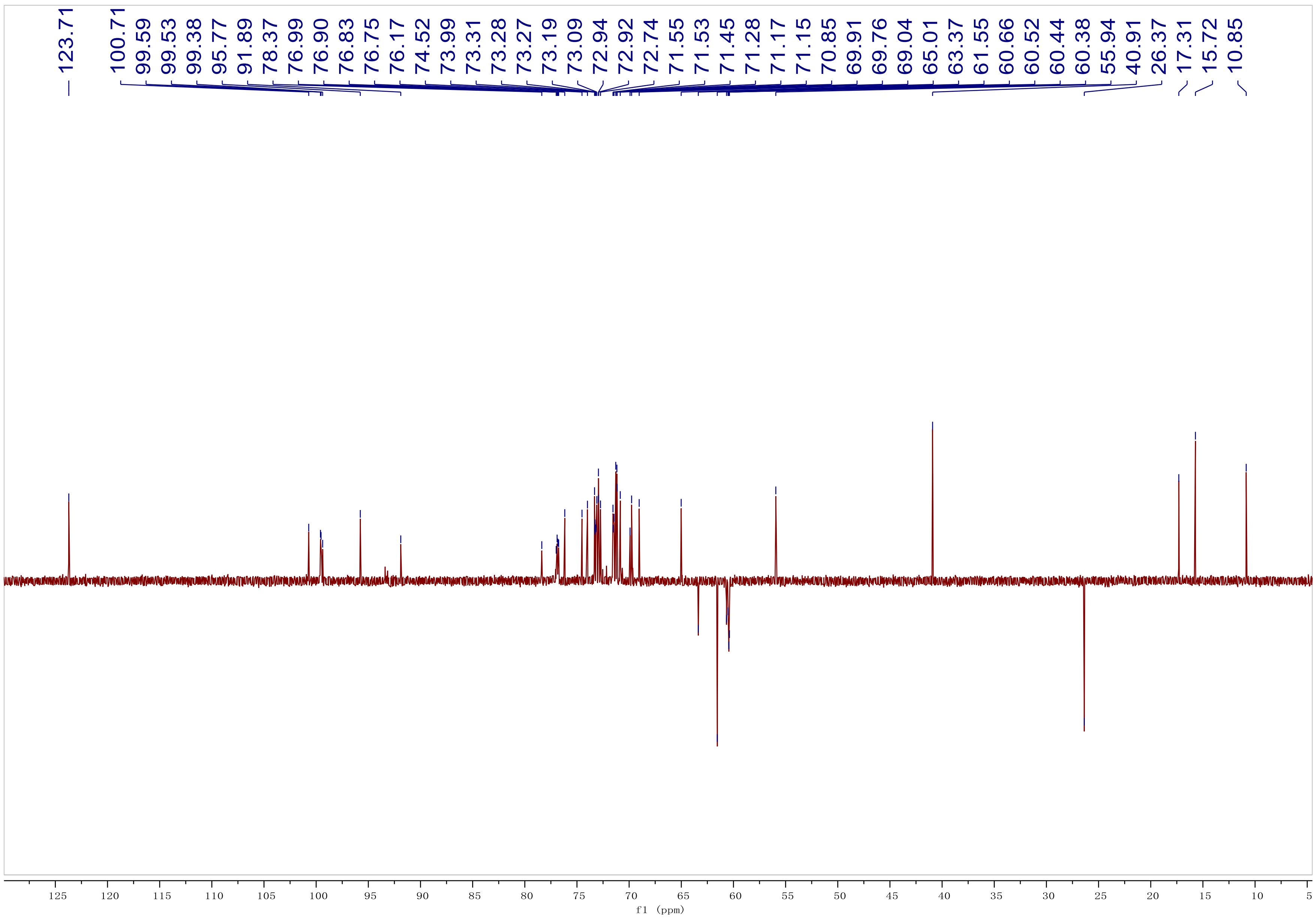

Figure S27. DEPT-135 spectrum of compound **2** (125 MHz, D_2O).

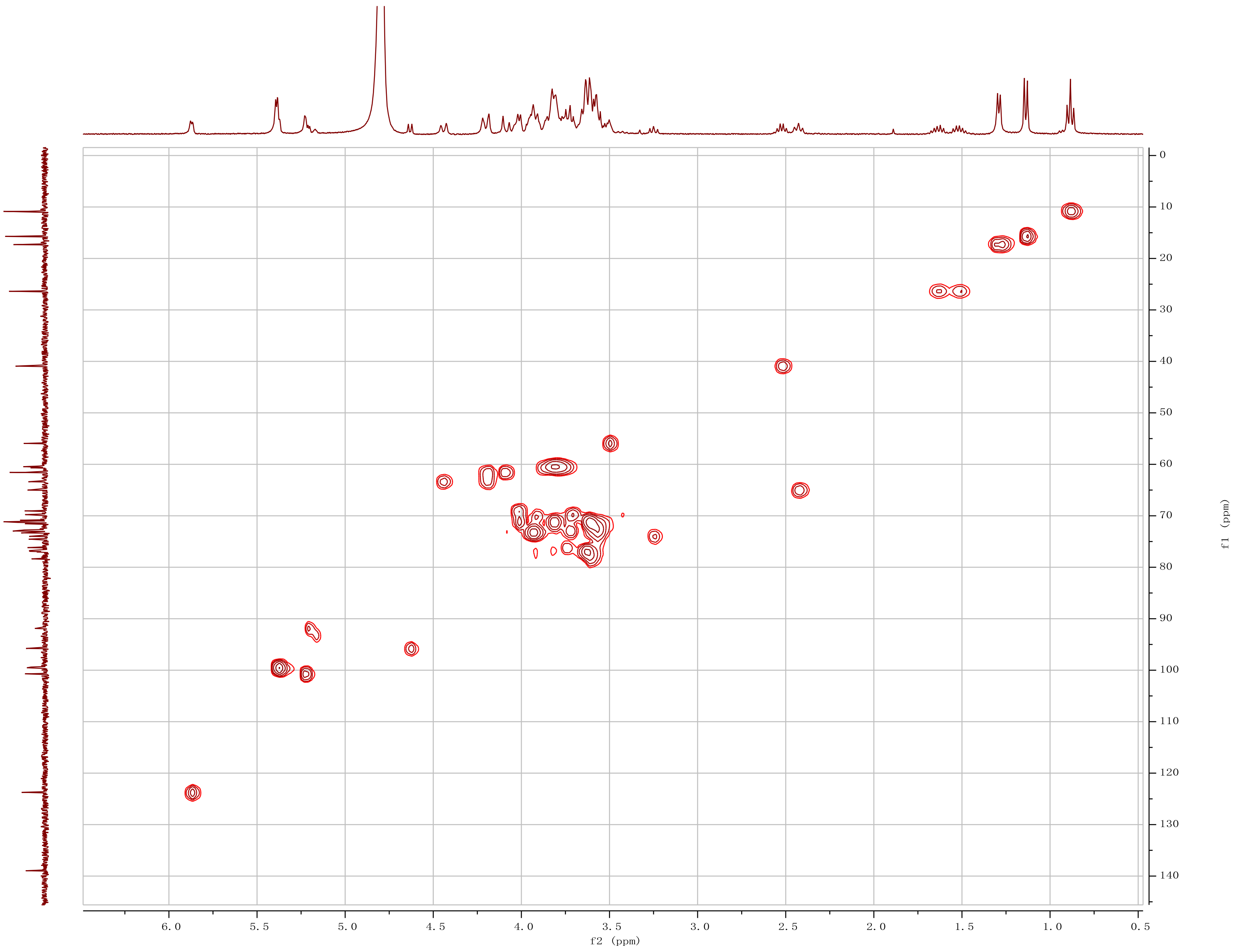

Figure S28. HSQC spectrum of compound **2** (500 MHz, D_2O).

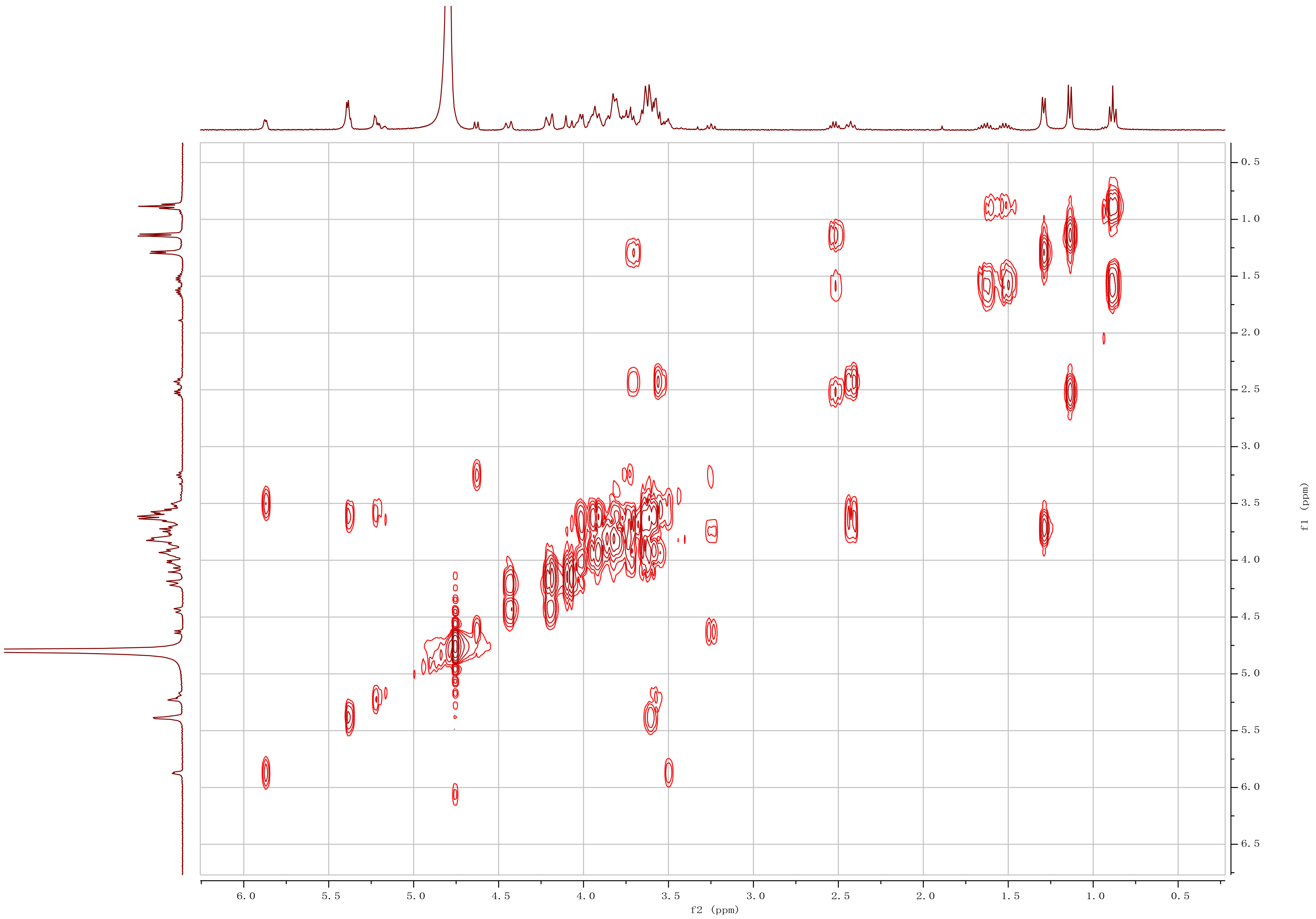

Figure S29. ^{1}H-^{1}H COSY spectrum of compound **2** (500 MHz, D_2O).

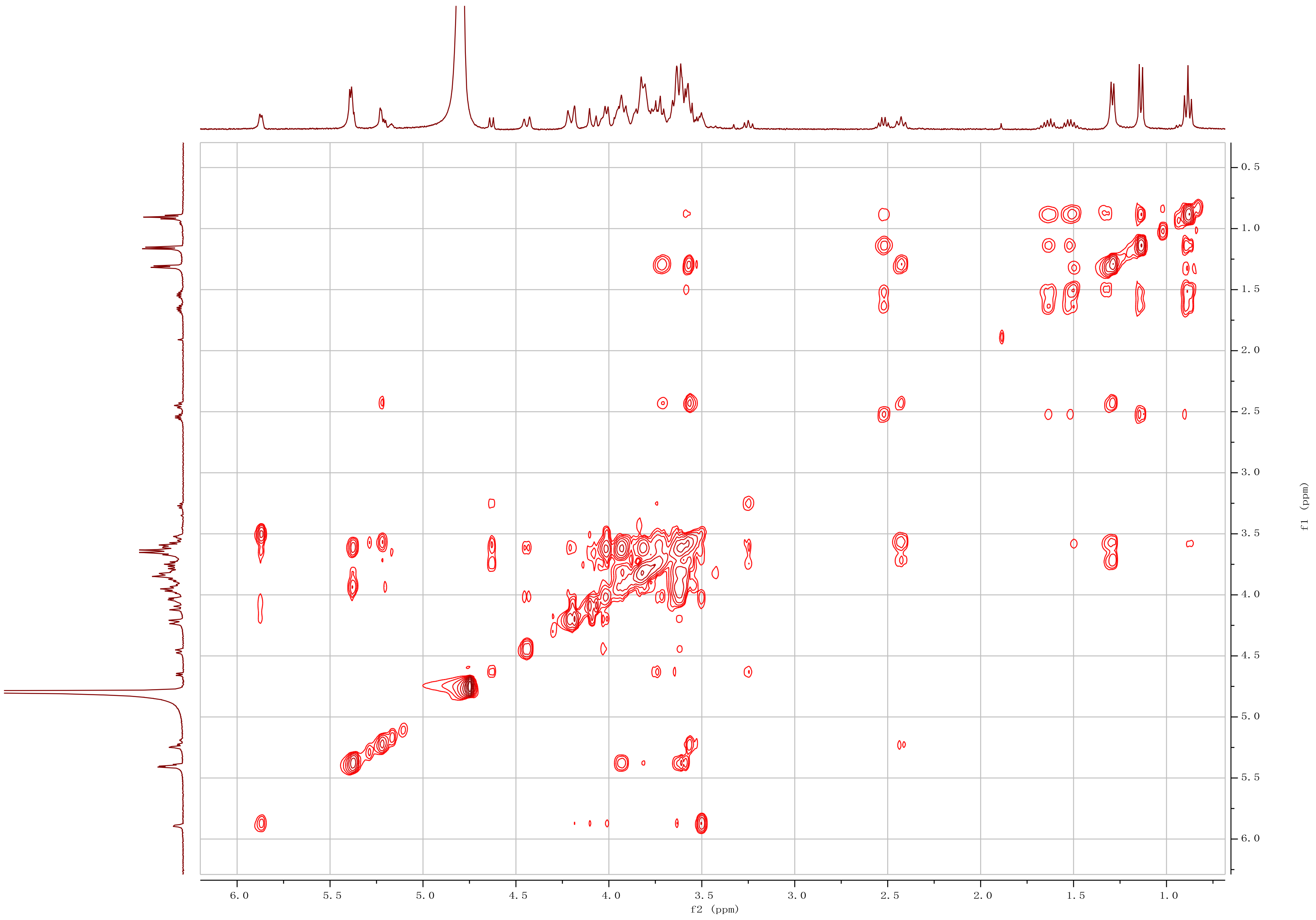

Figure S30. 2D-TCOSY spectrum of compound **2** (500 MHz, D_2O).

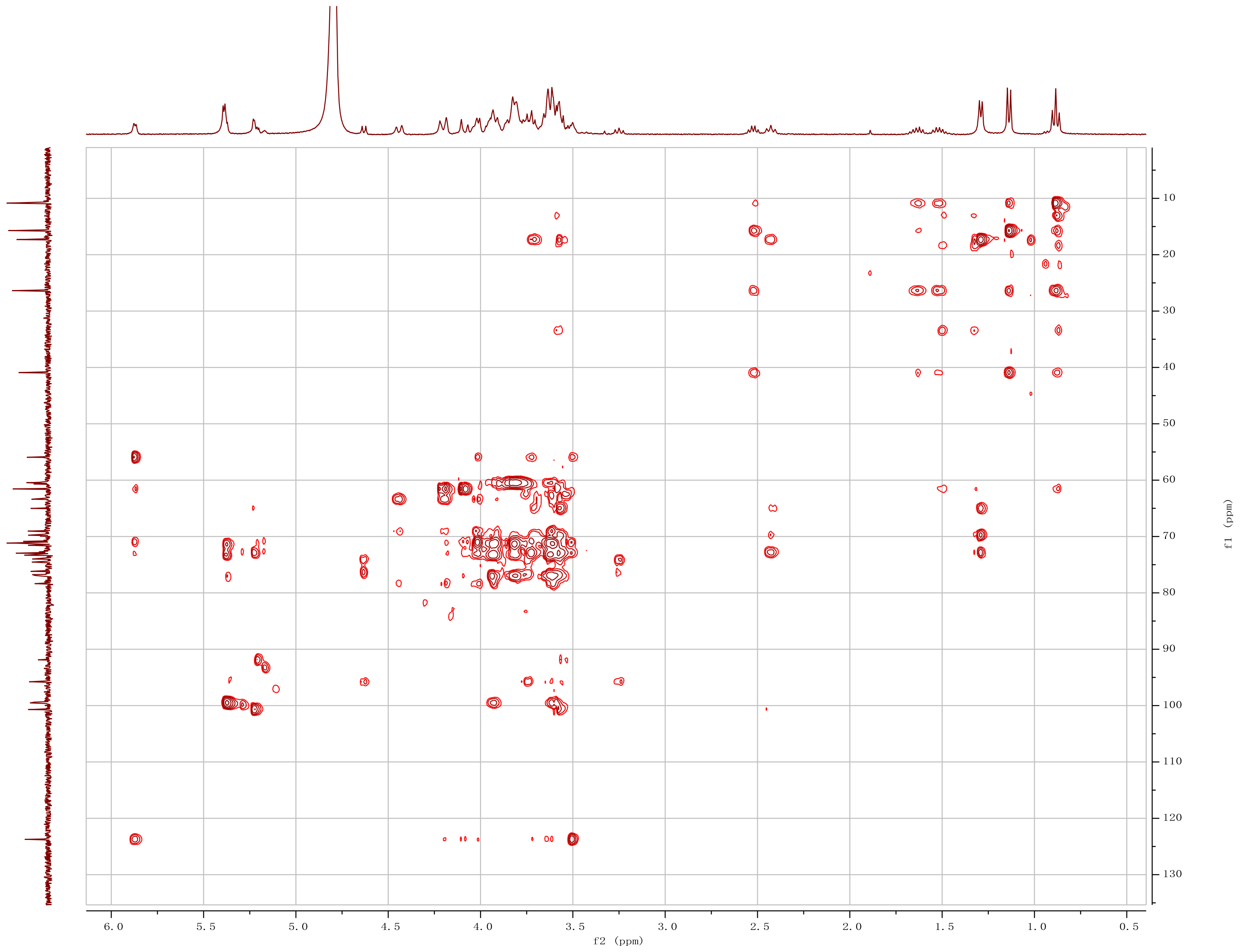

Figure S31. HSQC-TCOSY spectrum of compound **2** (500 MHz, D_2O).

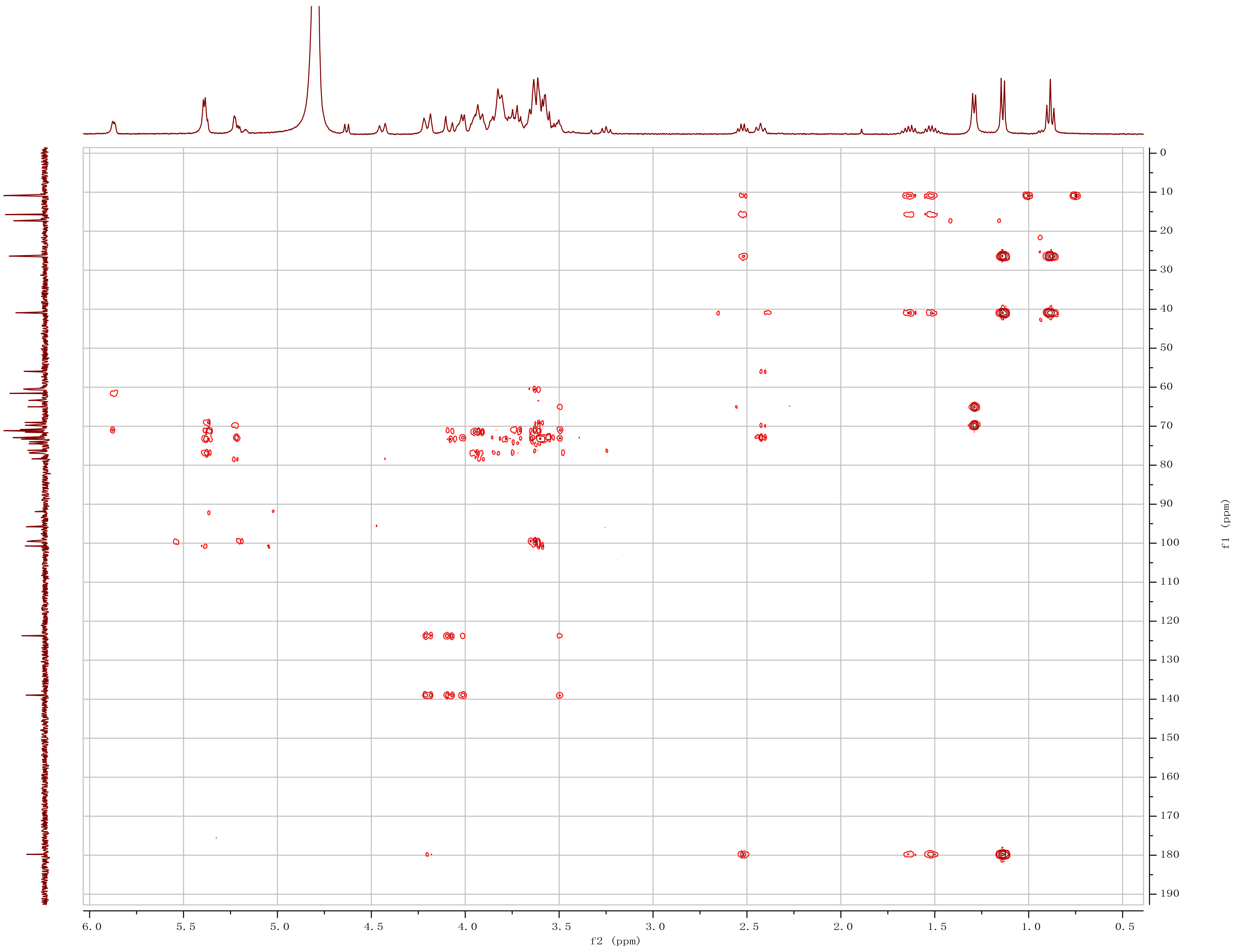

Figure S32. HMBC spectrum of compound **2** (500 MHz, D_2O).

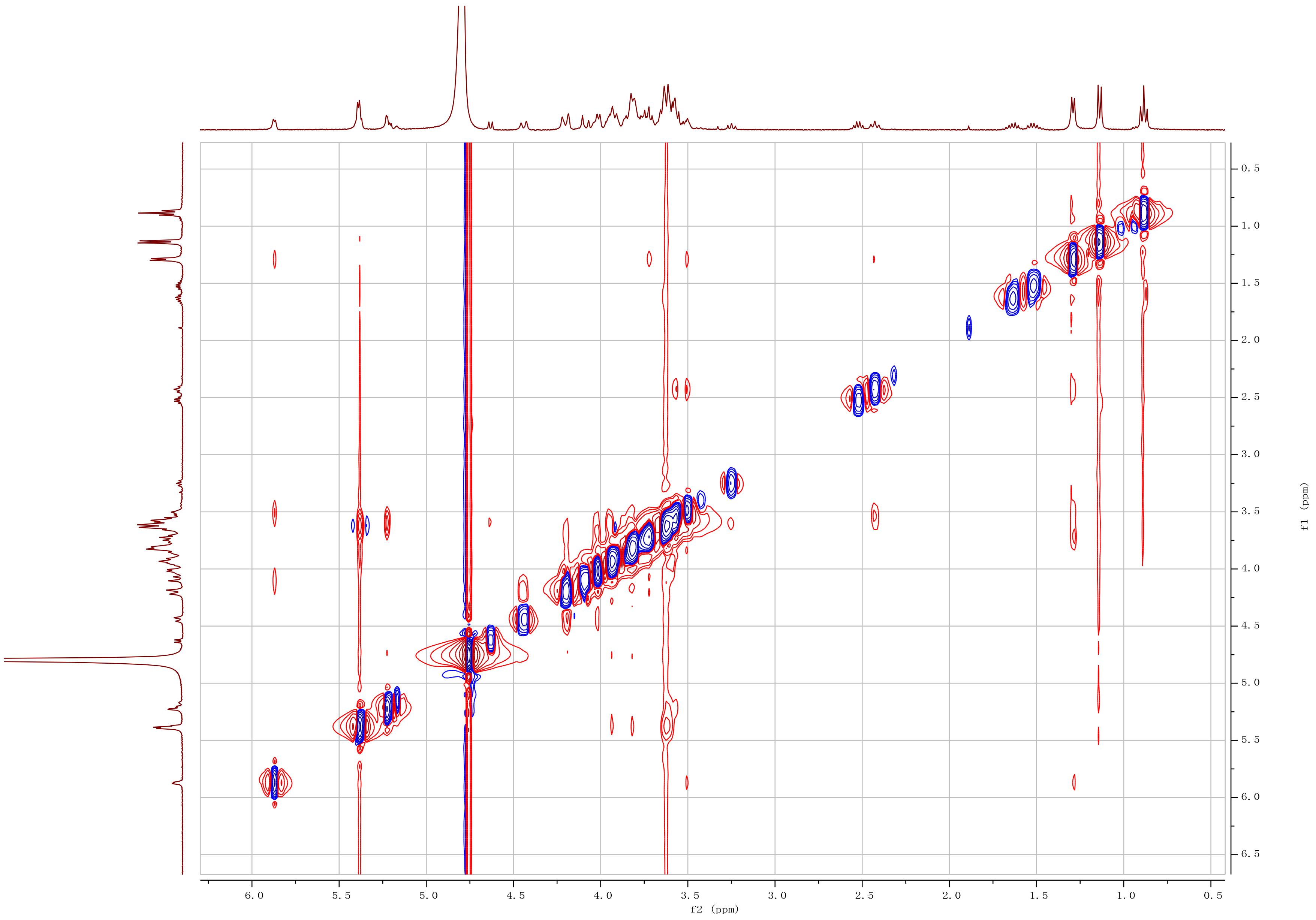

Figure S33. NOESY spectrum of compound **2** (500 MHz, D_2O).

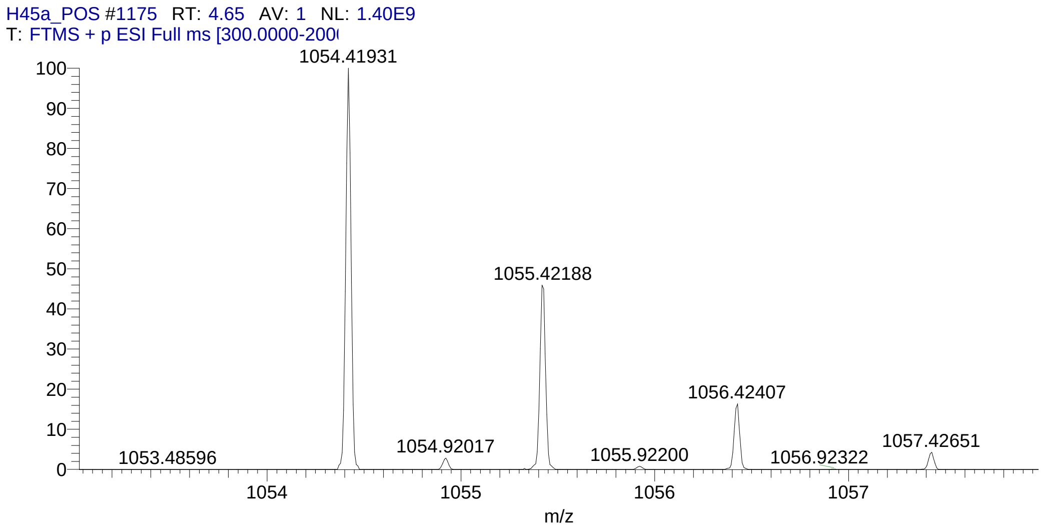

Figure S34. HRESIMS spectrum of compound **2**.

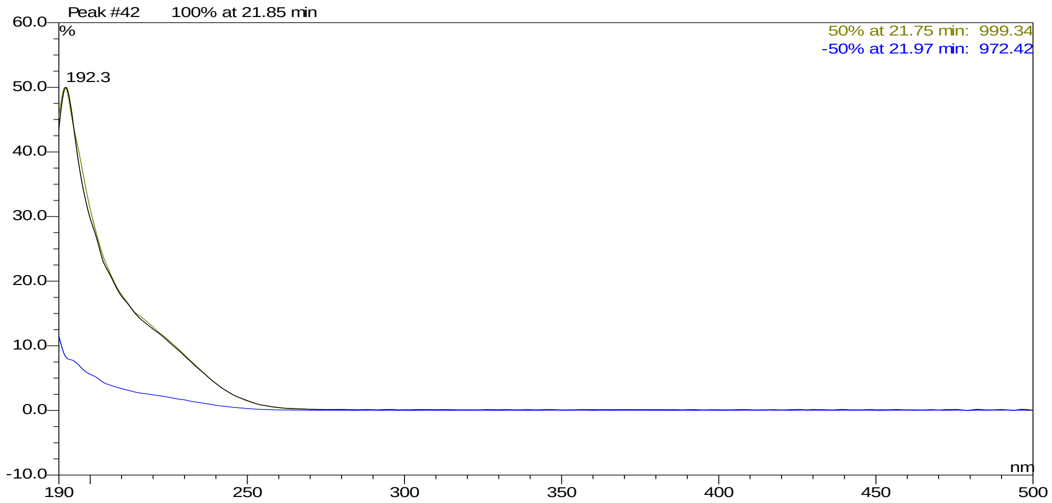

Figure S35. UV spectrum of compound **2**.

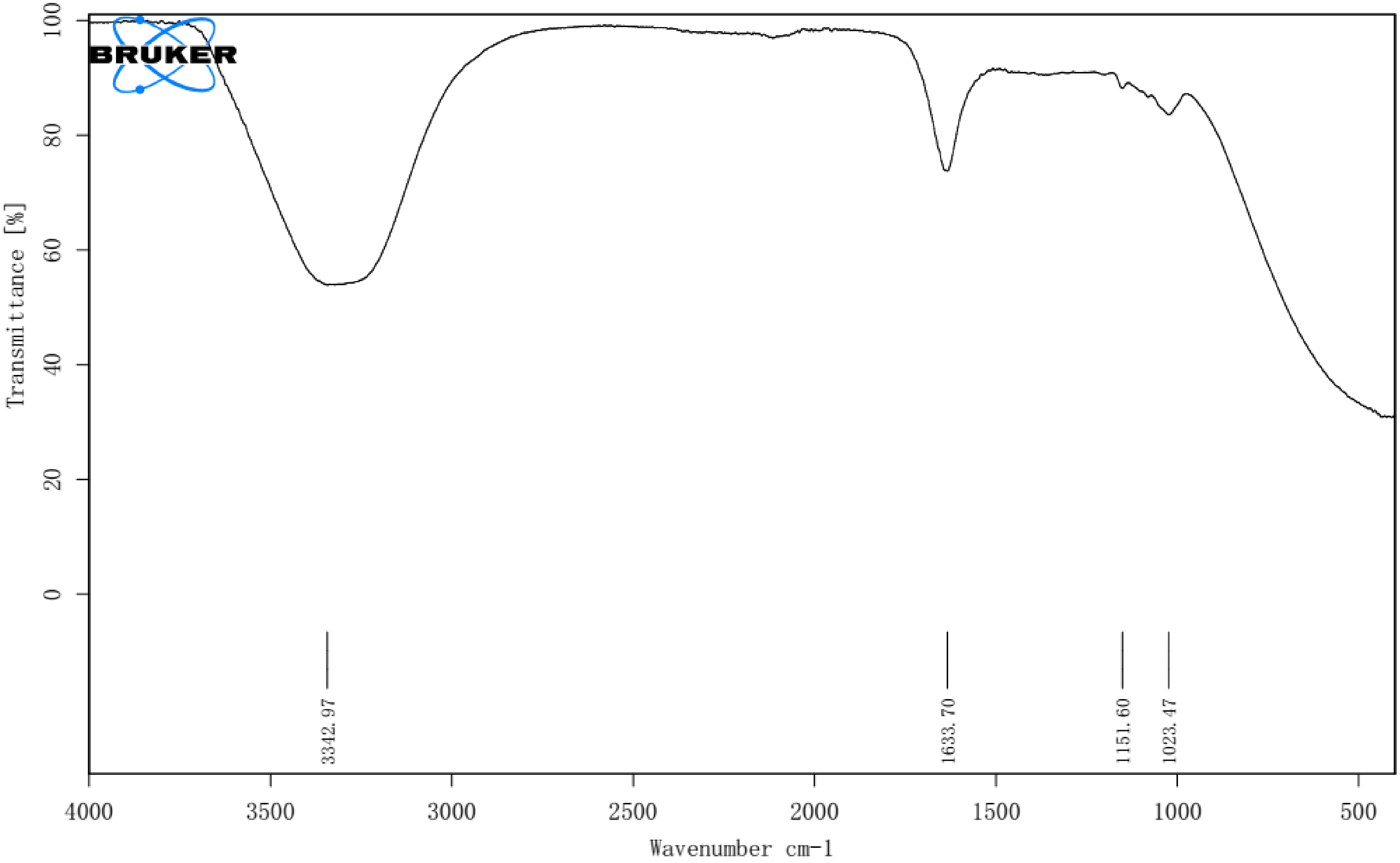

Figure S36. IR spectrum of compound **2**.

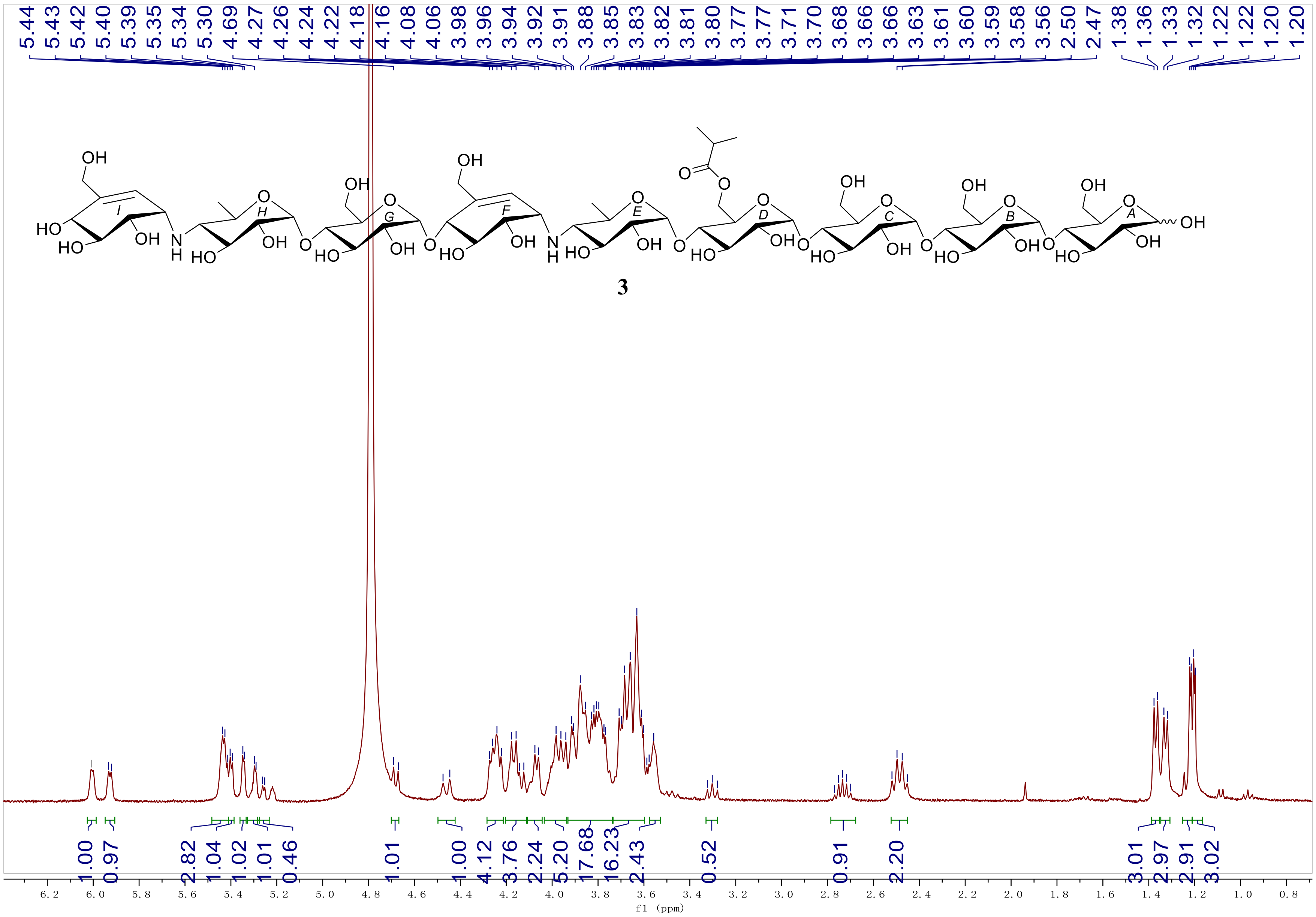

Figure S37. ^{1}H NMR spectrum of compound **1** (500 MHz, D_2O).

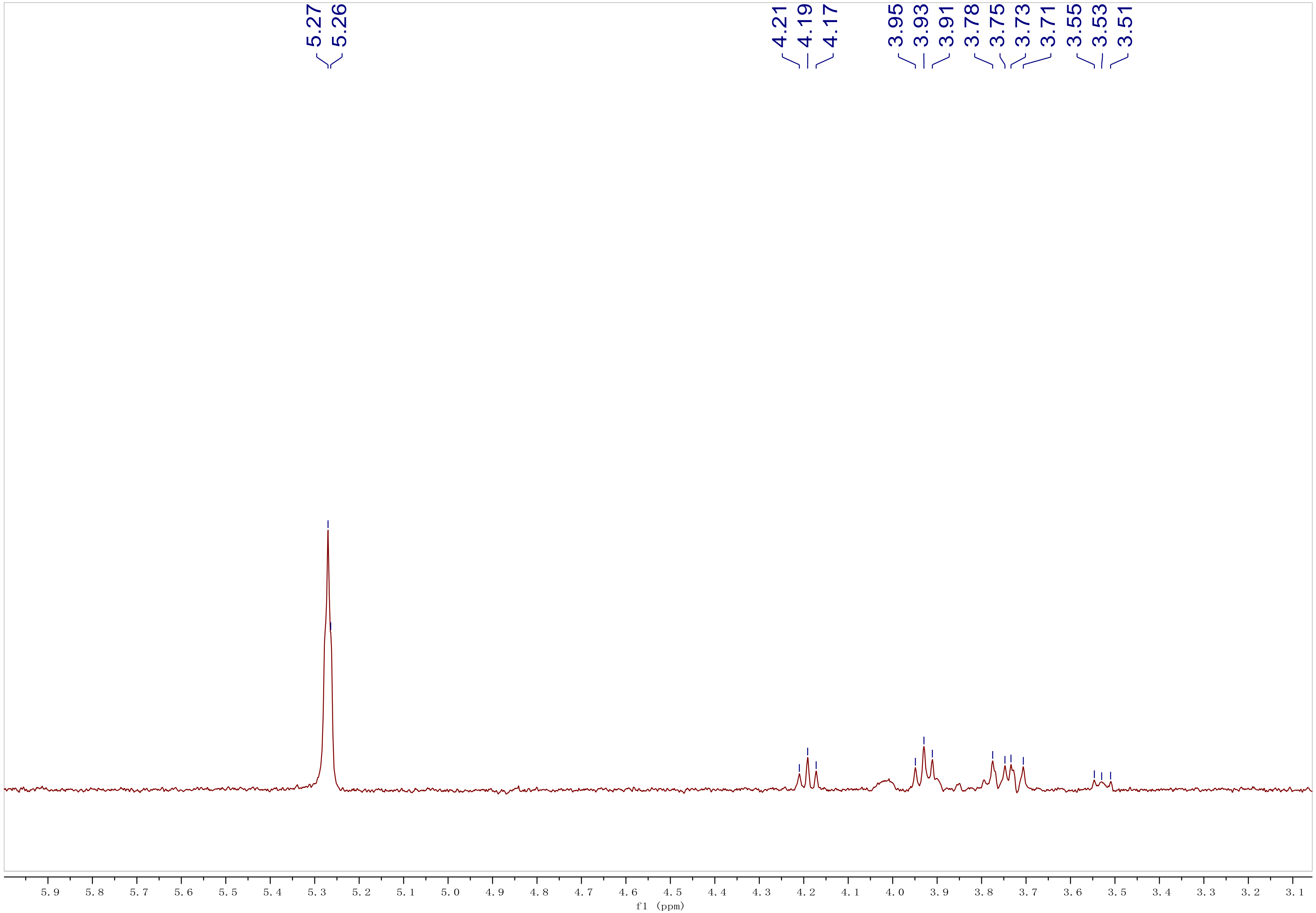

Figure S38. 1D-selective TOCSY spectrum of compound **3** (500 MHz, D_2O, excitation at δ 5.26, H-**A**1α).

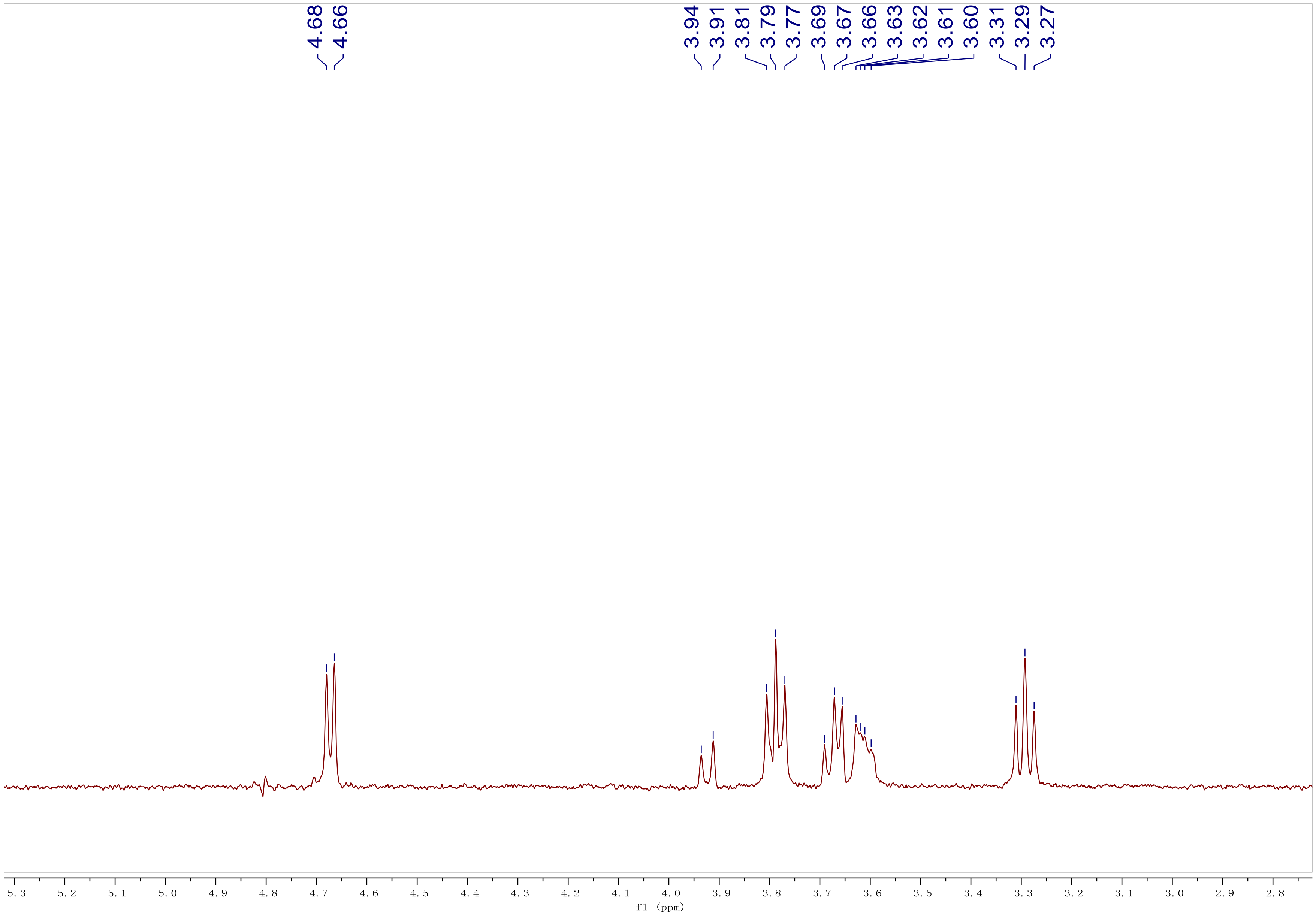

Figure S39. 1D-selective TOCSY spectrum of compound **3** (500 MHz, D_2O, excitation at δ 4.66, H-**A**1β).

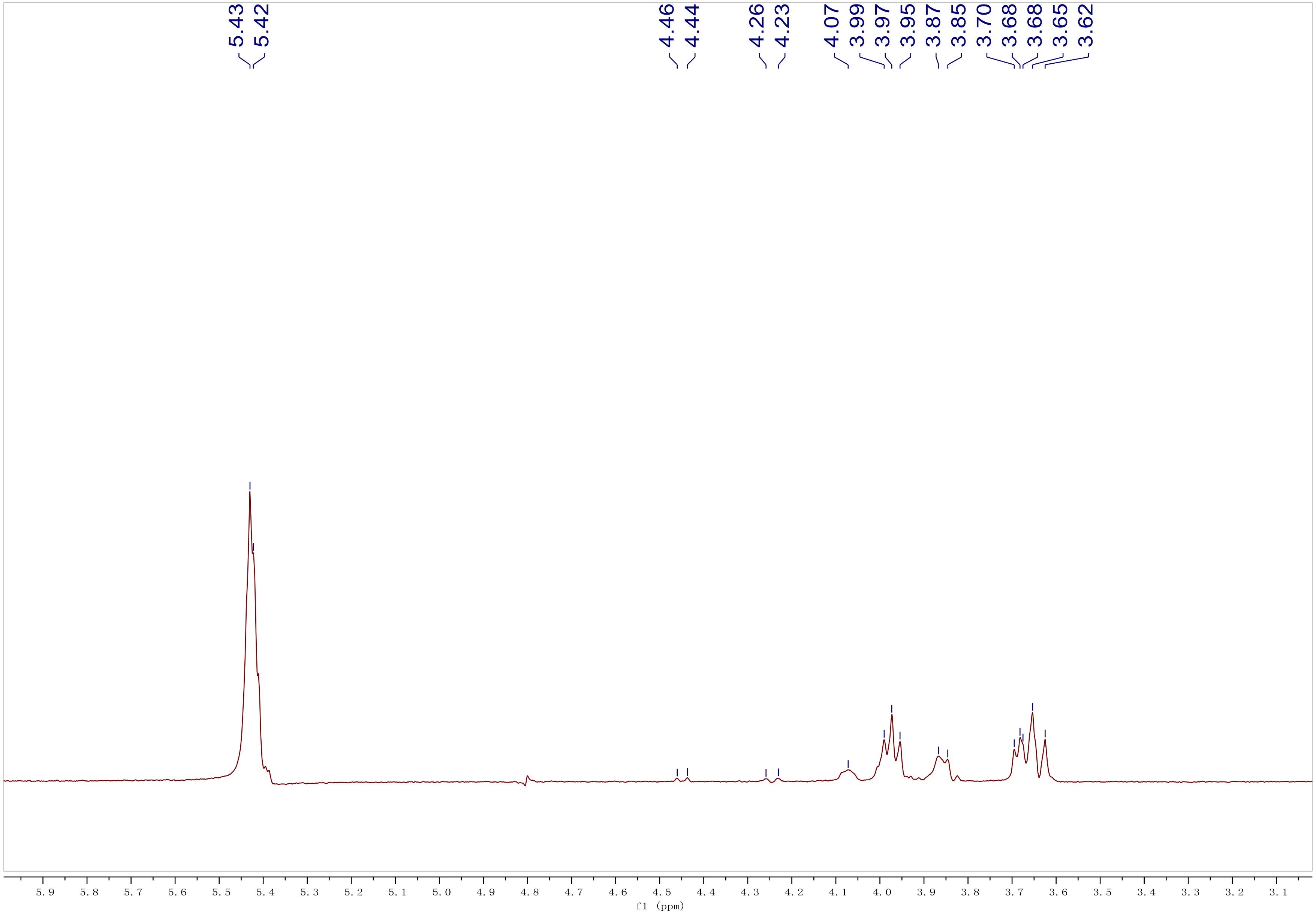

Figure S40. 1D-selective TOCSY spectrum of compound **3** (500 MHz, D_2O, excitation at δ 5.43, H-**B**1, H-**C**1, H-**D**1).

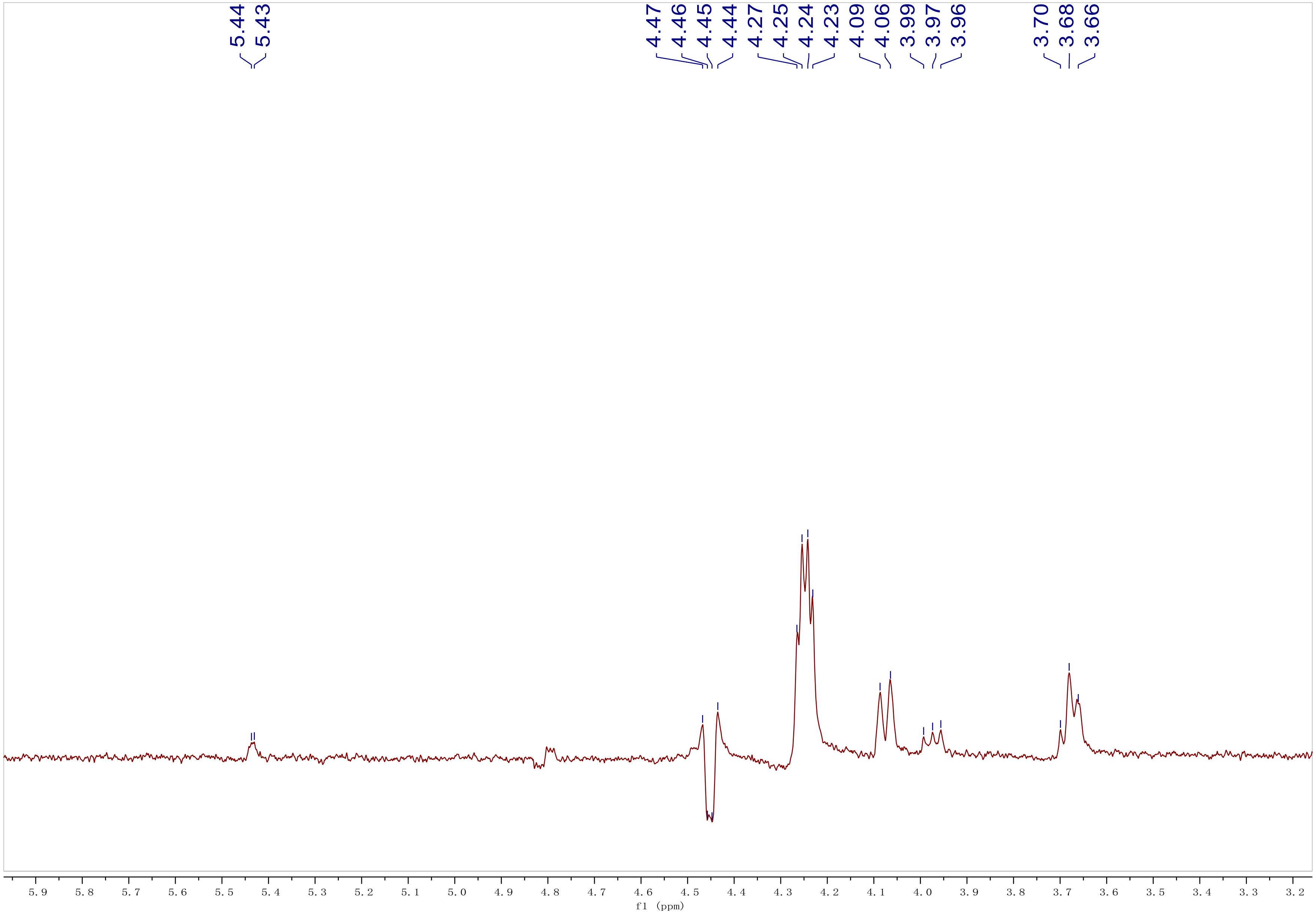

Figure S41. 1D-selective TOCSY spectrum of compound **3** (500 MHz, D_2O, excitation at δ 4.46, H-**D**6a).

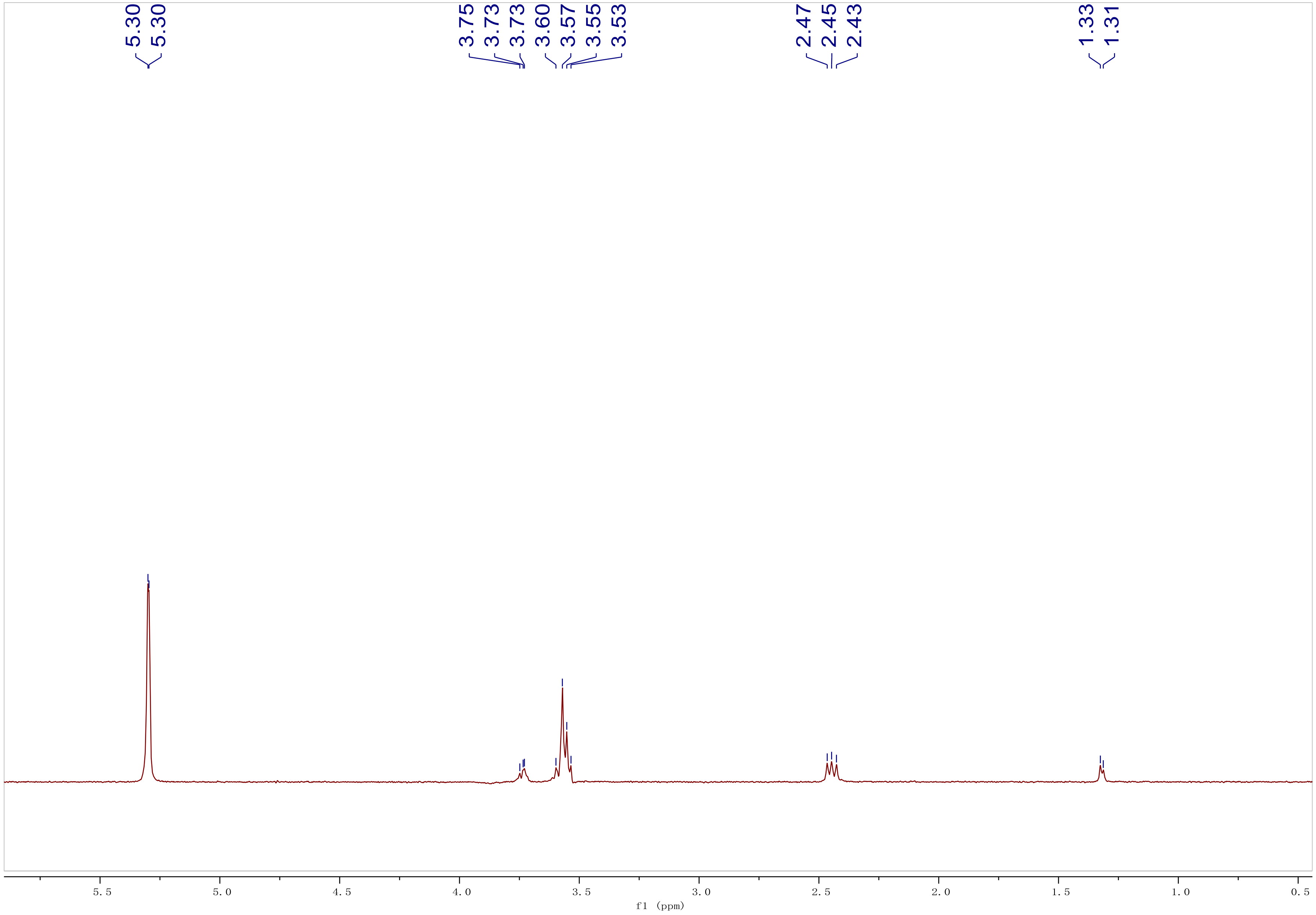

Figure S42. 1D-selective TOCSY spectrum of compound **3** (500 MHz, D_2O, excitation at δ 5.29, H-**E**1).

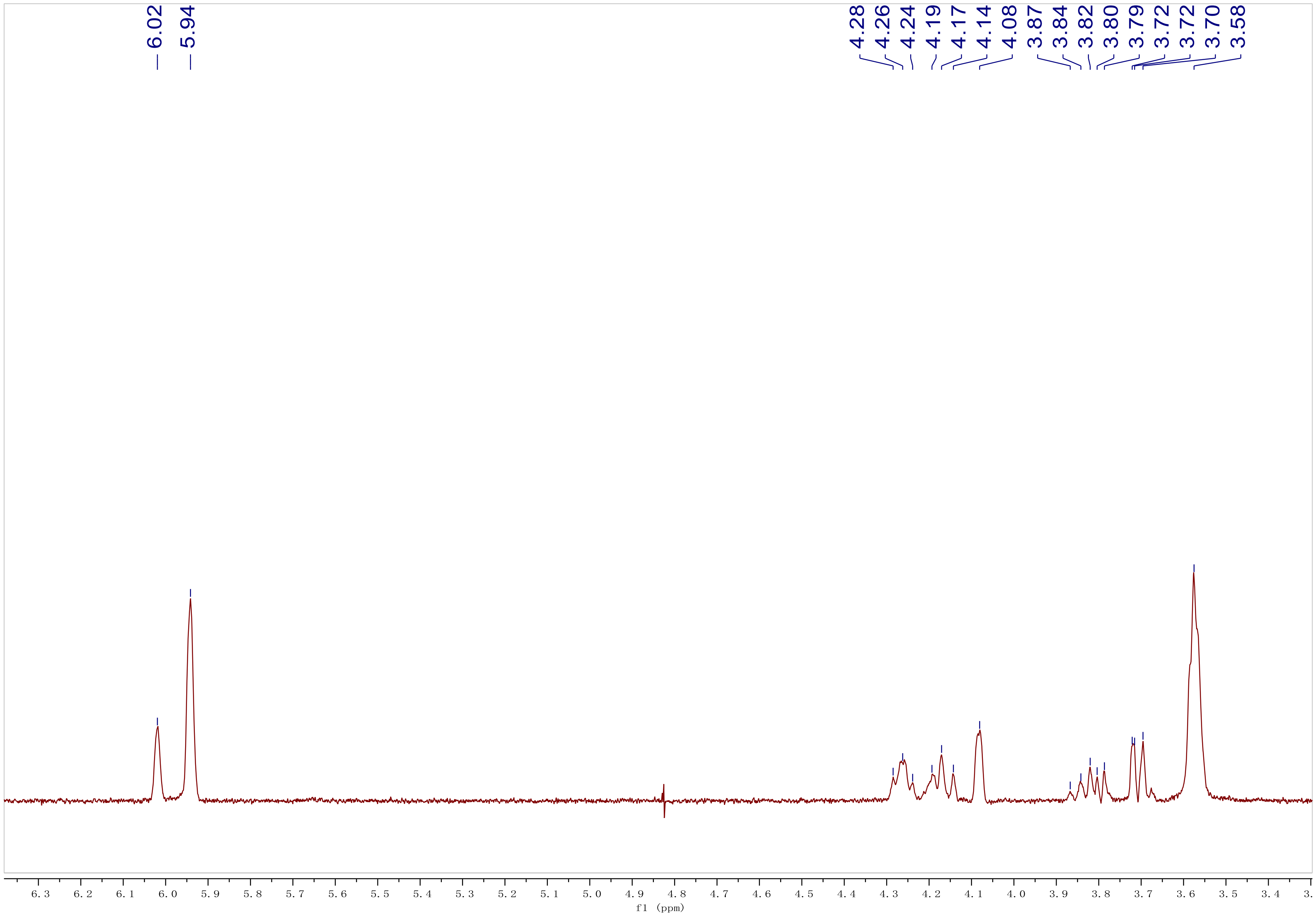

Figure S43. 1D-selective TOCSY spectrum of compound **3** (500 MHz, D_2O, excitation at δ 6.01, H-**F**7).

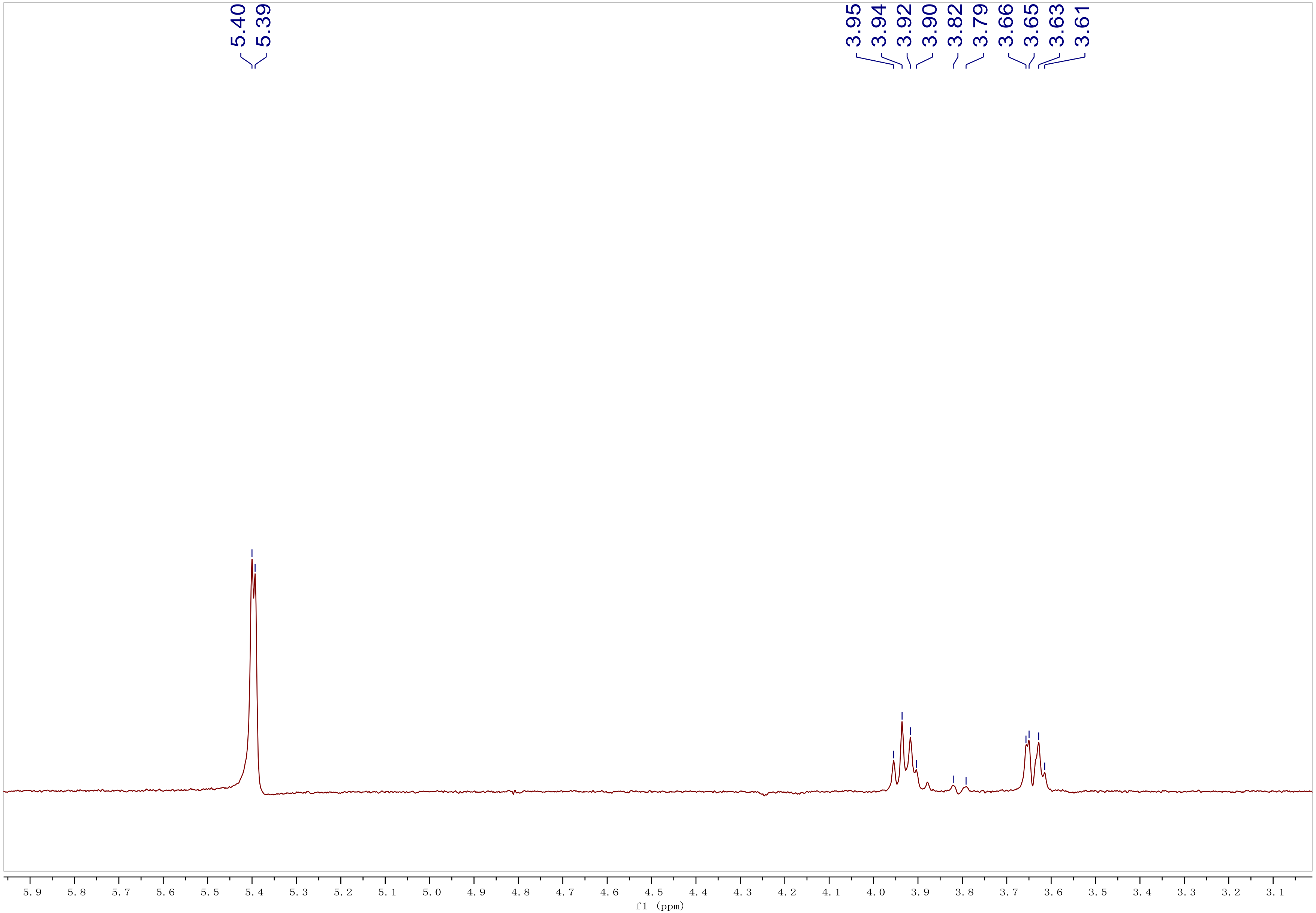

Figure S44. 1D-selective TOCSY spectrum of compound **3** (500 MHz, D_2O, excitation at δ 5.40, H-**G**1).

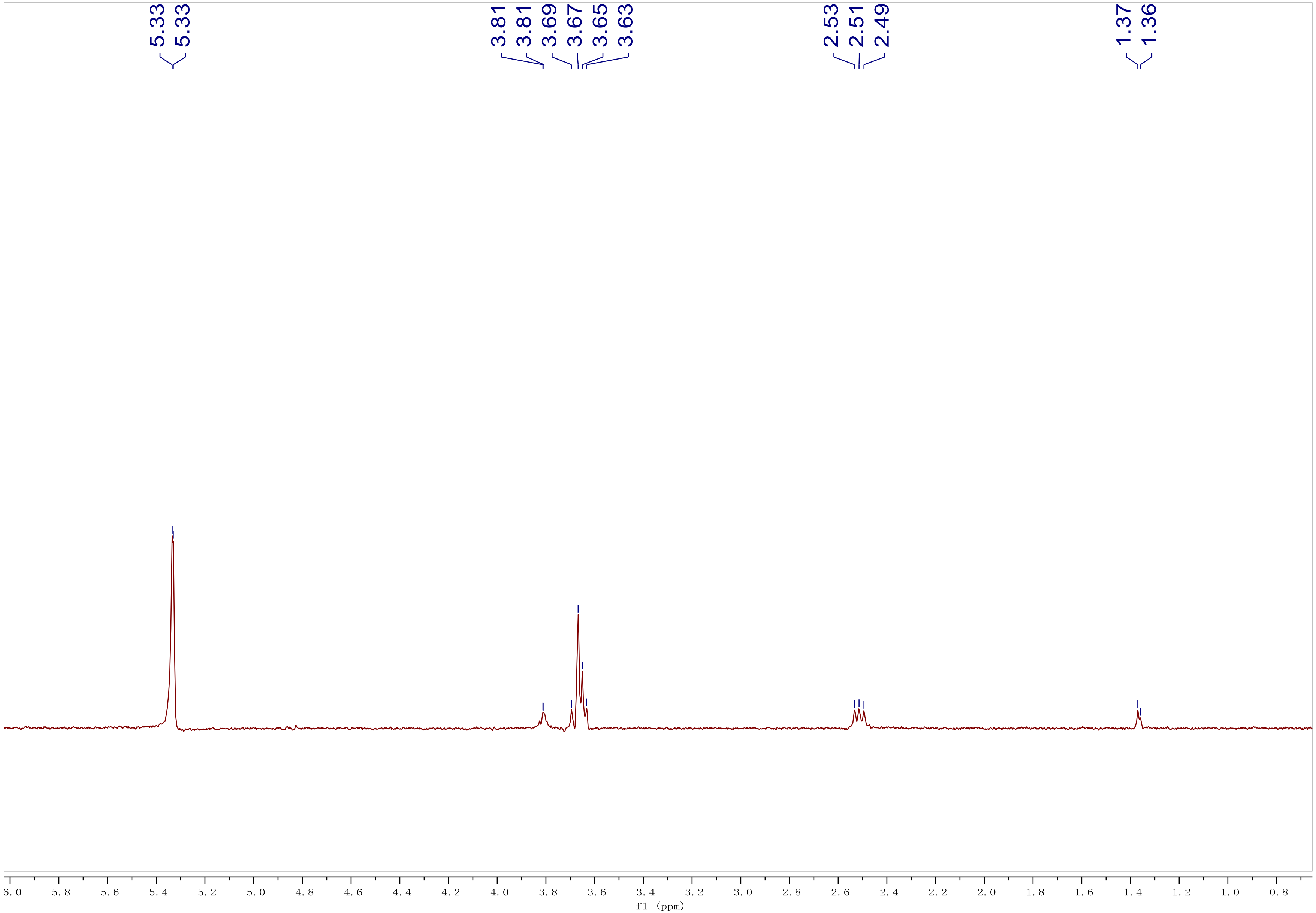

Figure S45. 1D-selective TOCSY spectrum of compound **3** (500 MHz, D_2O, excitation at δ 5.34, H-**H**1).

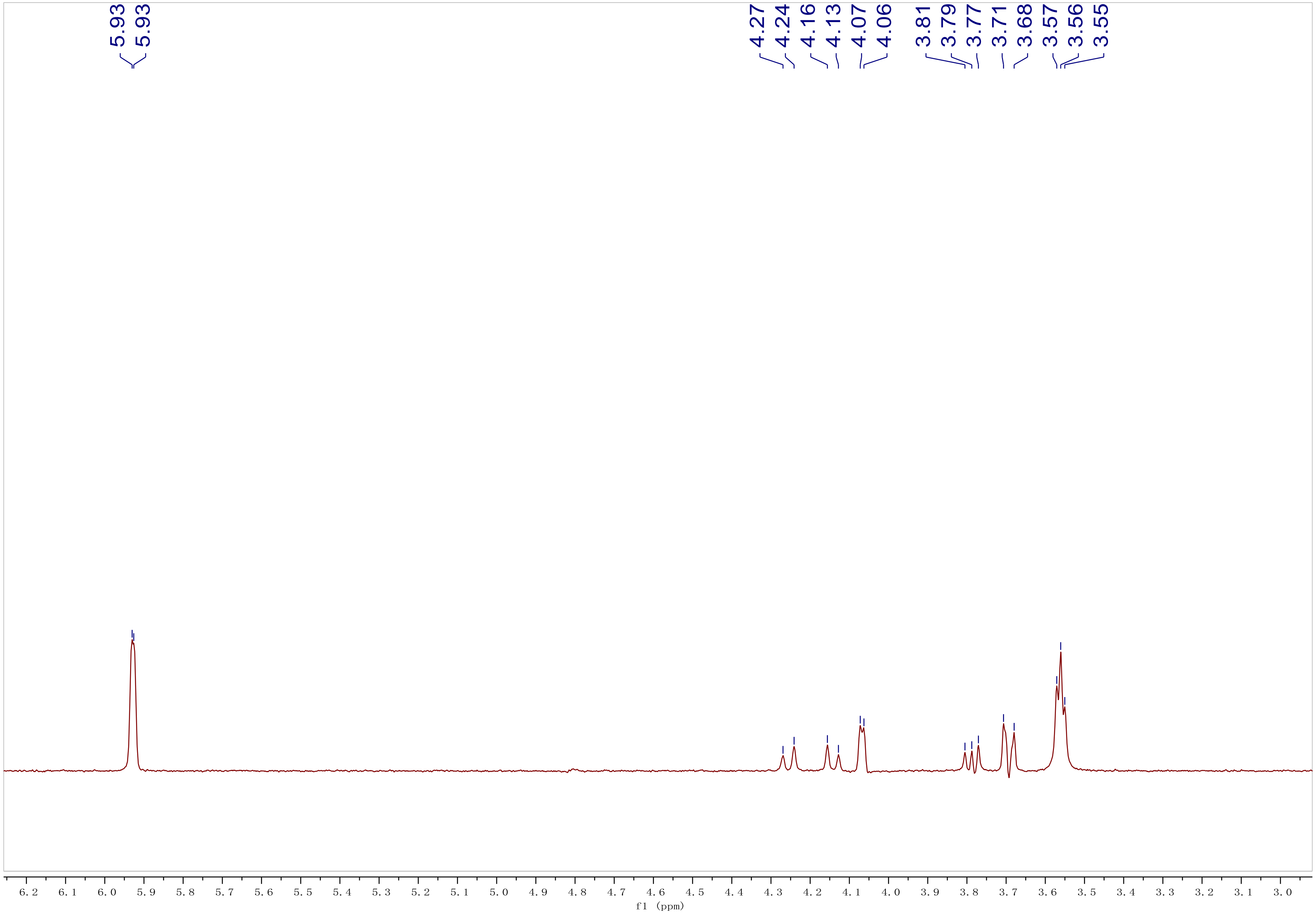

Figure S46. 1D-selective TOCSY spectrum of compound **3** (500 MHz, D_2O, excitation at δ 5.93, H-**I**7).

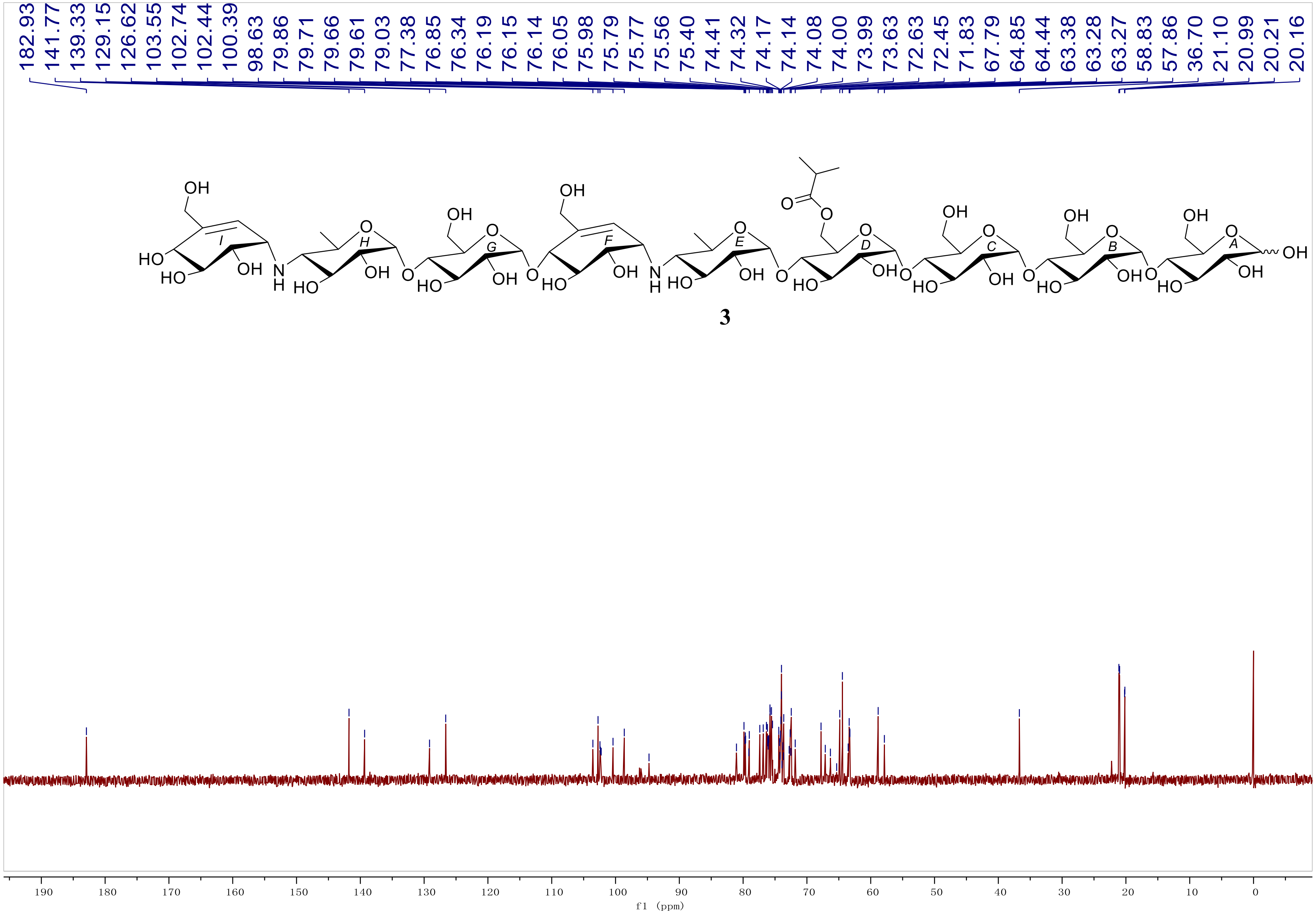

Figure S47. ^{13}C NMR spectrum of compound **3** (125 MHz, D_2O).

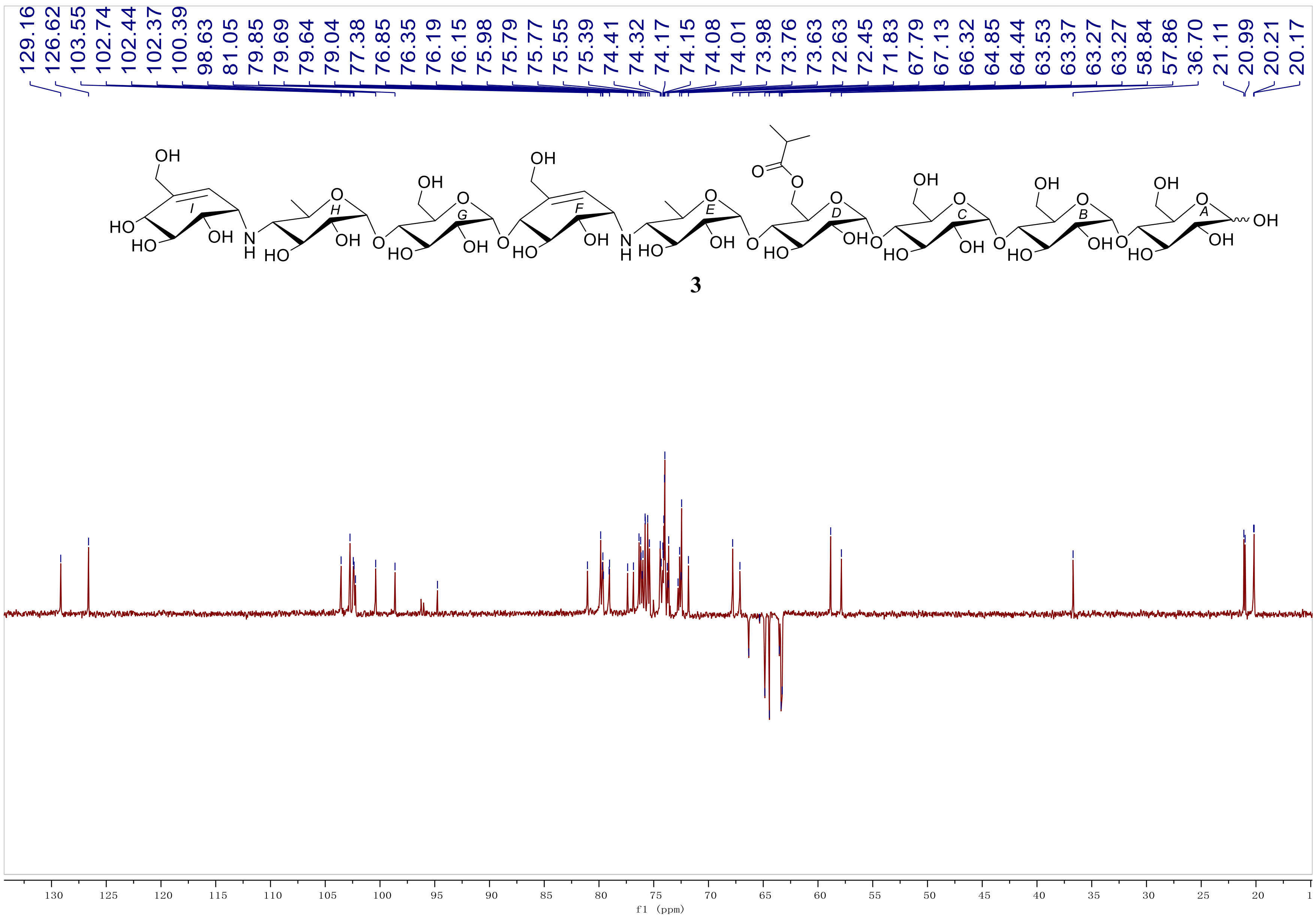

Figure S48. DEPT-135 spectrum of compound **3** (125 MHz, D_2O).

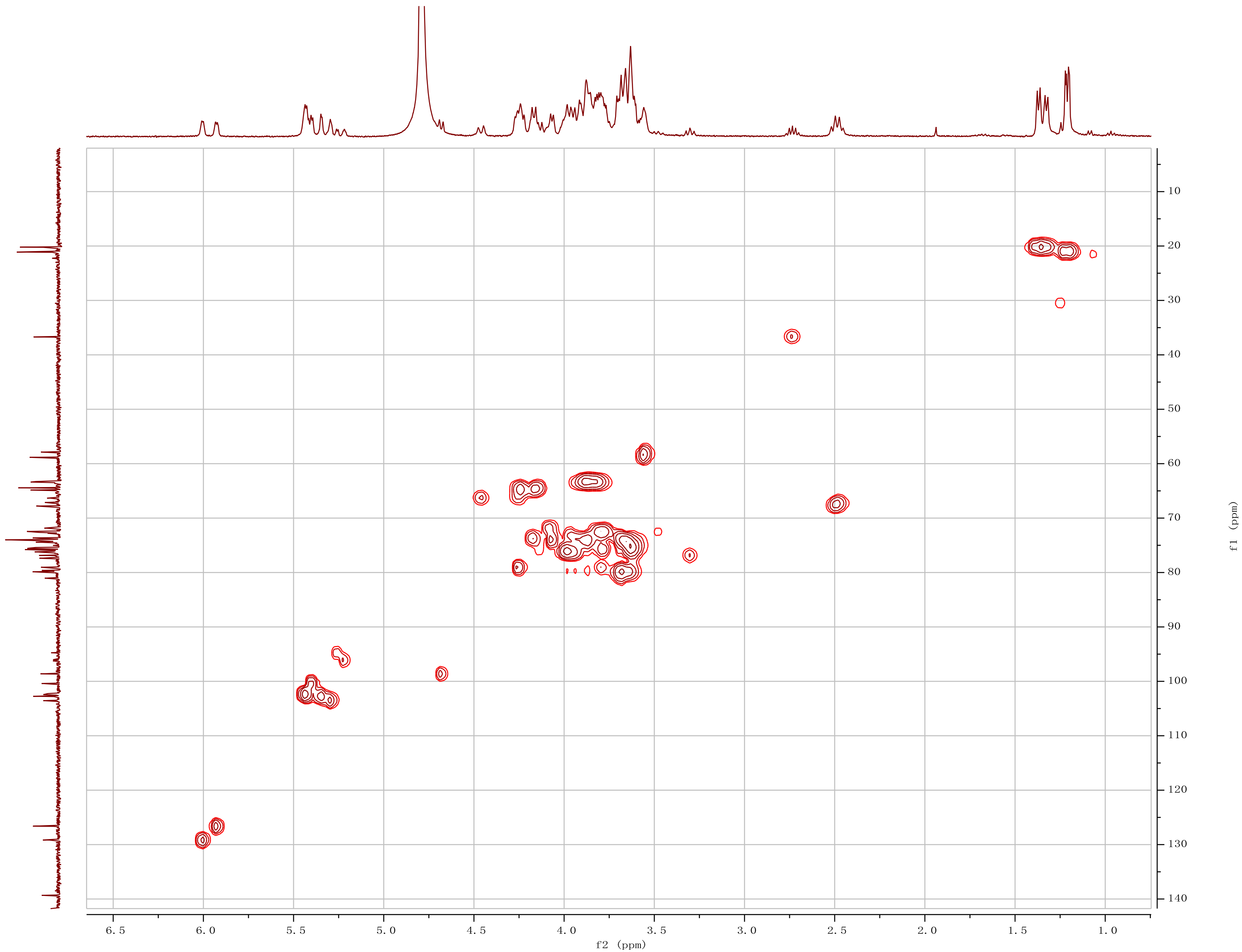

Figure S49. HSQC spectrum of compound **3** (500 MHz, D_2O).

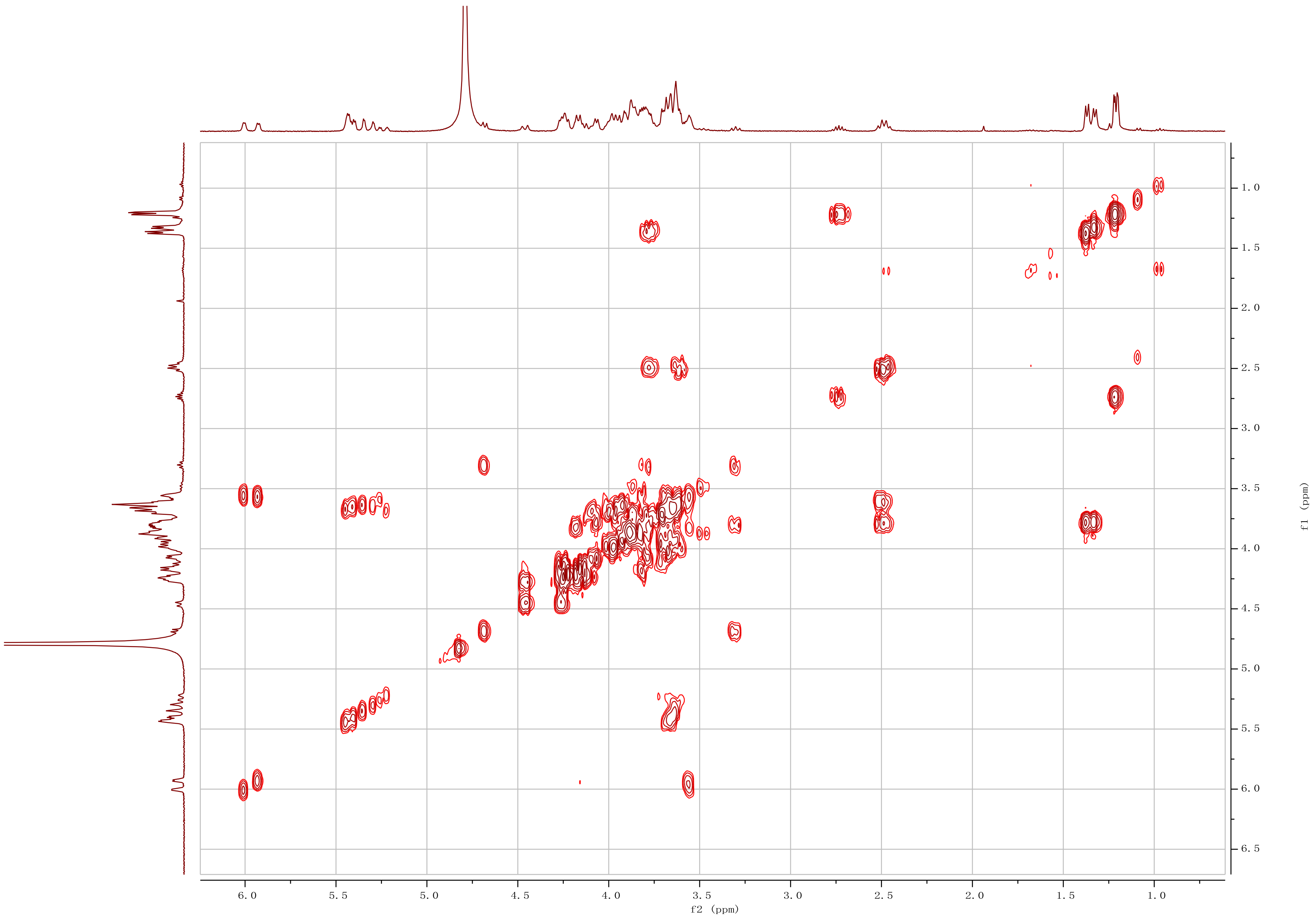

Figure S50. ^{1}H-^{1}H COSY spectrum of compound **3** (500 MHz, D_2O).

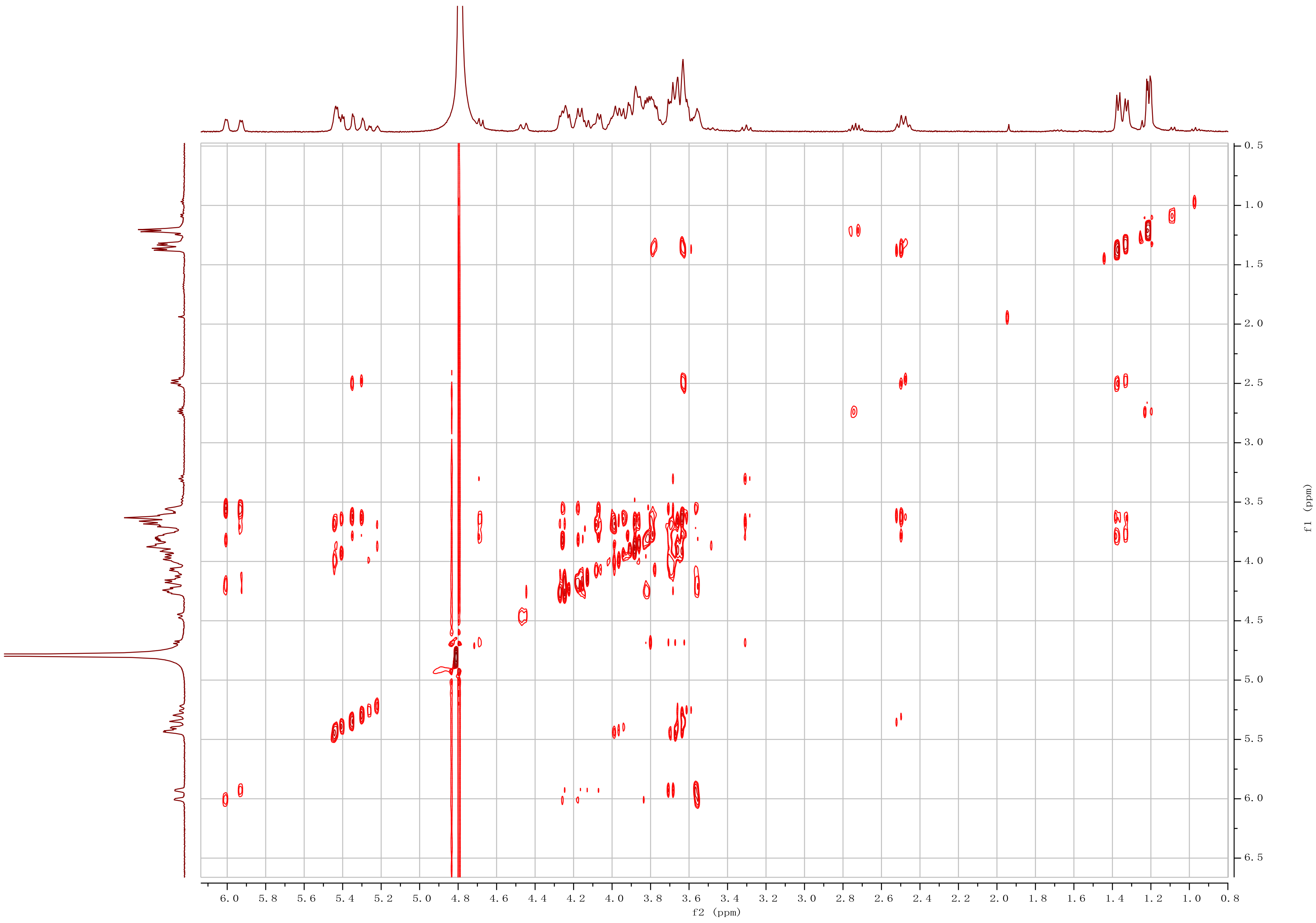

Figure S51. 2D-TOCSY spectrum of compound **3** (500 MHz, D_2O).

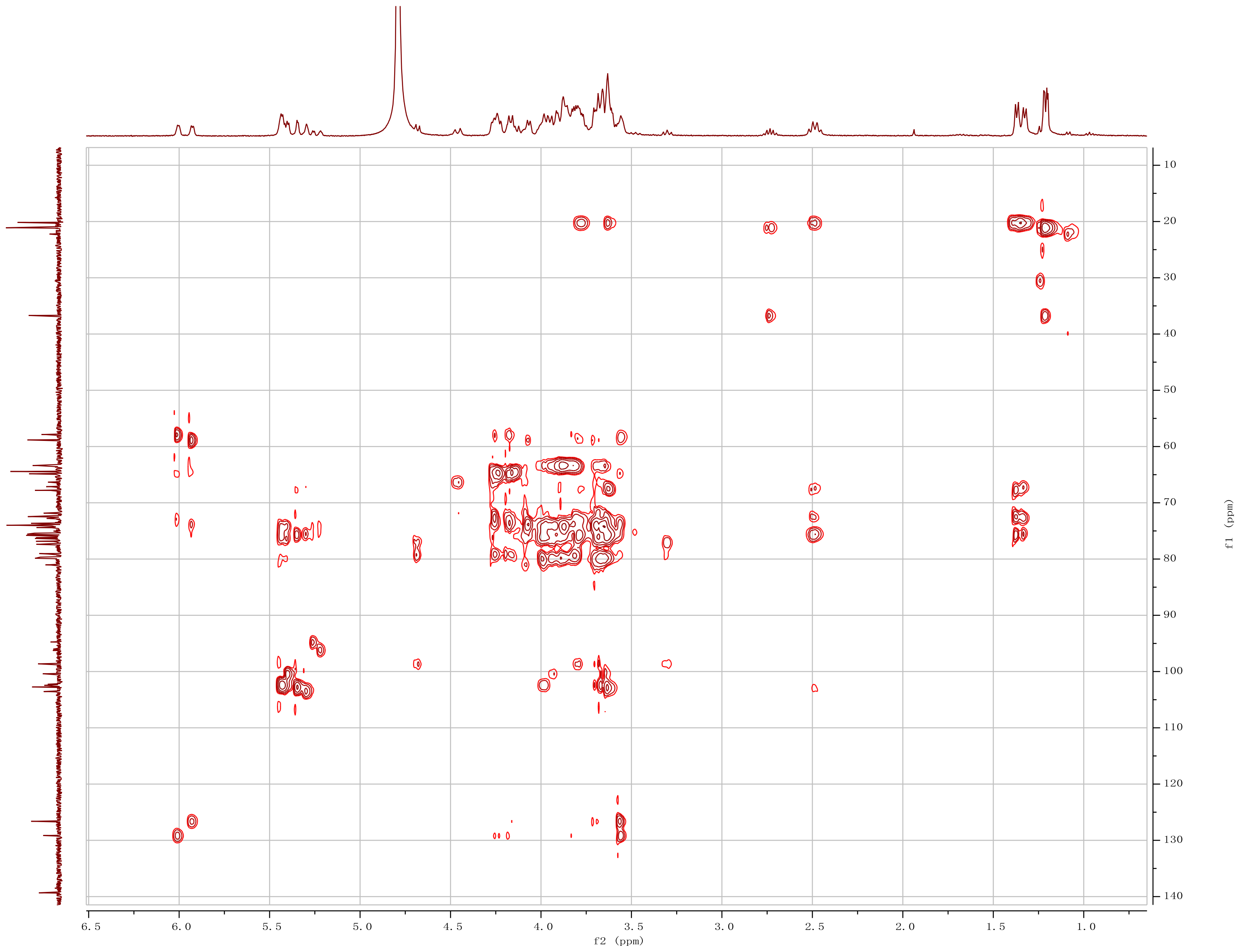

Figure S52. HSQC-TOCSY spectrum of compound **3** (500 MHz, D_2O).

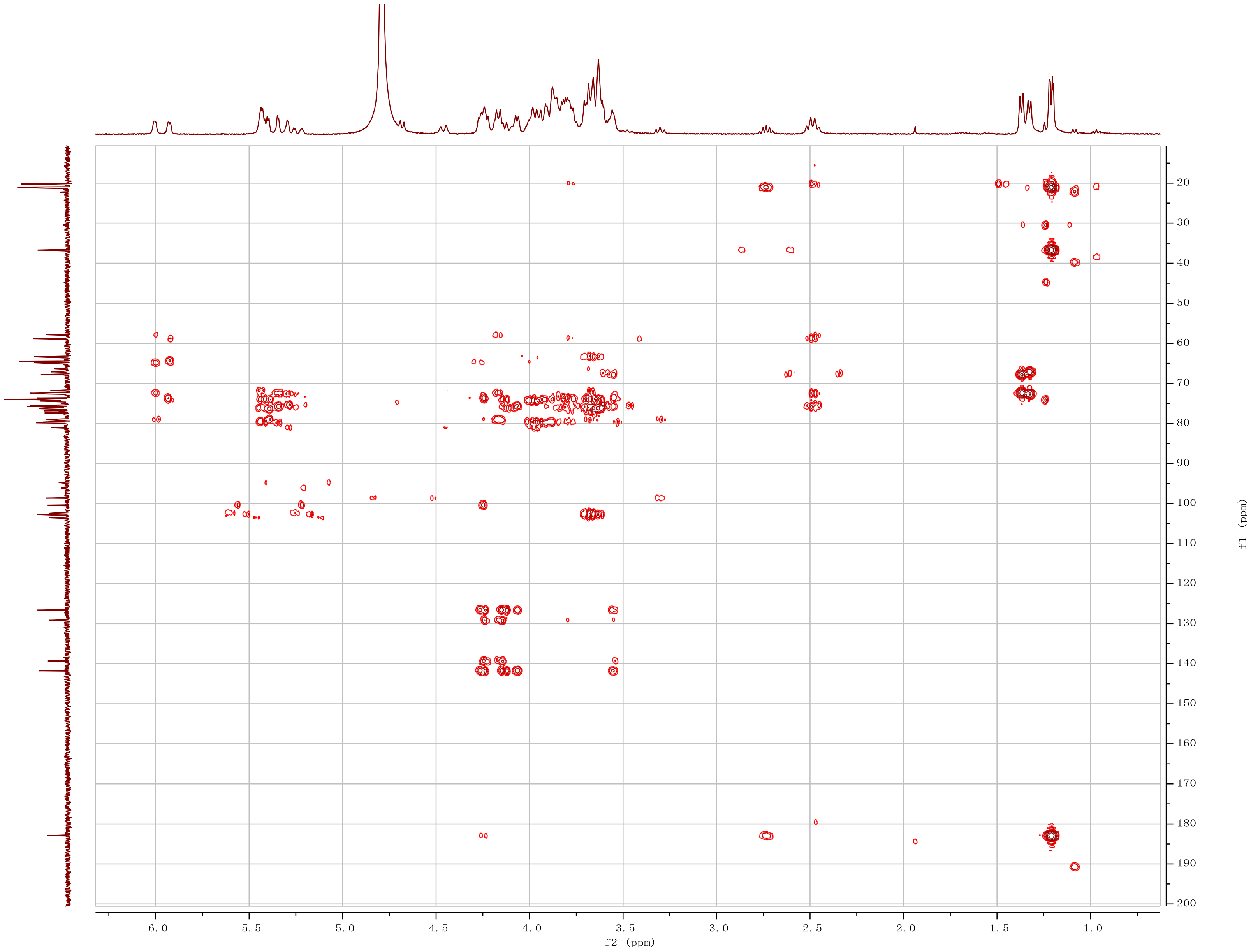

Figure S53. HMBC spectrum of compound **3** (500 MHz, D_2O).

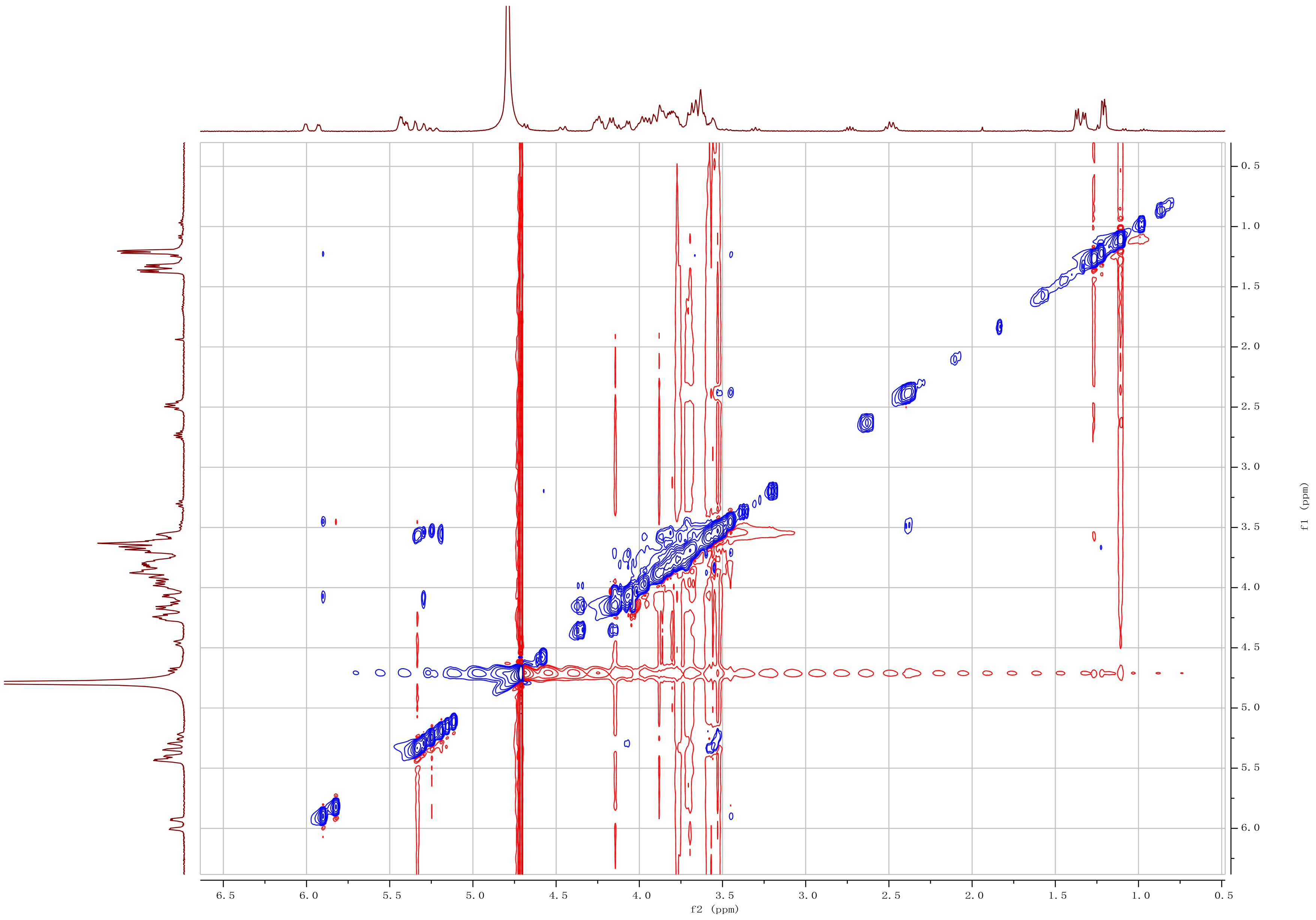

Figure S54. NOESY spectrum of compound **3** (500 MHz, D_2O).

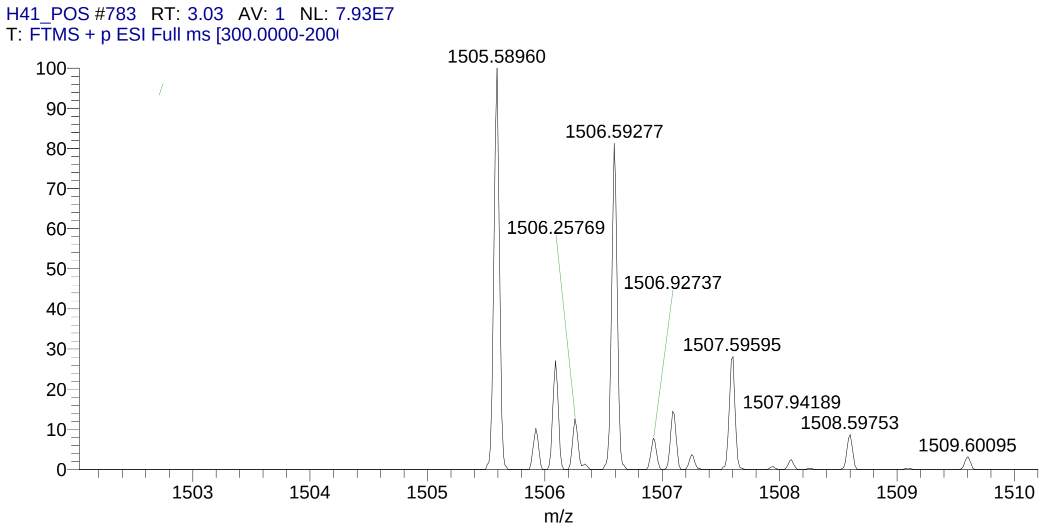

Figure S55. HRESIMS spectrum of compound **3**.

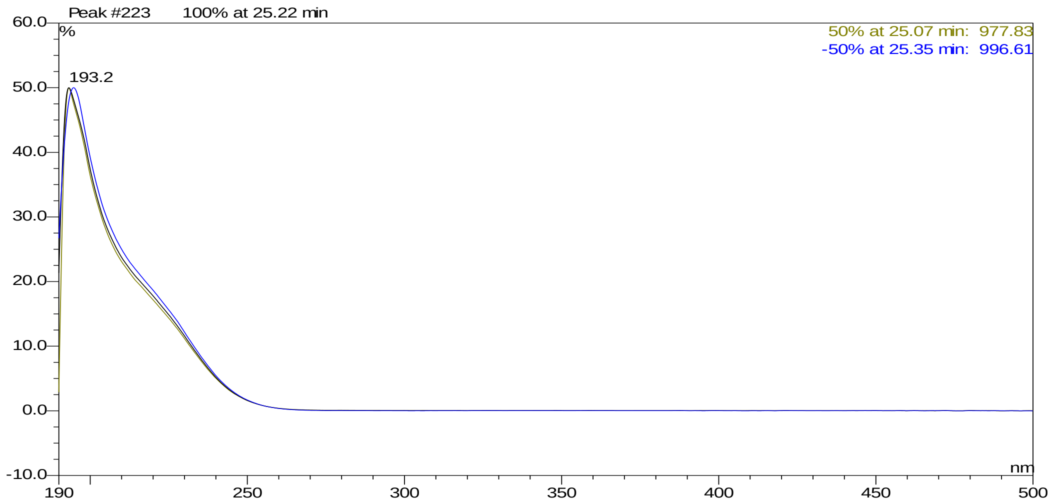

Figure S56. UV spectrum of compound **3**.

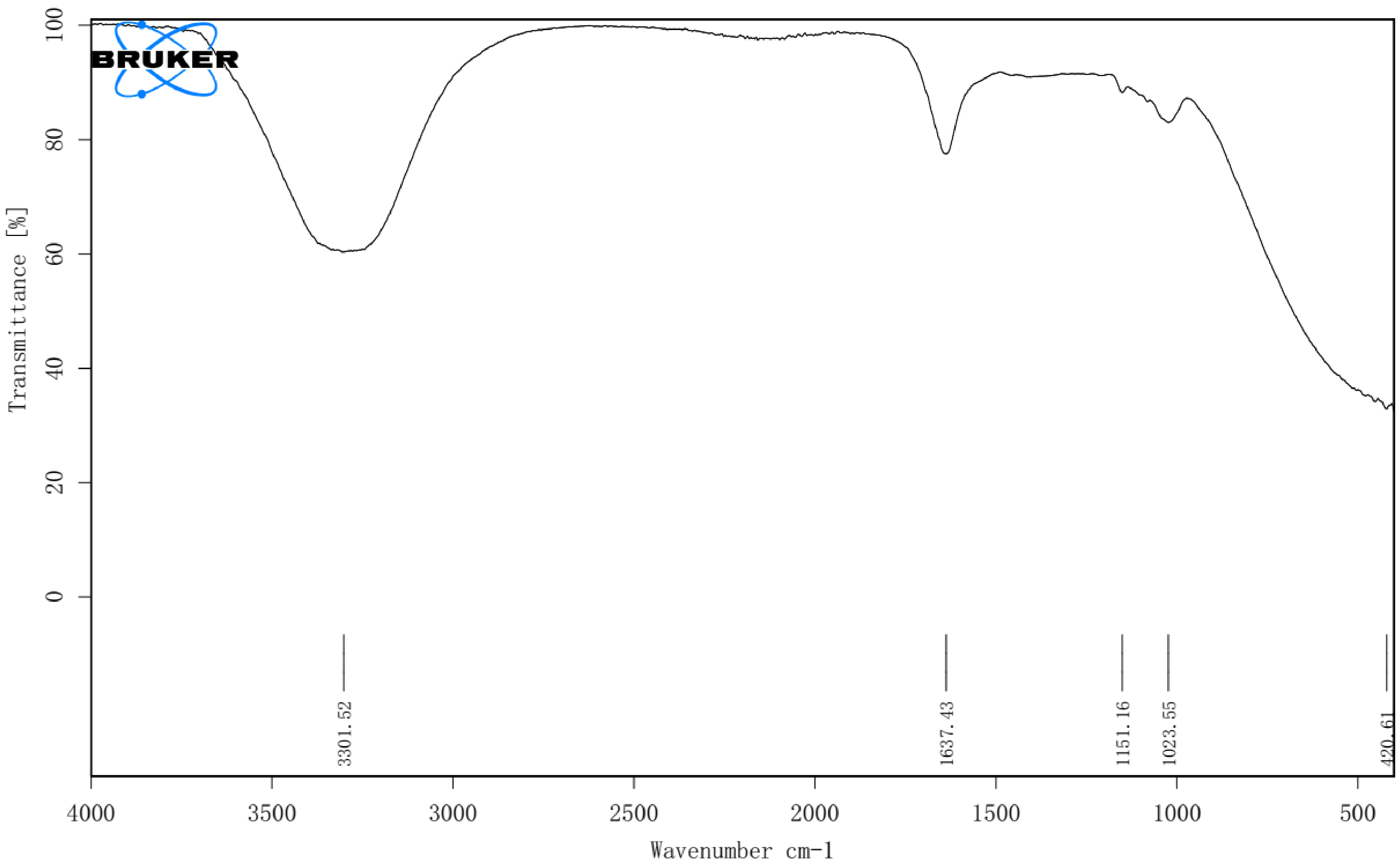

Figure S57. IR spectrum of compound **3**.

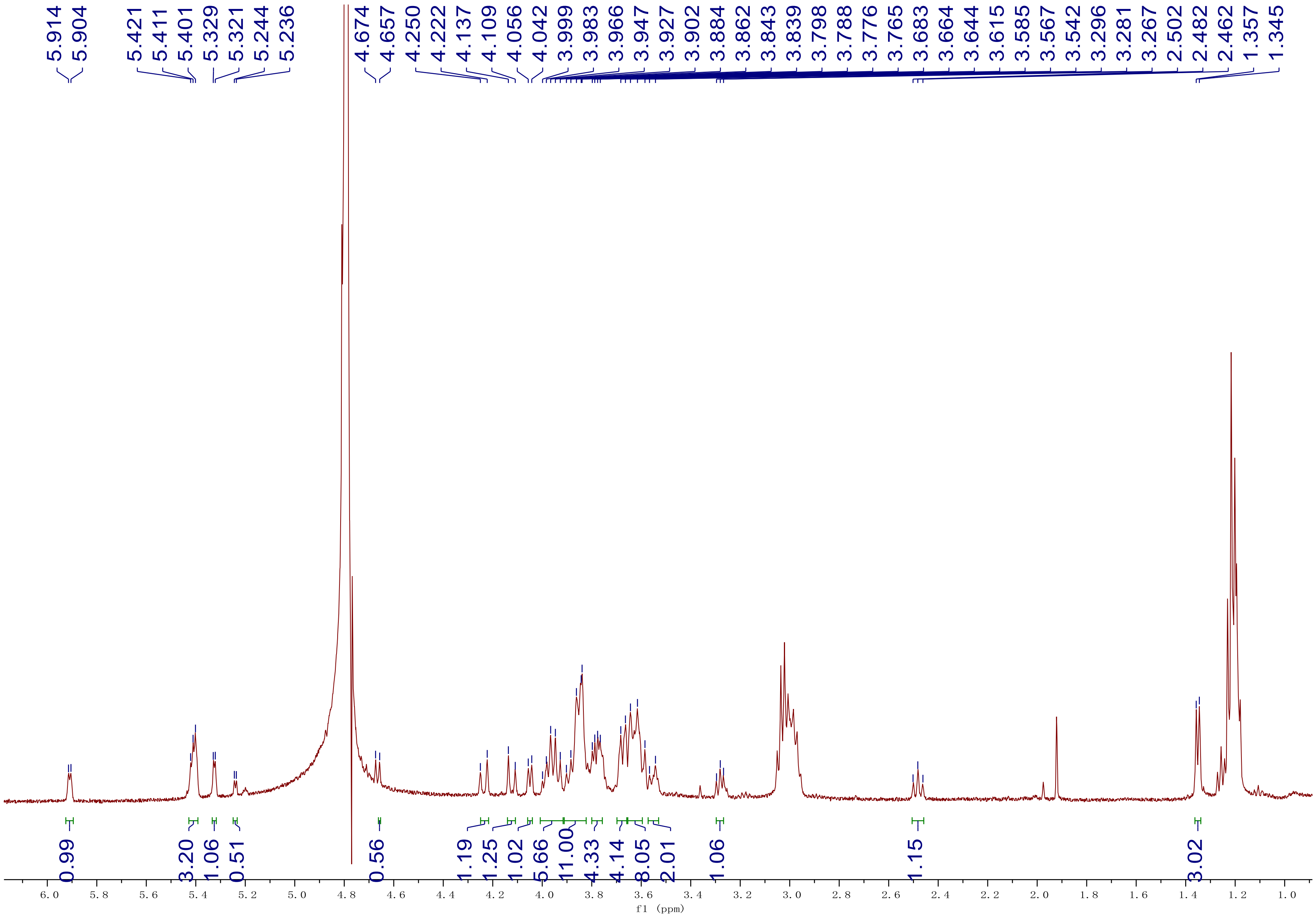

Figure S58. ^{1}H NMR spectrum of the common basic hydrolysis product (**9**) of compounds **1** and **5** (500 MHz, D_2O).

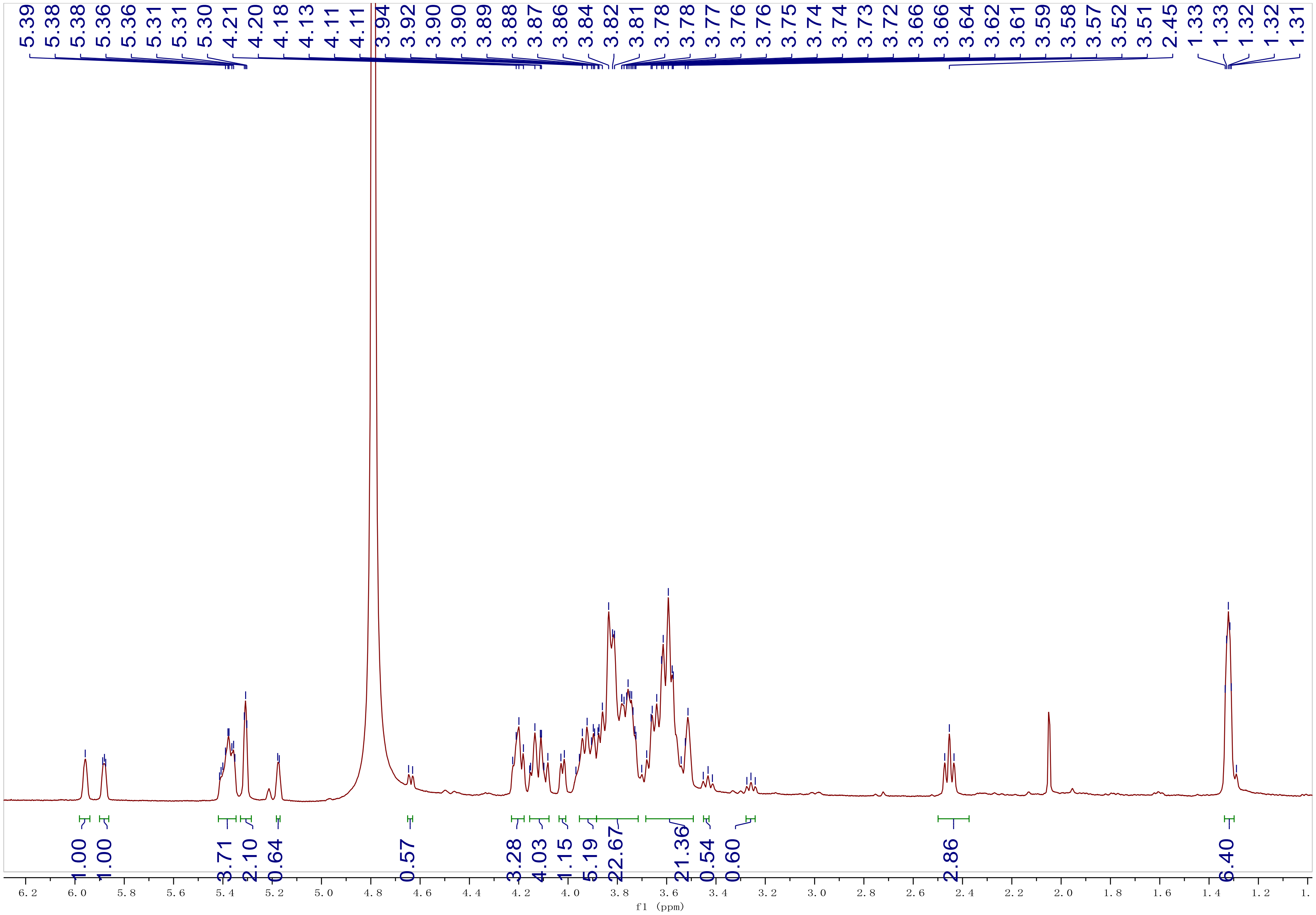

Figure S59. ^{1}H NMR spectrum of the common basic hydrolysis product (**10**) of compound **3** (500 MHz, D_2O).
